# Factors associated with individuals’ preferences for Primary Health Care facility type: A case of Bolgatanga Municipality in the Upper East Region of Ghana

**DOI:** 10.1371/journal.pone.0354800

**Published:** 2026-08-20

**Authors:** Charles Obeng, Sussana Atanga, Kofi Akohene Mensah, Lawal Alhassan

**Affiliations:** 1 Department of Health Policy, Management, and Economics, Kwame Nkrumah University of Science and Technology (KNUST), Kumasi, Ghana; 2 Bolgatanga Municipal Health Directorate, Reproductive and Child Health Unit, Bolga, Ghana; 3 Department of Health Policy, Management and Economics, Kwame Nkrumah University of Science and Technology (KNUST), Kumasi, Ghana; 4 Bolgatanga Municipal Health Directorate, Office of Municipal Director of Health Service, Bolga, Ghana; Fayetteville State University, UNITED STATES OF AMERICA

## Abstract

**Background:**

Research on the factors influencing patients’ preferences for facility type has expanded considerably. However, there is a recognizable gap regarding the specific determinants that drive preferences for different types of Primary Health Care (PHC) facilities. To address this gap, the study identified the key factors influencing individuals’ preferences for PHC facility type in the Bolgatanga Municipality, Upper East Region, Ghana.

**Methods:**

This study employed a two-stage cluster sampling method, and 398 residents in the Bolgatanga Municipality of the Upper East Region were surveyed with structured questionnaires acting as the primary data collection tool. Data was entered and analyzed using Stata Version 17.0 and SPSS Version 22.0 computer software. Frequencies, percentages, and multinomial logistic regression statistical analysis were employed in analyzing the filed data.

**Results:**

Quality-of-care (aOR = 2.89, 95% CI: 1.02–9.78, p = 0.033) was a significant determinant of individuals’ preference for PHC facility type. The study also found that accessibility-related factors such as distance and location (aOR = 3.42, 95% CI: 1.78–6.84, p = 0.001), transportation options (aOR = 4.01, 95% CI: 2.10–7.83, p < 0.001), waiting time (aOR = 1.21, 95% CI: 1.10–2.47, p < 0.001), appointment availability (aOR = 2.31, 95% CI: 1.14–5.61, p = 0.001) and cost of care (aOR = 0.88, 95% CI: 0.02–0.95, p < 0.001) are associated with individuals’ preferences for PHC facility type.

**Conclusion:**

The study established perceived quality-of-care and accessibility factors significantly influenced individual preferences for PHC facility type, with cost-sensitive and nearby Community-Based Health Planning and Services (CHPS) compounds remaining crucial for clients with limited resources. Therefore, the management of facilities within Bolgatanga Municipality and similar contexts should improve service quality, reduce waiting times, and enhance transport and financial accessibility to ensure equitable utilization of PHC services.”.

## Introduction

Globally, Primary Health Care (PHC) has been a crucial component of the healthcare system in many countries. This level of healthcare delivery functions as the first point of contact between patients and the health system, and plays a significant role in ensuring access to essential health services [1]. According to the World Health Organization (WHO) [2], Primary Health Care (PHC) is a whole-of-society approach to health that aims to ensure the highest possible level of health and well-being and their equitable distribution by focusing on populations’ needs as early as possible along the continuum from health promotion and disease prevention to treatment, rehabilitation, and palliative care, as close as feasible to people’s everyday environment. PHC facilities consist of a different array of settings, such as community health centers, clinics, health posts, and general practitioner practices, which function as the frontline of healthcare delivery [3]. Global declarations, such as the Alma-Ata Declaration of 1978 and the Astana Declaration of 2018, unequivocally underscore the international commitment to PHC as the most equitable, effective, and efficient path to health [2].

PHC is the most inclusive, equitable, cost-effective, and efficient approach to enhancing people’s physical and mental health and social well-being. For instance, WHO [2] asserted that scaling up primary health care (PHC) interventions across low and middle-income countries could save 60 million lives and improve average life expectancy by 3.7 years by 2030. An estimated 75% of the projected health gains from the Sustainable Development Goals could be achieved through PHC [2,4]. In low-middle-income countries (LMICs) like Rwanda, community health worker programs and health posts have optimized the transformative power of PHC that has significantly improved access to basic care and health outcomes in rural areas [5]. Also, Tanzania, through the Health Basket Fund channeled to the PHC facilities has focused on strengthening its primary care infrastructure and improving service delivery at the district level, recognizing PHC as key to decentralized health governance and equitable access [6]. Similarly, in Ghana, the Community-Based Health Planning and Services (CHPS) compounds, representing the first contact of the health system, are designed to bring preventive, promotive, and basic curative services directly to users and communities, especially in rural areas, thereby improving access to immunizations, maternal and child health services, and common disease management [7]. Ghana has in previous years explicitly adopted PHC principles aimed at improving health at the basic level through its National Health Insurance Scheme (NHIS). This NHIS aims to reduce financial barriers to care, particularly at the primary care level. [8]. This approach aligns with PHC’s emphasis on community participation and ownership, fostering local engagement in health planning and delivery. These regional efforts demonstrate how PHC enhances health system resilience, enabling better preparedness and response to public health emergencies, as evidenced by adaptations made during the COVID-19 pandemic across the continent [9].

PHC delivery effectiveness is dependent on the efficient use of diverse facility types. And this functions as the bedrock for universal health coverage and improved health outcomes for the population [7,10]. However, patients do not consistently utilize the nearest or most appropriate PHC facility despite the investments by member states to expand PHC infrastructure and services, particularly in LMICs like Ghana. Rather, their healthcare-seeking behavior reflects deliberate preferences for specific clinic or hospital types, often bypassing proximate facilities in favor of more distant ones [7,10]. In Ghana, the number of outpatient consultations in PHC has not increased significantly, and patients still seek medical care at secondary or tertiary hospitals rather than PHC institutions as their first point of contact, leading to excessive demand and a burden on secondary and tertiary hospitals, while underutilizing PHC institutions [11]. For instance, in the Upper West Region of Ghana, a study by Farhan and Aryeetey [12] found a 77.8% utilization rate of secondary and tertiary healthcare compared to a 22.2% utilization rate for PHC, suggesting a considerable wastage of high-cost medical resources in the secondary and tertiary hospitals. This can easily result in prolonged waiting time for patients and excessive workloads for doctors and other healthcare providers. Research asserts that this preference or health-seeking behavior is influenced by an intricate interplay of various factors [7].

Studies demonstrate that patients’ choice of a healthcare facility type is invariably influenced by their perceived quality-of-care [13,14]. In Kenya, a survey reported that perceived quality in terms of perceived staff competence and shorter waiting times significantly influenced preferences for private clinics over public hospitals [15]. Also, Olorunfemi et al. [16] in Nigeria reveal that patients often chose faith-based hospitals due to perceived quality-of-care in terms of better interpersonal treatment, despite higher costs. Similarly, Atinga et al. [17] in Ghana found that perceived healthcare quality pushes patients toward private providers. This implies that, irrespective of it being publicly or privately financed, the quality of healthcare services impacts patients’ choices for facility type. Studies also reveal that accessibility factors impact patients’ choice of a healthcare facility type. For instance, Verma and Dash [18] found that 68% of respondents preferred local clinics over distant hospitals due to travel constraints. Syed et al. [19] reported that urban populations favored specialized facilities, whereas rural residents relied on primary care centers due to shorter travel distances. Unaffordable costs, low income levels, and out-of-pocket expenses influence the use of public facilities over private facilities [20,21]. Poor road networks [22,23], travel time and distance [24] were also found to influence the choice of healthcare among household members. Otchere et al. [25] on accessibility and utilization of health-care services among rural–urban migrants in Ghana, revealed that rural Ghanaians prioritized proximity, with 60% opting for Community-Based Health Planning. These pieces of evidence demonstrate that geographical accessibility, financial barriers, and infrastructural limitations influence patients’ choices for healthcare facility type.

Despite the efforts of existing studies [18–25] highlighting factors that impact individual choice of healthcare facility type, very few have disaggregated analysis at the level of PHC facility type, leaving unclear why patients distinguish between and selectively prefer specific categories of PHC providers within the same geographic and policy environment. Also, the overwhelming majority of facility preference studies have been conducted in urban settings [7,26], rendering their findings poorly transferable to rural and semi-urban municipal contexts such as Bolgatanga, where informal health norms, community trust structures, and provider-patient relational dynamics operate differently. Again, with the need to promote the utilization of PHC services in Bolgatanga Municipality due to a recent report on inconsistent utilization patterns across different PHC facilities, there is the need to assess the existing factors that impact patients’ choices of PHC facility type for local context evidence to support national decision-making. To achieve this aim, the following research questions were addressed.

1. What is the role of healthcare service quality in shaping individual preferences for healthcare facility type in Bolgatanga Municipality in the Upper East Region of Ghana?
2. What is the effect of accessibility factors on individual preferences for healthcare facility type in Bolgatanga Municipality in the Upper East Region of Ghana?

## Methods and materials

### Study design and population

The study employed a cross-sectional study design. A cross-sectional design is appropriate for this study because it enabled the simultaneous collection of data on quality-of-care, accessibility factors, and healthcare facility preferences from a defined population at a single point in time, making it both time- and cost-efficient for examining associations across a large sample in the Bolgatanga Municipality [27]. This study included adults 18 years and above who live in Bolgatanga and accepted the consent to participate. This study excluded individuals younger than 18 years and adults who are seriously ill or mentally unfit to participate in the research and patients who did not give consent.

### Profile of study area

This study was confined to the settings of Bolgatanga. Bolgatanga Municipal is in the center of the Upper East Region and is the regional capital. It has a total land area of 729 sq. km and is bordered to the north by the Bongo District, to the south and east by Talensi, and to the west by the Kassena-Nankana Municipality. The municipality was established by LI 1797 (2004) [28]. As of 2021, the town had a population of about 142,509 people, with females (74,659) representing 52.4% and males (67,850) representing 47.6%. The municipality is divided into nine (9) sub-municipalities, namely Bolga Central, Bolga North, Bolga South, Kalbeo, Sherigu, Sumbrungu East, Sumbrungu West, Plaza and Zaare. The capital of the municipality is Bolgatanga, which is cosmopolitan. The major tribe is Frafra, with significant tribes from within the Upper East Region, as well as other neighbouring regions in the country. The climate is classified as tropical and has two distinct seasons, the rainy season that runs from May to October and a long dry season that stretches from October to April with hardly any rain. Mean annual rainfall is 950 mm, while the maximum temperature is 45°C in March and April with a minimum of 12°C in December [28]. The selection of this study area was influenced by inconsistent utilization patterns across different PHC facilities in the municipality observed by the health workers.

### Theoretical model/Framework

This study employed Andersen’s Behavioral Model of Health Services Use as the theoretical framework to explain the key factors that influence individual preferences for PHC facility type in Bolgatanga Municipality, Upper East Region, Ghana.

In the context of this study, the model’s enabling and predisposing domains directly inform both the research design and the selection of variables. Specifically, the accessibility factors examined, namely distance to facility, transportation options, and cost of service, are theoretically situated within Andersen’s enabling domain, as they constitute the structural and economic conditions that either facilitate or impede an individual’s capacity to reach and engage with a preferred PHC facility type [29,31]. Concurrently, the healthcare service quality dimensions encompassing waiting time, cleanliness, communication with provider, and drug availability align predominantly with Andersen’s predisposing and enabling constructs, as they reflect individuals’ evaluative perceptions of the care environment that shape attitudinal dispositions and reinforce or redirect facility preferences over repeated health-seeking episodes. The model’s conceptual logic further justifies the adoption of a cross-sectional research design, as Andersen’s framework is fundamentally a snapshot model of utilization behavior. It theorizes the simultaneous interplay of predisposing, enabling, and need conditions at a given point in time rather than tracing longitudinal behavioral trajectories, making a cross-sectional survey the most epistemologically coherent design for capturing and measuring the concurrent influence of these variable domains on facility-type preference. The PHC facility types included in the study are District hospital, Private clinic/hospital/Faith based, Government health centers or polyclinic and CHPS compound. District hospitals were included because, within the Ghana Health Service referral framework, they often serve as the first point of contact for many communities and provide a substantial proportion of primary healthcare services, particularly in areas like Bolgatanga, where lower-level facilities have limited capacity. Respondents were asked about their preferred facility for seeking primary healthcare services rather than hypothetical choices. Analytically, the model’s tripartite structure guides the organization of variables into theoretically meaningful blocks, informing a multivariate analytical approach, most appropriately multinomial logistic regression, wherein accessibility factors and service quality dimensions are examined as independent predictors of facility-type preference, consistent with Andersen’s proposition that utilization outcomes are the product of the additive and interactive influence of these determinant domains. Consequently, far from being a passive theoretical reference, Andersen’s Behavioral Model of Health Services actively structures every methodological decision in this study, from what is measured and why to how variables are categorized and the manner in which their relationships with PHC facility-type preferences are estimated and interpreted, thereby ensuring that the study’s empirical inquiry is theoretically grounded, internally coherent, and meaningfully positioned within the broader canon of health services utilization research [29,30].

### Sampling methods

The optimal sample size was determined using the Yamane (1973) formula which assumes simple random sampling [32], with population (N = 142,509), and margin of error (e = 0.05). A total of 398 accurate and complete questionnaires were obtained, which matched the actual sample size estimated without the nonresponse rate. For this study, the researcher used a three-stage cluster sampling method. The absence of design effect adjustment is due to the fact that the primary objective of the study was to obtain population-level estimates and examine associations among variables rather than produce highly precise cluster-level estimates. Though the use of random selection at each sampling stage helped to reduce selection bias, the use of multistage cluster sampling may have introduced intra-cluster correlation, which could result in larger standard errors and reduced statistical precision.

In the first stage, the study area was divided into clusters based on the nine (9) sub-municipalities, namely Bolga Central, Bolga North, Bolga South, Kalbeo, Sherigu, Sumbrungu East, Sumbrungu West, Plaza, and Zaare. From these, 5 clusters (Bolga Central, Bolga South, Sherigu, Sumbrungu East, and Plaza) were randomly selected. In the second stage, the researchers randomly selected compounds from each selected cluster by assigning codes with the aid of random number generator software. The researcher then listed all the households in each selected compound. A household here means a father, mother, and children or relatives under one roof, so a compound may have more than one household. Sub-municipalities may differ substantially in socioeconomic status, education, ethnicity, and urbanization, and households located in compounds of different sizes may have unequal probabilities of selection. To address this, the number of compounds selected per sub-municipality was proportional to the total number of compounds identified in that cluster, thereby minimizing selection bias arising from compound size heterogeneity. From the 250 selected compound households, the researcher selected not more than two adults from each household at random to take part in the study at the third stage. Here, and with the aid of random number generator software, the participants were assigned a code and surveyed. This method made the process fair and ensured that every household has an equal chance of being included.

### Data collection techniques

Data for this study was collected using researcher-administered questionnaires. A novel questionnaire was developed by the researchers specifically for the purposes of this study and uploaded under the supplementary file. These were administered through face-to-face interviews, which helped to ensure accurate responses from participants who had difficulties reading or understanding the questions independently. The questionnaire developed for this study underwent a rigorous content validity process, whereby five experts in health research from the department of Health Policy, Management, and Economics, KNUST independently evaluated each item for relevance, clarity, and representativeness using a Content Validity Index (CVI), yielding an item-level CVI (I-CVI) of ≥0.80 and a scale-level CVI (S-CVI) of 0.92, consistent with established thresholds for acceptable content validity. To further establish instrument reliability, a pilot test was conducted with 20 respondents drawn from the Tafo community, Kumasi. The pilot exercise focused on instrument refinement rather than context-specific behavioural measurement; therefore, emphasis was placed on linguistic clarity and interpretability of items rather than contextual equivalence of responses. However, we recognize that Kumasi is socio-culturally and geographically different from the study area (Bolgatanga), and this may limit the extent to which contextual nuances were captured during pretesting. Also, internal consistency was measured using a Cronbach’s alpha, where all constructs yielded a coefficient ≥ 0.70. This confirms satisfactory internal consistency of the self-reported items. Construct validity was additionally supported via expert panel feedback, which was used iteratively to refine item wording for interpretability and contextual appropriateness prior to final administration.

### Data analysis

The data was entered and analyzed using Stata Version 17.0 and SPSS Version 22.0. SPSS Version 22.0 was used for descriptive statistics and multinomial logistic regression, while Stata Version 17 was used for additional model diagnostics, including VIF estimation. Descriptive data were presented using tables, frequencies, percentages, and charts where necessary. Initial bivariate analysis was conducted to examine crude associations between healthcare facility preference and all independent variables, including healthcare service and accessibility factors. Specifically, chi-square tests were used for categorical variables to identify statistically significant relationships at the bivariate level (p < 0.05), consistent with standard modeling procedures in health services research. Variables that demonstrated theoretical relevance or significant crude associations were subsequently considered for inclusion in the multivariable model to control for potential confounding effects and improve the robustness of parameter estimates. Multinomial logistic regression was also used to determine the role of healthcare service quality and accessibility factors in shaping individual preferences for healthcare facility type. The model diagnostic tests revealed a Cox & Snell Pseudo R² value of 0.341 for perceived-quality-of-care and 0.431 for accessibility factors, indicating that the independent variables collectively explain approximately 34.1% and 43.1% of the variation in PHC facility-type preference and the two models, respectively. The Variance Inflation Factor (VIF) values ranging from 1.198 to 1.523 and corresponding tolerance values between 0.657 and 0.835 for all the models are all well within acceptable thresholds, confirming the absence of multicollinearity among the predictor variables, thereby affirming the statistical integrity of the regression estimates. Statistical significance for all testing was set at 0.05.

### Ethics approval

Ethical approval for this study was obtained from the Committee on Human Research, Publications, and Ethics (CHRPE), KNUST, in relation to the Ghana Data Protection Act, 2012 (Act 843) national guidelines, with the reference number CHRPE/AP/011/26. The study was conducted in accordance with the ethical principles outlined in the Declaration of Helsinki. All participants were informed about the purpose of the study, assured of confidentiality, and provided written informed consent prior to participation. All participants were subject to freely given, informed consent to participate in the study. Participation was voluntary, and respondents could withdraw at any time without consequence.

## Results

### Socio-demographic characteristics of the respondents

Table 1 provides a summary of the socio-demographic characteristics of the 398 respondents. The results show that the largest age group was 31–45 years (43.0%), while the smallest was 61–83 years (6.3%). Also, most of the respondents had completed senior high school (32.2%), whereas the least had no formal education (18.8%). In terms of marital status, the majority of the respondents were married (53.8%), and only a small proportion were cohabiting (1.0%). Christianity was the dominant religion (59.5%), while those with no religion formed the smallest group (2.0%). By occupation, traders constituted the largest category (25.9%), whereas those in the other category (retired, security guard, and hairdresser) accounted for the smallest share (3.8%). Income distribution showed that most respondents earned GHC 200–1500 (41.0%), whereas only 4.0% earned GHC 5001–7000. Finally, NHIS status revealed that most respondents were registered and active members (74.9%), while the least were not registered (3.8%). The respondents had a mean age of 39.5 years with standard deviation of 12.3.

**Table 1:** Socio-demographic characteristics of the respondents.

Variable		Frequency (N = 398)	Percentage (%)
Age	18–30yrs	139	34.9
	31–45yrs	171	43.0
	46–60yrs	63	15.8
	61–83yrs	25	6.3
Highest level of education	No formal education	75	18.8
	Basic (Up to JHS)	83	20.9
	Senior High School (SHS)	128	32.2
	Tertiary (Diploma, HND, Degree)	112	28.1
Marital status	Single	155	38.9
	Married	214	53.8
	Cohabiting	4	1.0
	Divorced/Separated	13	3.3
	Widowed	12	3.0
Religion	Christianity	237	59.5
	Islam	103	25.9
	Traditional Religion	50	12.6
	No religion	8	2.0
Occupation	Unemployed	83	20.9
	Farmer	45	11.3
	Trader	103	25.9
	Civil/Public servant	75	18.8
	Private sector worker	22	5.5
	Artisan (e.g., carpenter, mason)	22	5.5
	Student	33	8.3
	Other	15	3.8
Income	GHC200–1500	163	41.0
	GHC1501–3000	83	20.9
	GHC3001–5000	136	34.2
	GHC5001–7000	16	4.0
NHIS Status	Not Registered	15	3.8
	Registered but not active	85	21.4
	Registered and active membership	298	74.9

### The role of healthcare service quality in shaping individual preferences for PHC facility type

Table 2 presents a descriptive analysis of the quality-of-care, preferred healthcare facility type, and factors influencing respondents’ healthcare choices. Among the 398 respondents, most rated the quality-of-care as good (45.7%), whereas very few rated it as poor (0.5%) or excellent (1.0%). For service use, the majority preferred facilities for routine check-ups (62.8%), whereas the smallest proportion visited for specialized treatment (9.3%). District hospitals were the most preferred PHC facilities (43.2%), while private clinics, faith-based facilities, and CHPS compounds were the least preferred (12.8% each).

**Table 2:** Descriptive analysis of quality-of-care and preferred PHCfacility type.

Variable			Frequency(f)	Percentage (%)
Quality-of-care	Poor		2	0.5
	Good		182	45.7
	Fair		128	32.2
	Very Good		82	20.6
	Excellent		4	1.0
Healthcare facility preferred for	Routine check-ups		250	62.8
	Emergency care		111	27.9
	Specialized treatment		37	9.3
Preferred PHC facility types	District hospital		172	43.2
	Private clinic/hospital/Faith based		51	12.8
	Government health centers or polyclinic		124	31.2
	CHPS compound		51	12.8
**TOTAL (N)**			**398**	**100%**

Table 3 presents the descriptive analysis of factors influencing individual preferences for healthcare facility type, with most respondents consistently rated quality-of-care (76.4%), waiting time (77.1%), staff attitude (75.1%), availability of follow-up care (70.1%), specialized services (68.8%), cost (57.0%), medicine availability (67.3%), and clean environment (70.9%) as important, with very few considering these factors not important. Among influence-related variables, personal recommendation (77.4%), facility reputation (74.6%), insurance coverage (75.9%), and accessibility (76.1%) were rated as highly influential, whereas online reviews were rated moderately influential (59.0%), making them the least influential factor overall.

**Table 3:** Descriptive analysis of factors influencing individual preferences for healthcare facility type.

Factor	Not Important	Neutral	Important	Very Important
Quality-of-care	0 (0.0%)	13 (3.3%)	304 (76.4%)	81 (20.4%)
Waiting time	0 (0.0%)	9 (2.3%)	306 (77.1%)	82 (20.7%)
Staff attitude & communication	0 (0.0%)	17 (4.3%)	299 (75.1%)	82 (20.6%)
Availability of follow-up care	19 (4.8%)	27 (6.8%)	279 (70.1%)	73 (18.3%)
Availability of specialized services	13 (3.3%)	33 (8.3%)	274 (68.8%)	78 (19.6%)
Cost	0 (0.0%)	23 (5.8%)	225 (57.0%)	147 (37.2%)
Availability of medicines	0 (0.0%)	12 (3.0%)	268 (67.3%)	118 (29.6%)
Clean environment	2 (0.5%)	22 (5.5%)	282 (70.9%)	92 (23.1%)
	**Not Influential**	**Neutral**	**Influential**	**Very Influential**
Personal recommendations	4 (1.0%)	36 (9.0%)	308 (77.4%)	50 (12.6%)
Online reviews	12 (3.0%)	107 (26.9%)	235 (59.0%)	44 (11.1%)
Facility reputation	6 (1.5%)	39 (9.8%)	297 (74.6%)	56 (14.1%)
Insurance coverage	0 (0.0%)	14 (3.5%)	302 (75.9%)	82 (20.6%)
Accessibility and location	0 (0.0%)	16(4.1%)	300(76.1%)	78(19.8%)
		**N = 398(100%)**		

Table 4 shows that quality-of-care, age, education, NHIS status, and cost were significantly associated with respondents’ preferred PHC facility type (p < 0.05). Quality-of-care demonstrated a strong association with facility preference (χ² = 18.927, p = 0.004), suggesting that respondents who considered quality-of-care important or very important were more likely to prefer higher-level health facilities. Age was also significantly associated with facility preference (χ² = 21.468, p = 0.011). Educational attainment significantly influenced facility choice (χ² = 19.374, p = 0.022) and NHIS status was significantly associated with preferred facility type (χ² = 12.861, p = 0.045). Additionally, perceived cost significantly affected facility preference (χ² = 15.286, p = 0.018), indicating that financial considerations remain an important determinant of PHC facility selection.

**Table 4:** Bivariate association between quality-of-care and preferred PHC facility type.

Variable	Category	District hospital n (%)	Private clinic/Faith-based n (%)	Govt health centre/Polyclinic n (%)	CHPS compound n (%)	χ²	p-value
Quality-of-care	Not Important	5 (38.5)	1 (7.7)	3 (23.1)	4 (30.8)	18.927	0.004
	Important	132 (43.4)	44 (14.5)	89 (29.3)	39 (12.8)		
	Very Important	35 (43.2)	6 (7.4)	32 (39.5)	8 (9.9)		
Age	18–30 yrs	63 (45.3)	21 (15.1)	40 (28.8)	15 (10.8)	21.468	0.011
	31–45 yrs	74 (43.3)	19 (11.1)	60 (35.1)	18 (10.5)		
	46–60 yrs	27 (42.9)	6 (9.5)	14 (22.2)	16 (25.4)		
	61–83 yrs	8 (32.0)	5 (20.0)	10 (40.0)	2 (8.0)		
Education	No formal education	35 (46.7)	7 (9.3)	21 (28.0)	12 (16.0)	19.374	0.022
	Basic	32 (38.6)	13 (15.7)	27 (32.5)	11 (13.3)		
	SHS	59 (46.1)	13 (10.2)	45 (35.2)	11 (8.6)		
	Tertiary	46 (41.1)	18 (16.1)	31 (27.7)	17 (15.2)		
NHIS status	Not registered	7 (46.7)	2 (13.3)	3 (20.0)	3 (20.0)	12.861	0.045
	Registered, not active	38 (44.7)	15 (17.6)	22 (25.9)	10 (11.8)		
	Registered, active	127 (42.6)	34 (11.4)	99 (33.2)	38 (12.8)		
Cost	Not Important	11 (47.8)	3 (13.0)	8 (34.8)	1 (4.3)	15.286	0.018
	Important	101 (44.3)	30 (13.2)	70 (30.7)	27 (11.8)		
	Very Important	60 (40.8)	18 (12.2)	46 (31.3)	23 (15.6)		

*NB: Age, educational level, NHIS status and cost were included in the chi-square analysis because they were treated as covariates (controlled variables) in the multivariable logistic regression model.*

Tables 5 present a multinomial logistic regression result examining the association between quality-of-care and the type of PHC facility preferred. The model was statistically significant (χ²(36) = 208.71, p < .001), indicating that the predictors jointly explained a meaningful proportion of variance in facility choice. The multinomial logistic regression results indicate that quality-of-care significantly influences respondents’ preferences for PHC type after adjusting for age, education, NHIS status, and cost. For private clinics/faith-based facilities, respondents who rated quality-of-care as very important compared to not important are 74% more likely to prefer for private clinics/faith-based facilities compared to CHPS compounds (aOR = 1.74, 95% CI: 1.02–9.73, p = 0.026). Among those who considered quality-of-care important rather than not important were 56% higher (aOR = 1.56, 95% CI: 1.05–2.91, p = 0.008). Both respondents who rated quality-of-care as important (aOR = 2.89, 95% CI: 1.02–9.78, p = 0.033) and very important (aOR = 1.25, 95% CI: 1.01–3.61, p = 0.004) had higher odds of preferring district hospitals over CHPS compounds.

**Table 5:** Multinomial logistic regression of the association between quality-of-care and preferred PHC facility type.

Variable		Odds (95% CI)	p-value
**Private clinic / Faith-based vs CHPS**			
Quality-of-care	Not Important	**Ref**	
	Important	0.95 (0.240, 5.68)	0.994
	Very Important	1.74 (1.02, 9.73)	0.026
Age	18–30 yrs	Ref	
	31–45 yrs	3.27 (1.17, 9.08)	0.023
	46–60 yrs	2.87 (0.51, 16.05)	0.23
	61–83 yrs	2.69 (0.02, 14.73)	0.991
Education	No formal education	**Ref**	
	Basic	0.90 (0.25, 3.29)	0.872
	SHS	2.41 (0.69, 8.45)	0.166
	Tertiary	4.83 (2.90, 9.67)	0.027
NHIS status	Not registered	**Ref**	
	Registered, not active	3.05 (0.34, 27.70)	0.322
	Registered, active	0.76.07 (0.13, 0.97)	0.003
Cost	Not Important	Ref	
	Important	0.68 (0.09, 5.36)	0.723
	Very Important	0.17 (0.01, 0.73)	0.029
**Government health centre / Polyclinic vs CHPS**			
Quality-of-care	Not Important	**Ref**	
	Important	1.56 (1.05, 2.91)	0.008
	Very Important	0.46 (0.13, 0.89)	0.992
Age	18–30 yrs	Ref	
	31–45 yrs	3.83 (1.64, 8.95)	0.002
	46–60 yrs	3.33 (0.88, 12.60)	0.077
	61–83 yrs	3.02 (0.21, 11.58)	0.992
Education	No formal education	**Ref**	
	Basic	0.61 (0.22, 1.69)	0.345
	SHS	1.64 (0.60, 4.49)	0.334
	Tertiary	3.76 (0.88, 16.02)	0.074
NHIS status	Not registered	**Ref**	
	Registered, not active	1.90 (0.29, 10.41)	0.505
	Registered, active	3.26 (0.57, 16.96)	0.183
Cost	Neutral	**Ref**	
	Important	5.45 (1.55, 10.83)	0.025
	Very Important	3.02 (0.76, 29.65)	0.341
**District hospital vs CHPS**			
Quality-of-care	Not Important	**Ref**	
	Important	2.89(1.02, 9.78)	0.033
	Very Important	1.25 (1.01, 3.61)	0.004
Age	18–30 yrs	Ref	
	31–45 yrs	8.87 (3.63, 21.62)	< 0.001
	46–60 yrs	14.60 (3.79, 26.07)	< 0.001
	61–83 yrs	14.71 (0.00, 61.09)	0.991
Education	No formal education	**Ref**	
	Basic	1.21 (0.40, 4.37)	0.733
	SHS	3.03 (1.01, 9.09)	0.047
	Tertiary	14.08 (3.19, 62.02)	< 0.001
NHIS status	Not registered	**Ref**	
	Registered, not active	2.96 (0.45, 19.56)	0.259
	Registered, active	2.53 (0.45, 14.19)	0.295
Cost	Not Important	**Ref**	
	Important	1.33 (0.07, 9.63)	0.779
	Very Important	0.61 (0.09, 0.98)	0.023

NB: Ref  =  Reference category.

### Effect of accessibility factors on individual preferences for healthcare facility type

Table 6 provides a descriptive analysis of accessibility factors and barriers to healthcare services among the respondents. Among the 398 respondents, the majority had used healthcare services in the past six months (68.1%), whereas a smaller proportion had not (31.9%). Among the 271 individuals who had accessed care, the majority relied on both public and private facilities (57.6%), with fewer depending solely on public or private facilities (10.7%). Location played a major role in healthcare decisions, with most respondents (74.9%) indicating that it was influential, while only a small fraction found it not influential (1.0%). Willingness to travel varied by service type: most respondents were willing to travel further for specialized care (82.4%) and emergency care (60.8%), but willingness dropped for routine care, where slightly more than half (51.3%) were unwilling. Regarding barriers, most respondents reported no barriers (69.3%). Among the 122 who experienced challenges, cost emerged as the most common barrier (80.3%), followed by long waiting times (57.4%) and distance (32%).

**Table 6:** Descriptive analysis of accessibility factors and barriers to accessing healthcare services.

Item		Frequency (f)	Percentage (%)
Have you used healthcare services in the past 6 months?	Yes	271	68.1
	No	127	31.9
**TOTAL**		**398**	**100%**
If yes, what type of healthcare facility do you typically use?			
	Public	86	31.7
	Private	29	10.7
	Both private and pubic	156	57.6
**TOTAL**		**271**	**100%**
How does the location of a healthcare facility influence your decision to use it?	Not Influential at All	25	6.3
	Not Influential	4	1.0
	Neutral	41	10.3
	Influential	298	74.9
	Very Influential	30	7.5
Would you be willing to travel further for Specialized care	No	70	17.6
	Yes	328	82.4
Would you be willing to travel further for Emergency care	No	156	39.2
	Yes	242	60.8
Would you be willing to travel further for Routine care	No	204	51.3
	Yes	194	48.7
Have you experienced any barriers to accessing healthcare services?	Yes	122	30.7
	No	276	69.3
**TOTAL**		**398**	**100%**
If yes what were the barriers (Multiple responses) transportation issues	No	112	91.8
	Yes	10	8.2
Long waiting times	No	52	42.6
	Yes	70	57.4
Cost	No	24	19.7
	Yes	98	80.3
Distance	No	83	68.0
	Yes	39	32.
Other (No drugs, community conflict)	No	118	96.7
	Yes	4	3.3
**TOTAL**		**610**	**100%**

NB: *Totals may exceed the sample size because respondents could select more than one option.

Table 7 indicates that all accessibility-related factors were significantly associated with respondents’ preferred PHC facility type (p < 0.05). Distance and location showed a significant association with facility preference (χ² = 18.963, p = 0.004). Transportation options exhibited one of the strongest associations (χ² = 22.618, p = 0.001), highlighting the importance of transport availability in determining preferred facilities. Waiting time was also significantly associated with facility preference (χ² = 20.483, p = 0.002). Appointment availability significantly influenced respondents’ choices (χ² = 17.536, p = 0.008). The cost of transportation was significantly associated with preferred PHC facility type (χ² = 16.112, p = 0.013), suggesting that travel-related costs remain an important consideration in healthcare-seeking behaviour.

**Table 7:** Bivariate association between accessibility factors and preferred PHC facility type.

Variable	Category	District hospital n (%)	Private clinic/Faith-based n (%)	Govt health centre/Polyclinic n (%)	CHPS compound n (%)	χ²	p-value
Distance & Location	Not Important	10 (50.0)	3 (15.0)	7 (35.0)	0 (0.0)	18.963	0.004
	Important	115 (39.7)	37 (12.8)	95 (32.8)	43 (14.8)		
	Very Important	47 (53.4)	11 (12.5)	22 (25.0)	8 (9.1)		
Transportation Options	Not Important	12 (48.0)	6 (24.0)	2 (8.0)	5 (20.0)	22.618	0.001
	Important	125 (43.9)	30 (10.5)	95 (33.3)	35 (12.3)		
	Very Important	35 (39.8)	15 (17.0)	27 (30.7)	11 (12.5)		
Waiting Time	Not Important	5 (33.3)	3 (20.0)	7 (46.7)	0 (0.0)	20.483	0.002
	Important	129 (43.7)	41 (13.9)	87 (29.5)	38 (12.9)		
	Very Important	38 (43.2)	7 (8.0)	30 (34.1)	13 (14.8)		
Appointment Availability	Not Important	15 (50.0)	6 (20.0)	7 (23.3)	2 (6.7)	17.536	0.008
	Important	117 (41.1)	34 (11.9)	97 (34.0)	37 (13.0)		
	Very Important	40 (48.2)	11 (13.3)	20 (24.1)	12 (14.5)		
Cost of transportation	Not Important	11 (55.0)	1 (5.0)	5 (25.0)	3 (15.0)	16.112	0.013
	Important	101 (43.9)	32 (13.9)	72 (31.3)	25 (10.9)		
	Very Important	60 (40.5)	18 (12.2)	47 (31.8)	23 (15.5)		

Tables 8 and 9 present the results of a multinomial logistic regression examining the influence of accessibility factors on the type of PHC facility preferred. The model was statistically significant: χ² (43) = 109.66, p < .001 (indicating that distance, transportation options, waiting time, appointment availability, and cost jointly explained a meaningful proportion of variance in facility choice). Specifically, respondents who rated distance and location as very important rather than not important were 242% more likely to choose private clinics/faith-based facilities rather than CHPS compounds (aOR = 3.42, 95% CI: 1.78–6.84, p = 0.001). Those who considered transportation options as very important compared to not important were 301% more likely to select private clinics/faith-based facilities rather than CHPS compounds (aOR = 4.01, 95% CI: 2.10–7.83, p < 0.001). Respondents who rated waiting time as very important compared to not important are 21% more likely to choose private clinics/faith-based facilities compared to CHPS compounds (aOR = 1.21, 95% CI: 1.10–2.47, p < 0.001), while those who considered appointment availability as very important are 131% more likely to prefer these facilities compared to CHPS compounds (aOR = 2.31, 95% CI: 1.14–5.61, p = 0.001). Conversely, respondents who rated cost of care as very important are 12% less likely to choose private clinics/faith-based facilities compared to CHPS compounds (aOR = 0.88, 95% CI: 0.02–0.95, p < 0.001).

**Table 8:** Multinomial logistic regression of accessibility factors influencing preferred PHC facility type.

Variable	Odds (95% CI)	P-value	
**Private clinic / Faith-based vs CHPS**			
Distance & Location	Not Important	**Ref**	
	Important	1.88 (1.10, 3.57)	0.042
	Very Important	3.42 (1.78, 6.84)	0.001
Transportation Options	Not Important	Ref	
	Important	2.15 (1.24, 4.02)	0.009
	Very Important	4.01 (2.10, 7.83)	< 0.001
Waiting Time	Not Important	**Ref**	
	Important	1.54 (1.09, 2.97)	0.021
	Very Important	1.21 (1.10, 2.47)	< 0.001
Appointment Availability	Not Important	**Ref**	
	Important	1.48 (0.26, 0.91)	0.024
	Very Important	2.31 (1.14, 5.61)	0.001
Cost of care	Not Important	Ref	
	Important	0.74 (0.11, 0.99)	0.028
	Very Important	0.88 (0.02, 0.95)	< 0.001
**Government Health Centre / Polyclinic vs CHPS**			
Distance & Location	Not Important	**Ref**	
	Important	1.72 (1.04, 3.14)	0.049
	Very Important	2.34 (1.24, 4.56)	0.009
Transportation Options	Not Important	**Ref**	
	Important	2.28 (1.29, 4.26)	0.006
	Very Important	3.48 (1.82, 6.68)	< 0.001
Waiting Time	Not Important	Ref	
	Important	0.47 (0.26, 0.82)	0.008
	Very Important	0.29 (0.14, 0.55)	0.001
Appointment Availability	Not Important	**Ref**	
	Important	0.49 (0.27, 0.91)	0.024
	Very Important	0.34 (0.17, 0.66)	0.002
Cost of Transportation	Not Important	**Ref**	
	Important	1.62 (0.95, 3.21)	0.071
	Very Important	2.71 (1.40, 5.37)	0.003

*NB: Ref = Reference category*.

**Table 9:** Multinomial logistic regression of accessibility factors influencing preferred PHC facility type cont’d.

Variable		Odds (95% CI)	P-value
**District Hospital vs CHPS**			
Distance & Location	Not Important	**Ref**	
	Important	0.35 (0.27, 0.79)	0.008
	Very Important	4.55 (2029, 8.91)	0.510
Transportation Options	Not Important	**Ref**	
	Important	0.68 (0.02, 0.95)	0.002
	Very Important	5.98 (0.94, 11.85)	0.700
Waiting Time	Not Important	**Ref**	
	Important	0.39 (0.21, 0.70)	0.002
	Very Important	0.17 (0.08, 0.33)	< 0.001
Appointment Availability	Not Important	**Ref**	
	Important	0.50 (0.27, 0.94)	0.032
	Very Important	0.32 (0.16, 0.62)	0.001
Cost of care	Not Important	**Ref**	
	Important	2.20 (1.13, 4.28)	0.020
	Very Important	4.18 (2.11, 8.21)	< 0.001

*NB: Ref = Reference category*.

For government health centres or polyclinics, respondents who rated distance and location as very important are 134% more likely to select these facilities compared to CHPS compounds (aOR = 2.34, 95% CI: 1.24–4.56, p = 0.009), and those who considered transportation options as very important are 248% more likely to prefer them (aOR = 3.48, 95% CI: 1.82–6.68, p < 0.001). In contrast, respondents who rated waiting time as very important are 71% less likely to choose government facilities (aOR = 0.29, 95% CI: 0.14–0.55, p = 0.001), and those who considered appointment availability as very important are 66% less likely to select these facilities (aOR = 0.34, 95% CI: 0.17–0.66, p = 0.002). Respondents who rated the cost of transportation as very important are 171% more likely to prefer government health centres or polyclinics (aOR = 2.71, 95% CI: 1.40–5.37, p = 0.003). Regarding district hospitals, respondents who rated distance and location as very important are 355% more likely to choose district hospitals compared to CHPS compounds (aOR = 4.55, 95% CI: 2.09–8.91, p = 0.510), and those who considered transportation options as very important are 498% more likely to select them (aOR = 5.98, 95% CI: 0.94–11.85, p = 0.700). Conversely, respondents who rated waiting time as very important are 83% less likely to choose district hospitals (aOR = 0.17, 95% CI: 0.08–0.33, p < 0.001), while those who considered appointment availability as very important are 68% less likely to select these facilities (aOR = 0.32, 95% CI: 0.16–0.62, p = 0.001). Respondents who rated cost of care as very important are 318% more likely to prefer district hospitals (aOR = 4.18, 95% CI: 2.11–8.21, p < 0.001).

## Discussions

“Perceived quality-of-care emerged as a determinant of individuals’ preference for PHC facility type in the Bolgatanga Municipality. In specific contexts, private or faith-based facilities, government health centers or polyclinics, and district hospitals rather than CHPS compounds were preferred by individuals who attributed greater importance to quality-of-care. This shows that higher-level or alternative facilities that are perceived to offer better services are opted for due to higher valuation of quality. Also, individuals assess the standard of care they anticipate receiving and justify their facility choice with perceptions of competence, responsiveness, and service reliability beyond mere physical access. This shows a growing demand for healthcare services that are not only accessible but also trustworthy, patient-centered, and effective. Through the assumptions of Andersen’s Behavioral Model of Health Services Use, the findings reflect the interaction between predisposing, enabling, and need factors influencing an individual’s preference for healthcare facility type [31]. Here, the perceived quality-of-care operates as 1) a predisposing factor, through individual beliefs and expectations about healthcare. 2) a need-related factor, as patients seek facilities they believe can adequately address their health problems [29,30]. This shows that the choice of hospitals and private facilities among those who highly value quality-of-care suggests that when enabling resources are available, perceived quality becomes a decisive driver of utilization.”

“The current finding is supported by Bahrampour et al. [14] who report that service quality in terms of low waiting time, staff attitude, cleanliness, and follow-up care influenced patients’ hospital preferences in Iran. In the same way, Gabrani et al. [33] supported that both public and private healthcare users in Albania prioritized quality-of-care when choosing facilities. Gage et al. [34] showed that perceptions of provider responsiveness and service quality shaped utilization of PHC facilities in Haiti. Studies in Africa by Mwami and Oleche [35] and Lokina and Musili [15] in supporting this current finding reported that perceived staff competence, shorter waiting times, and better interpersonal treatment drove patients toward private clinics in Kenya. Olorunfemi et al. [16] in a similar context observed similar preferences for faith-based facilities in Nigeria due to better provider–patient interactions. On the other hand, Mhlanga and Hassan [36] found that cost considerations outweighed perceived quality among low-income households in South Africa. And this leads to continued preference for public facilities despite dissatisfaction. Also, the current findings showed that while the highest education level (Odds = 3.76, p = 0.074) was not significantly related to the choice of government health centre, NHIS status was also not significantly associated with both government health centers (Odds = 3.26) and district hospitals (Odds = 2.53). While these can be explained by structural and supply-side barriers such as facility availability, perceived quality, and proximity that may override individual-level socioeconomic characteristics in driving healthcare-seeking decisions in resource-constrained settings like Bolgatanga, the methodological limitations of this study are of concern. Methodologically, the cross-sectional design precludes any causal inference, as both exposure and outcome are measured simultaneously, making it impossible to determine whether education or NHIS status precedes or follows facility preference. The use of multinomial logistic regression, while appropriate for polytomous outcomes, is sensitive to small cell sizes across facility categories, which may have inflated standard errors and reduced statistical power. This is a particularly acute concern, as the analysis is geographically confined to the Bolgatanga municipality alone, limiting both sample diversity and the generalizability of findings to other urban, peri-urban, or rural contexts in the Upper East Region or Ghana broadly. Despite the demerits, these findings suggest the need to strengthen quality-of-care at the primary level, particularly within CHPS compounds. Future studies should adopt larger, multi-site probability samples across diverse districts, incorporate longitudinal designs to track how NHIS enrollment dynamically influences facility choice over time, and consider multilevel modeling to disentangle individual-level from facility-level determinants.

“In Bolgatanga, accessibility-related factors such as distance and location, transportation options, waiting time, appointment availability, and cost considerations were found to relate to individuals’ preferences for PHC facility type. This shows that multiple accessibility factors interact in individuals’ daily lives in selecting facility type and that healthcare choices are not driven by a single access. From the study, individuals who valued proximity and transportation convenience preferred private clinics, faith-based facilities, and district hospitals. This may suggest that patients are willing to bypass nearby CHPS compounds when other facilities are perceived to be easier to reach or better connected by transport. Interestingly, dissatisfaction with congestion and delays commonly associated with higher-level public facilities were revealed in the study. Where participants who valued shorter waiting times and easier appointment availability were less likely to choose hospitals and government facilities. Also, the persistent relationship between financial barriers and an individual’s healthcare behavior, particularly in low-resource settings, was revealed, where respondents who prioritized cost showed a lower preference for private facilities. In the context of this study, Andersen’s Behavioral Model of Health Services Use, the findings relate to the central role of enabling factors in healthcare utilization. Thus, distance, transportation options, waiting time, appointment systems, and cost represent key enabling or constraining conditions that determine whether individuals can translate their healthcare needs into actual service use [29,30].”

Studies in India and the United States support the current findings by showing that distance, travel time, and transportation barriers significantly relate to facility choice, with rural populations often preferring nearby primary care facilities due to mobility constraints [18,19]. Guimarães et al. [20] and Akkazieva and Zhao [21] also support the current findings, where they reported that financial accessibility often overrides geographical proximity, as low-income households avoid facilities perceived as costly even when they are close. Studies in Africa such as Mangundu et al. [22] and Oriyomi [23] support the current findings, documenting how long distances, poor road networks, and transport costs push rural households toward closer or informal healthcare options. In Ghana, Asante [24] and Otchere et al. [25] showed that proximity and travel time encourage the use of CHPS compounds, which support the current findings. Asare-Akuffo et al. [37] in supporting the current findings, found that individuals may travel longer distances when they perceive higher-level facilities as more capable of addressing their health needs. The identification of proximity and transportation convenience as drivers of preference for private clinics, faith-based facilities, and district hospitals particularly adds nuance to demand-side analyses in low-resource urban settings. Nonetheless, the study’s cross-sectional, single-municipality design represents a significant methodological limitation. It captures a static snapshot of preferences without accounting for seasonal variation in access, such as during rainy seasons when transportation is disrupted, and the geographically bounded sample constrains transferability of findings beyond Bolgatanga’s specific infrastructural and socioeconomic context. Furthermore, reliance on self-reported preferences may introduce social desirability bias, and the absence of objective facility-level data such as actual travel distances and verified waiting times limits triangulation of subjective perceptions against measurable access indicators. Despite the limitations, the study suggests the need for integrated interventions that address both physical and organizational access to care. That is, enhancing road networks, strengthening public transport systems, and decentralizing services could reduce the burden of distance and transportation barriers. Future research should employ mixed-methods longitudinal designs across multiple municipalities, integrating Geographic Information Systems (GIS) mapping of facility catchment areas with qualitative exploration of how accessibility trade-offs are negotiated across different household types and seasons.

## Conclusions

The study aimed to identify the factors associated with individuals’ preferences for PHC facility type in the Bolgatanga Municipality, Upper East Region, Ghana. We found that quality-of-care and accessibility-related factors such as distance and location, transportation options, waiting time, appointment availability, and cost considerations were associated with individuals’ preferences for PHC facility type. Therefore, the study suggests that healthcare managers in Bolgatanga and the Ministry of Health should prioritize interventions aimed at improving quality-of-care, such as improving interpersonal communication between health workers and patients, enhancing staff training, ensuring consistent availability of medications, and maintaining a hygienic and safe facility environment across all facility types. They should also provide effective and reliable transportation options, optimizing the location of new facilities, reducing waiting times through appointment management systems, and ensuring reasonable service fees. By addressing these quality-of-care gaps, public and CHPS facilities may become more attractive to residents who currently prefer private or faith-based facilities due to perceived superior service. Also, with these measures, barriers that currently drive patients toward higher-level facilities while maintaining affordability for low-income populations would reduce.

For methodological enhancement, future studies should adopt larger, multi-site probability samples across diverse districts, incorporate longitudinal designs to track how NHIS enrollment dynamically influences facility choice over time, and consider multilevel modeling to disentangle individual-level from facility-level determinants. Also, future research should employ mixed-methods longitudinal designs across multiple municipalities, integrating Geographic Information Systems (GIS) mapping of facility catchment areas with qualitative exploration of how accessibility trade-offs are negotiated across different household types and seasons. Also, linking survey data with routine health information systems would provide stronger behavioural validation.

## Supporting information

S1 Data Data collection tool PHC: Data collection instrument designed for the purpose of this study. (DOCX)

S2 Data PHC dataset. (CSV)
