# Supplementary material for: Factors associated with individuals’ preferences for Primary Health Care facility type: A case of Bolgatanga Municipality in the Upper East Region of Ghana
Source: PLoS One. 2026 Aug 20;21(8):e0354800. doi: 10.1371/journal.pone.0354800 (PMC13492761; doi:10.1371/journal.pone.0354800)
Supplement: Supplementary file 2 [file pone.0354800.s005.pdf]

Charles Obeng,
Department of Health Policy, Management and Economics,
Kwame Nkrumah University of Science and Technology (KNUST)
Kumasi, Ghana.
Email: obengcharles436@gmail.com
12 June 2026

The Editor-in-Chief
PLOS ONE

Re: Response to Reviewers – Revised Manuscript

Dear Editor,

We sincerely thank the Editor and all three Reviewers for their constructive and detailed feedback. We have carefully addressed each comment in the revised manuscript. Below, we provide point-by-point responses. Reviewer comments are presented in italics; our responses follow in regular text.

AUDITOR'S COMMENT

Comment:
Delete all subheadings from the Discussion section to ensure smoother flow.

Response:
All subheadings have been removed from the Discussion section. The content has been restructured into continuous prose to improve readability and flow.

REVIEWER #1

Comment:
Justify the selection of only quality of care, age, education, NHIS status, and cost in the multinomial logistic regression.

Response:
We appreciate this comment. Quality of care was the primary explanatory variable, directly aligned with the study's objective of examining its influence on facility preference. Age, education, NHIS status, and cost were included as control variables to adjust for potential confounding effects. These covariates were selected based on theoretical relevance and evidence from prior literature demonstrating their consistent influence on healthcare-seeking behaviour and facility utilisation decisions.

REVIEWER #2

<u>General Comments</u>

Comment 1: Conceptualization of Facility Preference

District hospitals are included as PHC facilities but often function as secondary-level referral facilities in Ghana. The authors should justify their inclusion and clarify whether questions concerned actual utilization or hypothetical preference, and how facility categories align with Ghana Health Service (GHS) classifications.

Response:

We acknowledge this concern. In the Ghanaian health system context, district hospitals serve dual roles, often functioning as the primary point of care in districts with limited health centre coverage. For this study, PHC facility types were defined based on the functional and accessible role they play in the community, consistent with how GHS operationalises first-contact care at the district level. Additionally, the questionnaire focused on respondents' preferences for healthcare facility use rather than referral utilization. This clarification has been added to the Methods section (page 7, lines 195–201).

Comment 2: Questionnaire Validation

No information is provided on the parameters of expert review (reliability, construct validity, consistency, or interpretability) for the novel questionnaire.

Response:

We have strengthened the reporting of instrument validation. The revised manuscript now includes details on the expert review process, including the criteria assessed (content relevance, clarity, interpretability, and comprehensiveness) and the feedback integration process. A pilot test was also conducted to assess internal consistency. These details have been added to the relevant section of the Methods (page 8, lines 234–244).

Comment 3: Informatics Perspective

Reliance on self-reported perceptions limits validation of actual healthcare-seeking behaviour. Integration of routine health information system data would strengthen the research.

Response:

We acknowledge this observation. This study deliberately relied on primary self-reported data because the objective was to capture individuals' perceived preferences and behavioural intentions, which are not fully observable in routine health records. Self-reported survey data are appropriate for understanding subjective decision-making, particularly where perceptions, accessibility, and socio-cultural factors drive behaviour. The integration of routine health information system data has been highlighted as a direction for future research in the revised manuscript.

Comment 4: Sample Size Calculation

The manuscript references Yamane's formula but does not report the population size or margin of error used.

Response:

The population size and margin of error (5%) used in Yamane's formula have now been explicitly reported in the revised Methods section (page 7, lines 214–215).

Comment 5: Overall Grammar and Style

Numerous grammatical and stylistic issues require editing, including inconsistent capitalization of healthcare facility names, inconsistent use of percentages and decimals, and inconsistent use of PHC as a concept versus system.

Response:

The manuscript has been thoroughly revised for grammar and style. Specifically: (1) 'Primary Healthcare' has been harmonized throughout; (2) PHC is now consistently used as a level of healthcare delivery; (3) abbreviations are written out at first use (including WHO at line 46); (4) reference formatting has been made consistent throughout; (5) line 62 and 71–72 have been revised for clarity; and (6) a professional language review has been undertaken. All flagged instances have been corrected in the revised manuscript.

REVIEWER #3

1. Statistical and Methodological Issues

Comment 1: Missing Bivariate Analysis

The title references 'Factors Associated with...' but no bivariate analysis (e.g., chi-square tests) was conducted prior to multivariable logistic regression.

Response:

We agree with this comment. Bivariate analyses (chi-square tests) were conducted to examine crude associations between predictor variables and facility preference prior to multivariable modelling. These results have now been incorporated into the Methods section and the relevant results table to justify predictor selection for the final model (page 8, lines 243–250).

Comment 2: Model Separation (Impossible Odds Ratios)

The Odds Ratio for the '61–83 yrs' age category is reported as 6.41×10^7 with missing confidence intervals, indicating complete or quasi-complete separation.

Response:

We thank the reviewer for this observation. The issue arose from sparse data in the 61–83 years age category. The correct figures have been provided and the tables updated accordingly.

Comment 3: Pilot Study Location Mismatch

The questionnaire was piloted in Kumasi, a highly urbanised setting, whereas the main study was conducted in Bolgatanga, a rural/semi-urban northern setting.

Response:

We acknowledge this limitation. The pilot was conducted in Kumasi primarily for logistical reasons and due to the availability of a comparable population of healthcare users. While we recognise the socio-cultural and geographic differences, the pilot was intended to assess instrument clarity, response consistency, and administration feasibility rather than contextual representativeness. This has been acknowledged revised manuscript. (Page 8, line 231-236)

Comment 4: Software Ambiguity

Line 228 references both Stata Version 17 and SPSS Version 22, but results appear to come from SPSS alone. Clarify which software was used for which analysis.

Response:

The Methods section has been revised to clearly specify that SPSS Version 22 was used for descriptive statistics and multinomial logistic regression, while Stata Version 17 was used for additional model diagnostics including VIF estimation. This distinction is now explicitly stated. Line 242-244

Comment 5: Model Diagnostics Clarification

Clarify to which specific models the reported VIF and pseudo-R^2 values apply.

Response:

The revised manuscript now explicitly states that the VIF values apply to the explanatory variables in the multinomial logistic regression model used to examine factors associated with facility preference, and that the pseudo-R^2 (Nagelkerke) pertains to the same model. These clarifications have been added to the Methods section (page 8).

2. Structural and Formatting Issues

Comment 6: Introduction Too Verbose

The introduction is too verbose; the theoretical model framework section should be reduced.

Response:

The introduction has been substantially tightened. Redundant background information has been removed and the theoretical framework section reduced to its essential elements, consistent with standard scientific writing conventions.

Comment 7: Methodology Repetition

The study design and population sections contain redundant repetitions.

Response:

Repetitive content in the study design and population sections has been consolidated (page 5).

Comment 8: Irrelevant Population Profile Details

Remove extraneous details (e.g., basketry, tomato farming) unless explicitly linked to occupational health-seeking behavior or income in the analysis.

Response:

Extraneous population profile details have been removed from the revised manuscript (page 5).

Comment 9: Table Restructuring (Tables 3 & 4)

Split Table 2 into two tables: one for descriptive analysis of Quality of Care and PHC Facility Type, and another for Factors Influencing Individual Preferences. Renumber subsequent tables accordingly.

Response:

Table 2 has been split into two distinct tables as recommended. The first focuses on descriptive analysis of Quality of Care and Preferred PHC Facility Type, and the second presents factors influencing individual preferences. The subsequent tables have been renumbered accordingly.

Comment 10: Conclusion Too Long

The conclusion contains redundant material that belongs in the Discussion section.

Response:

The conclusion has been revised and condensed to present only the main takeaways and implications of the study. Material more appropriate for the Discussion section has been relocated accordingly (page 21).

Comment 11: Reference Formatting

Verify consistency of all references; Line 407 incorrectly uses an author name instead of the required numbered citation format.

Response:

All references have been reviewed and formatted consistently. The error on Line 407 (Lederle et al.) has been corrected to the appropriate numbered citation format.

3. Specific Line-by-Line Edits

Comment 12: Line 19

Change 'the study' to 'this study'.

Response:

Corrected.

Comment 13: Line 141

Change 'and denied their consent' to 'patients who did not give consent'.

Response:

Corrected.

Comment 14: Line 164 – Figure Numbering

Text references 'Figure 2' but there is no Figure 1. Correct figure numbering sequentially.

Response:

Figure numbering has been corrected and is now sequential throughout the manuscript.

Comment 15: Line 228

Change 'Data were' to 'Data was' for consistency.

Response:

Corrected.

Comment 16: P-values in Tables 3, 4, 6, 7

A p-value of '0.000' is statistically impossible. All instances should read $p < 0.001$.

Response:

All instances of '0.000' in the tables have been changed to '$p < 0.001$'.

Comment 17: Table 3 Typo

Fix typographical error in Odds Ratio for the Cost variable, currently written as '0.76.07'.

Response:
The typographical error in Table 3 has been corrected.

We trust that the revisions adequately address all the concerns raised. We are grateful for the opportunity to improve the manuscript and hope it is now suitable for publication.

Sincerely,

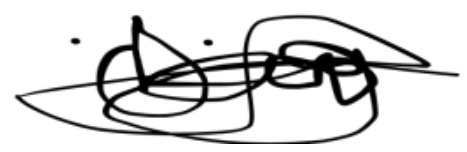

Mr. Charles Obeng (Corresponding Author 1)
Cc: Mrs. Sussana Atanga (Corresponding Author 2)
Prof. Kofi Akohene Mensah (PhD)
Mr. Lawal Alhassan
Tell: +233 (0) 550889312
