# Supplementary material for: Factors associated with individuals’ preferences for Primary Health Care facility type: A case of Bolgatanga Municipality in the Upper East Region of Ghana
Source: PLoS One. 2026 Aug 20;21(8):e0354800. doi: 10.1371/journal.pone.0354800 (PMC13492761; doi:10.1371/journal.pone.0354800)
Supplement: Supplementary file 3 [file pone.0354800.s006.pdf]

PLOS One

Factors Associated with Individuals' Preferences for Primary Healthcare Facility Type in Bolgatanga Municipality in the Upper East Region of Ghana.

--Manuscript Draft--

**Manuscript Number:**	PONE-D-26-23521R1
**Article Type:**	Research Article
**Full Title:**	Factors Associated with Individuals' Preferences for Primary Healthcare Facility Type in Bolgatanga Municipality in the Upper East Region of Ghana.
**Short Title:**	Factors Associated with Individuals' Preferences for Primary Healthcare Facility Type
**Corresponding Author:**	Charles Obeng, Mphil<br>Kwame Nkrumah University of Science and Technology College of Health Sciences<br>Kumasi, GHANA
**Keywords:**	quality of care; Primary Healthcare Facility Type; Distance and Location; Transportation Options; waiting time; Appointment Availability.
**Abstract:**	Background: There is a recognizable gap regarding the specific determinants that drive preferences for different types of Primary Health Care (PHC) facilities, despite the efforts of prior scholars on factors influencing patients' preferences for facility type. To address this gap, the study identified the key factors influencing individuals' preferences for PHC facility type in the Bolgatanga Municipality, Upper East Region, Ghana.<br>Methods: This study employed a two-stage cluster sampling method, and 398 residents in the Bolgatanga Municipality of the Upper East Region were surveyed with structured questionnaires acting as the primary data collection tool. Data was entered and analyzed using Stata Version 17.0 and SPSS Version 22.0 computer software. Frequencies, percentages, and multinomial logistic regression statistical analysis were employed in analyzing the filed data.<br>Results: Quality-of-care (aOR = 2.89, 95% CI: 1.02–9.78, p = 0.033) was a significant determinant of individuals' preference for PHC facility type. The study also found that accessibility-related factors such as distance and location (aOR = 3.42, 95% CI: 1.78–6.84, p = 0.001), transportation options (aOR = 4.01, 95% CI: 2.10–7.83, p < 0.001), waiting time (aOR = 1.21, 95% CI: 1.10–2.47, p < 0.001), appointment availability (aOR = 2.31, 95% CI: 1.14–5.61, p = 0.001) and cost of care (aOR = 0.88, 95% CI: 0.02–0.95, p < 0.001) are associated with individuals' preferences for PHC facility type.<br>Conclusion: The study established perceived quality-of-care and accessibility factors significantly influenced individual preferences for PHC facility type, with cost-sensitive and nearby Community-Based Health Planning and Services (CHPS) compounds remaining crucial for clients with limited resources. Therefore, the management of facilities within Bolgatanga Municipality and similar contexts should improve service quality, reduce waiting times, and enhance transport and financial accessibility to ensure equitable utilization of PHC services."
**Order of Authors:**	Charles Obeng, Mphil
	Sussana Atanga, MSc
	Kofi Akohene Mensah, PhD
	Lawal Alhassan, BSc
**Response to Reviewers:**	AUDITOR'S COMMENT<br>Comment:<br>Delete all subheadings from the Discussion section to ensure smoother flow.<br>Response:<br>All subheadings have been removed from the Discussion section. The content has been restructured into continuous prose to improve readability and flow.<br>REVIEWER #1<br>Comment:<br>Justify the selection of only quality of care, age, education, NHIS status, and cost in the multinomial logistic regression.

Powered by Editorial Manager® and ProduXion Manager® from Aries Systems Corporation

Response:
We appreciate this comment. Quality of care was the primary explanatory variable, directly aligned with the study's objective of examining its influence on facility preference. Age, education, NHIS status, and cost were included as control variables to adjust for potential confounding effects. These covariates were selected based on theoretical relevance and evidence from prior literature demonstrating their consistent influence on healthcare-seeking behaviour and facility utilisation decisions.
REVIEWER #2
General Comments
Comment 1: Conceptualization of Facility Preference
District hospitals are included as PHC facilities but often function as secondary-level referral facilities in Ghana. The authors should justify their inclusion and clarify whether questions concerned actual utilization or hypothetical preference, and how facility categories align with Ghana Health Service (GHS) classifications.
Response:
We acknowledge this concern. In the Ghanaian health system context, district hospitals serve dual roles, often functioning as the primary point of care in districts with limited health centre coverage. For this study, PHC facility types were defined based on the functional and accessible role they play in the community, consistent with how GHS operationalises first-contact care at the district level. Additionally, the questionnaire focused on respondents' preferences for healthcare facility use rather than referral utilization. This clarification has been added to the Methods section (page 7, lines 195–201).
Comment 2: Questionnaire Validation
No information is provided on the parameters of expert review (reliability, construct validity, consistency, or interpretability) for the novel questionnaire.
Response:
We have strengthened the reporting of instrument validation. The revised manuscript now includes details on the expert review process, including the criteria assessed (content relevance, clarity, interpretability, and comprehensiveness) and the feedback integration process. A pilot test was also conducted to assess internal consistency. These details have been added to the relevant section of the Methods (page 8, lines 234–244).
Comment 3: Informatics Perspective
Reliance on self-reported perceptions limits validation of actual healthcare-seeking behaviour. Integration of routine health information system data would strengthen the research.
Response:
We acknowledge this observation. This study deliberately relied on primary self-reported data because the objective was to capture individuals' perceived preferences and behavioural intentions, which are not fully observable in routine health records. Self-reported survey data are appropriate for understanding subjective decision-making, particularly where perceptions, accessibility, and socio-cultural factors drive behaviour. The integration of routine health information system data has been highlighted as a direction for future research in the revised manuscript.
Comment 4: Sample Size Calculation
The manuscript references Yamane's formula but does not report the population size or margin of error used.
Response:
The population size and margin of error (5%) used in Yamane's formula have now been explicitly reported in the revised Methods section (page 7, lines 214–215).
Comment 5: Overall Grammar and Style
Numerous grammatical and stylistic issues require editing, including inconsistent capitalization of healthcare facility names, inconsistent use of percentages and decimals, and inconsistent use of PHC as a concept versus system.
Response:
The manuscript has been thoroughly revised for grammar and style. Specifically: (1) 'Primary Healthcare' has been harmonized throughout; (2) PHC is now consistently used as a level of healthcare delivery; (3) abbreviations are written out at first use (including WHO at line 46); (4) reference formatting has been made consistent throughout; (5) line 62 and 71–72 have been revised for clarity; and (6) a professional language review has been undertaken. All flagged instances have been corrected in the revised manuscript.
REVIEWER #3

	1. Statistical and Methodological Issues<br>Comment 1: Missing Bivariate Analysis<br>The title references 'Factors Associated with...' but no bivariate analysis (e.g., chi-square tests) was conducted prior to multivariable logistic regression.<br>Response:<br>We agree with this comment. Bivariate analyses (chi-square tests) were conducted to examine crude associations between predictor variables and facility preference prior to multivariable modelling. These results have now been incorporated into the Methods section and the relevant results table to justify predictor selection for the final model (page 8, lines 243–250).<br>Comment 2: Model Separation (Impossible Odds Ratios)<br>The Odds Ratio for the '61–83 yrs' age category is reported as 6.41 × 10^7 with missing confidence intervals, indicating complete or quasi-complete separation.<br>Response:<br>We thank the reviewer for this observation. The issue arose from sparse data in the 61–83 years age category. The correct figures have been provided and the tables updated accordingly.<br>Comment 3: Pilot Study Location Mismatch<br>The questionnaire was piloted in Kumasi, a highly urbanised setting, whereas the main study was conducted in Bolgatanga, a rural/semi-urban northern setting.<br>Response:<br>We acknowledge this limitation. The pilot was conducted in Kumasi primarily for logistical reasons and due to the availability of a comparable population of healthcare users. While we recognise the socio-cultural and geographic differences, the pilot was intended to assess instrument clarity, response consistency, and administration feasibility rather than contextual representativeness. This has been acknowledged revised manuscript. (Page 8, line 231-236)<br>Comment 4: Software Ambiguity<br>Line 228 references both Stata Version 17 and SPSS Version 22, but results appear to come from SPSS alone. Clarify which software was used for which analysis.<br>Response:<br>The Methods section has been revised to clearly specify that SPSS Version 22 was used for descriptive statistics and multinomial logistic regression, while Stata Version 17 was used for additional model diagnostics including VIF estimation. This distinction is now explicitly stated. Line 242-244<br>Comment 5: Model Diagnostics Clarification<br>Clarify to which specific models the reported VIF and pseudo-R^2 values apply.<br>Response:<br>The revised manuscript now explicitly states that the VIF values apply to the explanatory variables in the multinomial logistic regression model used to examine factors associated with facility preference, and that the pseudo-R^2 (Nagelkerke) pertains to the same model. These clarifications have been added to the Methods section (page 8).<br>2. Structural and Formatting Issues<br>Comment 6: Introduction Too Verbose<br>The introduction is too verbose; the theoretical model framework section should be reduced.<br>Response:<br>The introduction has been substantially tightened. Redundant background information has been removed and the theoretical framework section reduced to its essential elements, consistent with standard scientific writing conventions.<br>Comment 7: Methodology Repetition<br>The study design and population sections contain redundant repetitions.<br>Response:<br>Repetitive content in the study design and population sections has been consolidated (page 5).<br>Comment 8: Irrelevant Population Profile Details<br>Remove extraneous details (e.g., basketry, tomato farming) unless explicitly linked to occupational health-seeking behavior or income in the analysis.<br>Response:<br>Extraneous population profile details have been removed from the revised manuscript (page 5).<br>Comment 9: Table Restructuring (Tables 3 & 4)<br>Split Table 2 into two tables: one for descriptive analysis of Quality of Care and PHC

Powered by Editorial Manager® and ProduXion Manager® from Aries Systems Corporation

	Facility Type, and another for Factors Influencing Individual Preferences. Renumber subsequent tables accordingly.<br>Response:<br>Table 2 has been split into two distinct tables as recommended. The first focuses on descriptive analysis of Quality of Care and Preferred PHC Facility Type, and the second presents factors influencing individual preferences. The subsequent tables have been renumbered accordingly.<br>Comment 10: Conclusion Too Long<br>The conclusion contains redundant material that belongs in the Discussion section.<br>Response:<br>The conclusion has been revised and condensed to present only the main takeaways and implications of the study. Material more appropriate for the Discussion section has been relocated accordingly (page 21).<br>Comment 11: Reference Formatting<br>Verify consistency of all references; Line 407 incorrectly uses an author name instead of the required numbered citation format.<br>Response:<br>All references have been reviewed and formatted consistently. The error on Line 407 (Lederle et al.) has been corrected to the appropriate numbered citation format.<br>3. Specific Line-by-Line Edits<br>Comment 12: Line 19<br>Change 'the study' to 'this study'.<br>Response:<br>Corrected.<br>Comment 13: Line 141<br>Change 'and denied their consent' to 'patients who did not give consent'.<br>Response:<br>Corrected.<br>Comment 14: Line 164 – Figure Numbering<br>Text references 'Figure 2' but there is no Figure 1. Correct figure numbering sequentially.<br>Response:<br>Figure numbering has been corrected and is now sequential throughout the manuscript.<br>Comment 15: Line 228<br>Change 'Data were' to 'Data was' for consistency.<br>Response:<br>Corrected.<br>Comment 16: P-values in Tables 3, 4, 6, 7<br>A p-value of '0.000' is statistically impossible. All instances should read p < 0.001.<br>Response:<br>All instances of '0.000' in the tables have been changed to 'p < 0.001'.<br>Comment 17: Table 3 Typo<br>Fix typographical error in Odds Ratio for the Cost variable, currently written as '0.76.07'.<br>Response:<br>The typographical error in Table 3 has been corrected.
**Additional Information:**	
**Question**	**Response**
**Competing Interests**<br><br>Use the instructions below to enter a competing interest statement for this submission. On behalf of all authors, disclose any competing interests that could be perceived to bias this work—acknowledging all financial support and any other relevant financial or non-financial competing interests.	The authors have declared that no competing interests exist.

This statement is required for submission and **will appear in the published article** if the submission is accepted. Please make sure it is accurate and that any funding sources listed in your Funding Information later in the submission form are also declared in your Financial Disclosure statement.<br><br>View published research articles from *PLOS ONE* for specific examples.<br><br>**NO authors have competing interests**<br><br>Enter: *The authors have declared that no competing interests exist.*<br><br>**Authors with competing interests**<br><br>Enter competing interest details beginning with this statement:<br><br>*I have read the journal's policy and the authors of this manuscript have the following competing interests: [insert competing interests here]*<br><br>* typeset	
**Financial Disclosure**<br><br>Enter a financial disclosure statement that describes the sources of funding for the work included in this submission. Review the submission guidelines for detailed requirements. View published research articles from *PLOS ONE* for specific examples.<br><br>This statement is required for submission and **will appear in the published article** if the submission is accepted. Please make sure it is accurate.	The author(s) received no specific funding for this work.

**Funded studies**<br>Enter a statement with the following details:<br>• Initials of the authors who received each award<br>• Grant numbers awarded to each author<br>• The full name of each funder<br>• URL of each funder website<br>• Did the sponsors or funders play any role in the study design, data collection and analysis, decision to publish, or preparation of the manuscript?<br><br>Did you receive funding for this work?	
**Ethics Statement**<br><br>Enter an ethics statement for this submission. This statement is required if the study involved:<br><br>• Human participants<br>• Human specimens or tissue<br>• Vertebrate animals or cephalopods<br>• Vertebrate embryos or tissues<br>• Field research<br><br>Write "N/A" if the submission does not require an ethics statement.<br><br>General guidance is provided below. Consult the submission guidelines for detailed instructions. **Make sure that all information entered here is included in the Methods section of the manuscript.**	Ethical approval for this study was obtained from the Committee on Human Research, Publications, and Ethics (CHRPE), KNUST, in relation to the Ghana Data Protection Act, 2012 (Act 843) national guidelines, with the reference number CHRPE/AP/011/26. The study was conducted in accordance with the ethical principles outlined in the Declaration of Helsinki. All participants were informed about the purpose of the study, assured of confidentiality, and provided written informed consent prior to participation. All participants were subject to freely given, informed consent to participate in the study. Participation was voluntary, and respondents could withdraw at any time without consequence.

Powered by Editorial Manager® and ProduXion Manager® from Aries Systems Corporation

**Format for specific study types**<br><br>**Human Subject Research (involving human participants and/or tissue)**<br>• Give the name of the institutional review board or ethics committee that approved the study<br>• Include the approval number and/or a statement indicating approval of this research<br>• Indicate the form of consent obtained (written/oral) or the reason that consent was not obtained (e.g. the data were analyzed anonymously)<br><br>**Animal Research (involving vertebrate animals, embryos or tissues)**<br>• Provide the name of the Institutional Animal Care and Use Committee (IACUC) or other relevant ethics board that reviewed the study protocol, and indicate whether they approved this research or granted a formal waiver of ethical approval<br>• Include an approval number if one was obtained<br>• If the study involved *non-human primates*, add *additional details* about animal welfare and steps taken to ameliorate suffering<br>• If anesthesia, euthanasia, or any kind of animal sacrifice is part of the study, include briefly which substances and/or methods were applied<br><br>**Field Research**<br><br>Include the following details if this study involves the collection of plant, animal, or other materials from a natural setting:<br>• Field permit number<br>• Name of the institution or relevant body that granted permission	
**Data Availability**<br><br>Before publication, Authors are required to make fully available and without restriction all data underlying their findings. Sharing the data underlying your findings increases the visibility, credibility, and impact of your work, while supporting transparency and future scientific discovery. Please see our PLOS Data	The datasets generated and/or analysed during the current study have been uploaded here in excel format, with the file name "Supplementary File_1_PHC_Dataset."

Powered by Editorial Manager® and ProduXion Manager® from Aries Systems Corporation

Policy page for detailed information on this policy.<br><br>A **Data Availability Statement**, detailing where the data can be accessed, is required at first submission. Insert your Data Availability Statement in the box below.<br><br>Please see the data reporting section of our submission guidelines for instructions on what you need to include in your Data Availability Statement. Examples of acceptable Data Availability Statements are included here for reference:<br>1. The minimal data set [and accompanying code, where generated] is available at [repository name] via [DOI hyperlink]<br>2. No data was generated by this study. The following existing data sources were used: [dataset name] from [dataset location] available via [web address or DOI hyperlink].<br>3. For Study Protocols: No datasets were generated or analysed during the current study. All relevant data from this study will be made available upon study completion.<br><br>*PLOS allows rare exemptions to address legal and ethical concerns. If you have legal or ethical restrictions, please use the box below to detail these in full sentences for the Journal team to consider.*<br><br>This statement will be typeset if the manuscript is accepted for publication.	

Powered by Editorial Manager® and ProduXion Manager® from Aries Systems Corporation

Manuscript Click here to access/download;Manuscript;Manuscript_15_06_2026.docx

Factors Associated with Individuals' Preferences for Primary Health Care Facility Type in Bolgatanga Municipality in the Upper East Region of Ghana.

Charles Obeng [1]*, Sussana Atanga [2]*, Kofi Akohene Mensah[3], Lawal Alhassan[4]

[1]* Department of Health Policy, Management, and Economics, Kwame Nkrumah University of Science and Technology (KNUST), Kumasi, Ghana

[2]* Bolgatanga Municipal Health Directorate, Reproductive and Child Health Unit, Bolga, Ghana.

[3]Department of Health Policy, Management and Economics, Kwame Nkrumah University of Science and Technology (KNUST), Kumasi, Ghana

[4]Bolgatanga Municipal Health Directorate, Office of Municipal Director of Health Service, Bolga, Ghana.

Corresponding Authors

[1]* Email: obengcharles436@gmail.com
[2]* Email: atangasussan85@gmail.com

Abstract

Background: There is a recognizable gap regarding the specific determinants that drive preferences for different types of Primary Health Care (PHC) facilities, despite the efforts of prior scholars on factors influencing patients' preferences for facility type. To address this gap, the study identified the key factors influencing individuals' preferences for PHC facility type in the Bolgatanga Municipality, Upper East Region, Ghana.

Methods: This study employed a two-stage cluster sampling method, and 398 residents in the Bolgatanga Municipality of the Upper East Region were surveyed with structured questionnaires acting as the primary data collection tool. Data was entered and analyzed using Stata Version 17.0 and SPSS Version 22.0 computer software. Frequencies, percentages, and multinomial logistic regression statistical analysis were employed in analyzing the filed data.

Results: Quality-of-care (aOR = 2.89, 95% CI: 1.02–9.78, p = 0.033) was a significant determinant of individuals' preference for PHC facility type. The study also found that accessibility-related factors such as distance and location (aOR = 3.42, 95% CI: 1.78–6.84, p = 0.001), transportation options (aOR = 4.01, 95% CI: 2.10–7.83, p < 0.001), waiting time (aOR = 1.21, 95% CI: 1.10–2.47, p < 0.001), appointment availability (aOR = 2.31, 95% CI:

1.14–5.61, p = 0.001) and cost of care (aOR = 0.88, 95% CI: 0.02–0.95, $p < 0.001$) are associated with individuals' preferences for PHC facility type.

Conclusion: The study established perceived quality-of-care and accessibility factors significantly influenced individual preferences for PHC facility type, with cost-sensitive and nearby Community-Based Health Planning and Services (CHPS) compounds remaining crucial for clients with limited resources. Therefore, the management of facilities within Bolgatanga Municipality and similar contexts should improve service quality, reduce waiting times, and enhance transport and financial accessibility to ensure equitable utilization of PHC services.

Keywords: Quality-of-care; Primary Health Care Facility Type; Distance and Location; Transportation Options; Waiting Time; Appointment Availability.

Introduction

Globally, Primary Health Care (PHC) has been a crucial component of the healthcare system in many countries. This level of healthcare delivery functions as the first point of contact between patients and the health system, and plays a significant role in ensuring access to essential health services [1]. According to the World Health Organization (WHO) [2], Primary Health Care (PHC) is a whole-of-society approach to health that aims to ensure the highest possible level of health and well-being and their equitable distribution by focusing on population's needs as early as possible along the continuum from health promotion and disease prevention to treatment, rehabilitation, and palliative care, as close as feasible to people's everyday environment. PHC facilities consist of a different array of settings, such as community health centers, clinics, health posts, and general practitioner practices, which function as the frontline of healthcare delivery [3]. Global declarations, such as the Alma-Ata Declaration of 1978 and the Astana Declaration of 2018, unequivocally underscore the international commitment to PHC as the most equitable, effective, and efficient path to health [2].

PHC is the most inclusive, equitable, cost-effective, and efficient approach to enhancing people's physical and mental health and social well-being. For instance, WHO [2] asserted that scaling up primary health care (PHC) interventions across low and middle-income countries could save 60 million lives and improve average life expectancy by 3.7 years by 2030. An estimated 75% of the projected health gains from the Sustainable Development Goals could be achieved through PHC [2, 4] . In Low-Middle-Income Countries (LMICs) like Rwanda, community health worker programs and health posts have optimized the transformative power

of PHC that has significantly improved access to basic care and health outcomes in rural areas [5]. Also, Tanzania through the Health Basket Fund channeled to the PHC facilities has focused on strengthening its primary care infrastructure and improving service delivery at the district level, recognizing PHC as key to decentralized health governance and equitable access [6]. Similarly, in Ghana, the Community-Based Health Planning and Services (CHPS) compounds, representing the first contact of the health system, are designed to bring preventive, promotive, and basic curative services directly to users and communities, especially in rural areas, thereby improving access to immunizations, maternal and child health services, and common disease management [7]. Ghana has in previous years explicitly adopted PHC principles aimed at improving health at the basic level through its National Health Insurance Scheme (NHIS). This NHIS aims to reduce financial barriers to care, particularly at the primary care level. [8]. This approach aligns with PHC's emphasis on community participation and ownership, fostering local engagement in health planning and delivery. These regional efforts demonstrate how PHC enhances health system resilience, enabling better preparedness and response to public health emergencies, as evidenced by adaptations made during the COVID-19 pandemic across the continent [9].

PHC delivery effectiveness is dependent on the efficient use of diverse facility types. And this functions as the bedrock for universal health coverage and improved health outcomes of the population [7, 10]. However, patients do not consistently utilize the nearest or most appropriate PHC facility despite the investments by member states to expand PHC infrastructure and services, particularly in LMICs like Ghana. Rather, their healthcare-seeking behavior reflects deliberate preferences for specific clinic or hospital types, often bypassing proximate facilities in favor of more distant ones [7, 10]. In Ghana, the number of outpatient consultations in PHC has not increased significantly, and patients still seek medical care at secondary or tertiary hospitals rather than PHC institutions as their first point of contact, leading to excessive demand and a burden on secondary and tertiary hospitals, while underutilizing PHC institutions [11]. For instance, In the Upper West Region of Ghana, a study by Farhan and Aryeetey [12] found a 77.8% utilization rate of secondary and tertiary healthcare compared to 22.2% utilization rate for PHC, suggesting a considerable wastage of high-cost medical resources in the secondary and tertiary hospitals. This can easily result in prolonged waiting time for patients and excessive workloads for doctors and other healthcare providers. Research asserts that this preference or health-seeking behavior is influenced by an intricate interplay of various factors [7].

Studies demonstrate that patients' choice of a healthcare facility type is invariably influenced by their perceived quality-of-care [13, 14]. In Kenya, a survey reported that perceived quality in terms of perceived staff competence and shorter waiting times significantly influenced preferences for private clinics over public hospitals [15]. Also, Olorunfemi et al. [16] in Nigeria reveal that patients often chose faith-based hospitals due to perceived quality-of-care in terms of better interpersonal treatment, despite higher costs. Similarly, Atinga et al. [17] in Ghana found that perceived healthcare quality pushes patients toward private providers. This implies that, irrespective of it being publicly or privately financed, the quality of healthcare services impacts patients' choices for facility type. Studies also reveal that accessibility factors impact patients' choice of a healthcare facility type. For instance, Verma and Dash [18] found that 68% of respondents preferred local clinics over distant hospitals due to travel constraints. Syed et al. [19] reported that urban populations favored specialized facilities, whereas rural residents relied on primary care centers due to shorter travel distances. Unaffordable costs, low income levels, and out-of-pocket expenses influence the use of public facilities over private facilities [20, 21]. Poor road networks [22, 23], travel time and distance [24] were also found to influence the choice of healthcare among household members. Otchere et al. [25] on accessibility and utilization of health-care services among rural–urban migrants in Ghana, revealed that rural Ghanaians prioritized proximity, with 60% opting for Community-Based Health Planning. These pieces of evidence demonstrate that geographical accessibility, financial barriers, and infrastructural limitations influence patients' choices for healthcare facility type.

Despite the efforts of existing studies [18–25] highlighting factors that impact individual choice of healthcare facility type, very few have disaggregated analysis at the level of PHC facility type, leaving unclear why patients distinguish between and selectively prefer specific categories of PHC providers within the same geographic and policy environment. Also, the overwhelming majority of facility preference studies have been conducted in urban settings [7, 26], rendering their findings poorly transferable to rural and semi-urban municipal contexts such as Bolgatanga, where informal health norms, community trust structures, and provider-patient relational dynamics operate differently. Again, with the need to promote the utilization of PHC services in Bolgatanga Municipality due to a recent report on inconsistent utilization patterns across different PHC facilities, there is the need to assess the existing factors that impact patients' choices of PHC facility type for local context evidence to support national decision-making. To achieve this aim, the following research questions were addressed.

1. What is the role of healthcare service quality in shaping individual preferences for healthcare facility type in Bolgatanga Municipality in the Upper East Region of Ghana?
2. What is the effect of accessibility factors on individual preferences for healthcare facility type in Bolgatanga Municipality in the Upper East Region of Ghana?

Methods and Materials

Study design and population

The study employed a cross-sectional study design. A cross-sectional design is appropriate for this study because it enabled the simultaneous collection of data on quality-of-care, accessibility factors, and healthcare facility preferences from a defined population at a single point in time, making it both time- and cost-efficient for examining associations across a large sample in the Bolgatanga Municipality [27].. This study included adults 18 years and above, who live in Bolgatanga and accepted the consent to participate. This study excluded individuals less than 18 years and adults who are seriously ill or mentally unfit to participate in the research and patients who did not give consent.

Profile of study area

This study was confined to the settings of Bolgatanga. Bolgatanga Municipal is in the center of the Upper East Region and is the regional capital. It has a total land area of 729 sq. km and is bordered to the north by the Bongo District, to the south and east by Talensi, and to the west by the Kassena-Nankana Municipality. The municipality was established by LI 1797 (2004) [28]. As of 2021, the town had a population of about 142,509 people, with females (74,659) representing 52.4% and males (67,850) representing 47.6 %. The municipality is divided into nine (9) sub-municipalities, namely Bolga Central, Bolga North, Bolga South, Kalbeo, Sherigu, Sumbrungu East, Sumbrungu West, Plaza and Zaare. The capital of the municipality is Bolgatanga, which is cosmopolitan. The major tribe is Frafra, with significant tribes from within the Upper East Region, as well as other neighbouring regions in the country. The climate is classified as tropical and has two distinct seasons, the rainy season that runs from May to October and a long dry season that stretches from October to April, with hardly any rain. Mean annual rainfall is 950 mm, while the maximum temperature is 45°C in March and April with a minimum of 12°C in December[28]. The selection of this study area is influenced by inconsistent utilization patterns across different PHC facilities in the municipality observed by the health workers.

Theoretical Model/Framework

This study employed Andersen's Behavioral Model of Health Services Use as the theoretical framework to explain the key factors that influence individual preferences for PHC facility type in Bolgatanga Municipality, Upper East Region, Ghana.

In the context of this study, the model's enabling and predisposing domains directly inform both the research design and the selection of variables. Specifically, the accessibility factors examined, namely distance to facility, transportation options, and cost of service, are theoretically situated within Andersen's enabling domain, as they constitute the structural and economic conditions that either facilitate or impede an individual's capacity to reach and engage with a preferred PHC facility type [29, 31]. Concurrently, the healthcare service quality dimensions encompassing waiting time, cleanliness, communication with provider, and drug availability align predominantly with Andersen's predisposing and enabling constructs, as they reflect individuals' evaluative perceptions of the care environment that shape attitudinal dispositions and reinforce or redirect facility preferences over repeated health-seeking episodes. The model's conceptual logic further justifies the adoption of a cross-sectional research design, as Andersen's framework is fundamentally a snapshot model of utilization behavior. It theorizes the simultaneous interplay of predisposing, enabling, and need conditions at a given point in time rather than tracing longitudinal behavioral trajectories, making a cross-sectional survey the most epistemologically coherent design for capturing and measuring the concurrent influence of these variable domains on facility-type preference. In the study preferred facility types included in the study are District hospital, Private clinic/hospital/Faith based, Government health centers or polyclinic and CHPS compound. District hospitals were included because, within the Ghana Health Service referral framework, they often serve as the first point of contact for many communities and provide a substantial proportion of primary healthcare services, particularly in areas like Bolgatanga, where lower-level facilities have limited capacity. Respondents were asked about their preferred facility for seeking primary healthcare services rather than hypothetical choices. Analytically, the model's tripartite structure guides the organization of variables into theoretically meaningful blocks, informing a multivariate analytical approach, most appropriately multinomial logistic regression, wherein accessibility factors and service quality dimensions are examined as independent predictors of facility-type preference, consistent with Andersen's proposition that utilization outcomes are the product of the additive and interactive influence of these determinant domains. Consequently, far from being a passive theoretical reference, Andersen's Behavioral Model of

Health Services actively structures every methodological decision in this study, from what is measured and why to how variables are categorized and the manner in which their relationships with PHC facility-type preferences are estimated and interpreted, thereby ensuring that the study's empirical inquiry is theoretically grounded, internally coherent, and meaningfully positioned within the broader canon of health services utilization research [29, 30].

Sampling methods

The optimal sample size was determined using the Yamane (1973) formula which assumes simple random sampling [32], with population (N = 142,509), and margin of error (e = 0.05). A total of 398 accurate and complete questionnaires were obtained, which matched the actual sample size estimated without the nonresponse rate. For this study, the researcher used a three-stage cluster sampling method. The absence of design effect adjustment is due to the fact that, the primary objective of the study was to obtain population-level estimates and examine associations among variables rather than produce highly precise cluster-level estimates. Though the use of random selection at each sampling stage helped to reduce selection bias, the use of multistage cluster sampling may have introduced intra-cluster correlation, which could result in larger standard errors and slightly reduced statistical precision.

In the first stage, the study area was divided into clusters based on the nine (9) sub-municipalities, namely Bolga Central, Bolga North, Bolga South, Kalbeo, Sherigu, Sumbrungu East, Sumbrungu West, Plaza, and Zaare. From these, 5 clusters (Bolga Central, Bolga South, Sherigu, Sumbrungu East, and Plaza) were randomly selected. In the second stage, the researchers randomly selected compounds from each selected cluster by assigning codes with the aid of random number generator software. The researcher then listed all the households in each selected compound. A household here means a father, mother, and children or relatives under one roof, so a compound may have more than one household. Sub-municipalities may differ substantially in socioeconomic status, education, ethnicity, and urbanization, and households located in compounds of different sizes may have unequal probabilities of selection. To address this, the number of compounds selected per sub-municipality was proportional to the total number of compounds identified in that cluster, thereby minimizing selection bias arising from compound size heterogeneity. From the 250 selected compound households, the researcher selected not more than two adults from each household at random to take part in the study at the third stage. Here, and with the aid of random number generator

software, the participants were assigned a code and surveyed. This method made the process fair and ensured that every household has an equal chance of being included.

Data collection techniques

Data for this study was collected using researcher-administered questionnaires. A novel questionnaire was developed by the researchers specifically for the purposes of this study and uploaded under the supplementary file. These were administered through face-to-face interviews, which helped to ensure accurate responses from participants who had difficulties reading or understanding the questions independently. The questionnaire developed for this study underwent a rigorous content validity process, whereby five experts in health research from the department of Health Policy, Management, and Economics, KNUST independently evaluated each item for relevance, clarity, and representativeness using a Content Validity Index (CVI), yielding an item-level CVI (I-CVI) of ≥0.80 and a scale-level CVI (S-CVI) of 0.92, consistent with established thresholds for acceptable content validity. To further establish instrument reliability, a pilot test was conducted with 20 respondents drawn from Tafo community, Kumasi. The pilot exercise focused on instrument refinement rather than context-specific behavioural measurement; therefore, emphasis was placed on linguistic clarity and interpretability of items rather than contextual equivalence of responses. However, we recognize that Kumasi is socio-culturally and geographically different from the study area (Bolgatanga), and this may limit the extent to which contextual nuances were captured during pretesting. Also, internal consistency was measured using a Cronbach's alpha, where all construct yielded a coefficient ≥ 0.70. This confirms satisfactory internal consistency of the self-reported items. Construct validity was additionally supported via expert panel feedback, which was used iteratively to refine item wording for interpretability and contextual appropriateness prior to final administration.

Data analysis

The data was entered and analyzed using Stata Version 17.0 and SPSS Version 22.0. SPSS Version 22.0 was used for descriptive statistics and multinomial logistic regression, while Stata Version 17 was used for additional model diagnostics including VIF estimation. Descriptive data were presented using tables, frequencies, percentages, and charts where necessary. Initial bivariate analysis was conducted to examine crude associations between healthcare facility preference and all independent variables, including healthcare service and accessibility factors. Specifically, Chi-square tests were used for categorical variables to identify statistically

significant relationships at the bivariate level ($p < 0.05$), consistent with standard modelling procedures in health services research. Variables that demonstrated theoretical relevance or significant crude associations were subsequently considered for inclusion in the multivariable model to control for potential confounding effects and improve the robustness of parameter estimates. Multinomial logistic regression was also used to determine the role of healthcare service quality and accessibility factors in shaping individual preferences for healthcare facility type. The model diagnostic tests revealed a Cox & Snell Pseudo R^2 value of 0.341 for perceived-quality-of-care and 0.431 for accessibility factors, indicating that the independent variables collectively explain approximately 34.1% and 43.1% of the variation in PHC facility-type preference and the two models respectively. The Variance Inflation Factor (VIF) values ranging from 1.198 to 1.523 and corresponding tolerance values between 0.657 and 0.835 for all the models are all well within acceptable thresholds, confirming the absence of multicollinearity among the predictor variables, thereby affirming the statistical integrity of the regression estimates. Statistical significance for all testing was set at 0.05.

Ethics approval

Ethical approval for this study was obtained from the Committee on Human Research, Publications, and Ethics (CHRPE), KNUST, in relation to the Ghana Data Protection Act, 2012 (Act 843) national guidelines, with the reference number CHRPE/AP/011/26. The study was conducted in accordance with the ethical principles outlined in the Declaration of Helsinki. All participants were informed about the purpose of the study, assured of confidentiality, and provided written informed consent prior to participation. All participants were subject to freely given, informed consent to participate in the study. Participation was voluntary, and respondents could withdraw at any time without consequence.

Results

Socio-demographic characteristics of the respondents

Table 1 provides a summary of the socio-demographic characteristics of the 398 respondents. The results show that the most respondents had completed senior high school (32.2%), whereas the least had no formal education (18.8%). In terms of marital status, the majority of the respondents were married (53.8%), and only a small proportion were cohabiting (1.0%). Christianity was the dominant religion (59.5%), while those with no religion formed the smallest group (2.0%). By occupation, traders constituted the largest category (25.9%), whereas those in the other category (retired, security guard, and hairdresser) accounted for the

smallest share (3.8%). Income distribution showed that most respondents earned GHC 200–1500 (41.0%), whereas only 4.0% earned GHC 5001–7000. Finally, NHIS status revealed that most respondents were registered and active members (74.9%), while the least were not registered (3.8%). The respondents had a mean age of 39.5 years with standard deviation of 12.3.

Table 1: Socio-demographic characteristics of the respondents

Variable		Frequency (N=398)	Percentage (%)
Highest level of education	No formal education	75	18.8
	Basic (Up to JHS)	83	20.9
	Senior High School (SHS)	128	32.2
	Tertiary (Diploma, HND, Degree)	112	28.1
Marital status	Single	155	38.9
	Married	214	53.8
	Cohabiting	4	1.0
	Divorced/Separated	13	3.3
	Widowed	12	3.0
Religion	Christianity	237	59.5
	Islam	103	25.9
	Traditional Religion	50	12.6
	No religion	8	2.0
Occupation	Unemployed	83	20.9
	Farmer	45	11.3
	Trader	103	25.9
	Civil/Public servant	75	18.8
	Private sector worker	22	5.5
	Artisan (e.g., carpenter, mason)	22	5.5
	Student	33	8.3
	Other	15	3.8
Income	GHC200-1500	163	41.0
	GHC1501-3000	83	20.9
	GHC3001-5000	136	34.2
	GHC5001-7000	16	4.0
NHIS Status	Not Registered	15	3.8
	Registered but not active	85	21.4
	Registered and active membership	298	74.9
**Variable**	**Mean (Std. Deviation)**		
Age in Years	**39.5 (12.3)**		

The role of healthcare service quality in shaping individual preferences for PHCfacility type

Table 2 presents a descriptive analysis of the quality-of-care, preferred healthcare facility type, and factors influencing respondents' healthcare choices. Among the 398 respondents, most

rated the quality-of-care as good (45.7%), whereas very few rated it as poor (0.5%) or excellent (1.0%). For service use, the majority preferred facilities for routine check-ups (62.8%), whereas the smallest proportion visited for specialized treatment (9.3%). District hospitals were the most preferred PHC facilities (43.2%), while private clinics, faith-based facilities, and CHPS compounds were the least preferred (12.8% each). Across the influencing factors, most respondents consistently rated quality-of-care (76.4%), waiting time (77.1%), staff attitude (75.1%), availability of follow-up care (70.1%), specialized services (68.8%), cost (57.0%), medicine availability (67.3%), and clean environment (70.9%) as important, with very few considering these factors not important. Among influence-related variables, personal recommendation (77.4%), facility reputation (74.6%), insurance coverage (75.9%), and accessibility (76.1%) were rated as highly influential, whereas online reviews were rated moderately influential (59.0%), making them the least influential factor overall.

Tables 3 and 4 present a multinomial logistic regression result examining the association between quality-of-care and the type of healthcare facility preferred, obtained from SPSS V.22 software. The model was statistically significant ($\chi^2(36) = 208.71$, $p < .001$), indicating that the predictors jointly explained a meaningful proportion of variance in facility choice. The multinomial logistic regression results indicate that quality-of-care significantly influences respondents' preferences for PHC type after adjusting for age, education, NHIS status, and cost.

For private clinics/faith-based facilities, respondents who rated quality-of-care as very important compared to not important are 74% more likely to prefer for private clinics/faith-based facilities compared to CHPS compounds (aOR = 1.74, 95% CI: 1.02–9.73, p = 0.026).

Among those who considered quality-of-care important rather than not important were 56% higher (aOR = 1.56, 95% CI: 1.05–2.91, p = 0.008). Both respondents who rated quality-of-care as important (aOR = 2.89, 95% CI: 1.02–9.78, p = 0.033) and very important (aOR = 1.25, 95% CI: 1.01–3.61, p = 0.004) had higher odds of preferring district hospitals over CHPS compounds.

Table 2: Descriptive analysis of quality-of-care and preferred PHCfacility type

Variable		Frequency(f)	Percentage (%)
Quality-of-care	Poor	2	0.5
	Good	182	45.7
	Fair	128	32.2
	Very Good	82	20.6
	Excellent	4	1.0
Healthcare facility preferred for	Routine check-ups	250	62.8
	Emergency care	111	27.9
	Specialized treatment	37	9.3
Preferred PHC facility types	District hospital	172	43.2
	Private clinic/hospital/Faith based	51	12.8
	Government health centers or polyclinic	124	31.2
	CHPS compound	51	12.8
**TOTAL (n)**		**398**	**100%**

Table 3: Descriptive analysis of factors influencing individual preferences for healthcare facility type

Factor	Not Important	Neutral	Important	Very Important
Quality-of-care	0 (0.0%)	13 (3.3%)	304 (76.4%)	81 (20.4%)
Waiting time	0 (0.0%)	9 (2.3%)	306 (77.1%)	82 (20.7%)
Staff attitude & communication	0 (0.0%)	17 (4.3%)	299 (75.1%)	82 (20.6%)
Availability of follow-up care	19 (4.8%)	27 (6.8%)	279 (70.1%)	73 (18.3%)
Availability of specialized services	13 (3.3%)	33 (8.3%)	274 (68.8%)	78 (19.6%)
Cost	0 (0.0%)	23 (5.8%)	225 (57.0%)	147 (37.2%)
Availability of medicines	0 (0.0%)	12 (3.0%)	268 (67.3%)	118 (29.6%)
Clean environment	2 (0.5%)	22 (5.5%)	282 (70.9%)	92 (23.1%)
	**Not Influential**	**Neutral**	**Influential**	**Very Influential**
Personal recommendations	4 (1.0%)	36 (9.0%)	308 (77.4%)	50 (12.6%)
Online reviews	12 (3.0%)	107 (26.9%)	235 (59.0%)	44 (11.1%)
Facility reputation	6 (1.5%)	39 (9.8%)	297 (74.6%)	56 (14.1%)
Insurance coverage	0 (0.0%)	14 (3.5%)	302 (75.9%)	82 (20.6%)
Accessibility and location	0 (0.0%)	16(4.1%)	300(76.1%)	78(19.8%)
		**n=398(100%)**		

Table 4: Multinomial logistic regression of the association between quality-of-care and preferred PHC facility type

Variable		Odds (95% CI)	p-value
**Private clinic / Faith-based vs CHPS**			
Quality-of-care	Not Important	**Ref**	
	Important	0.95 (0.240, 5.68)	0.994
	Very Important	1.74 (1.02, 9.73)	0.026
Age		2.37 (1.02, 8.04)	0.033
Education	No formal education	**Ref**	
	Basic	0.90 (0.25, 3.29)	0.872
	SHS	2.41 (0.69, 8.45)	0.166
	Tertiary	4.83 (2.90, 9.67)	0.027
NHIS status	Not registered	**Ref**	
	Registered, not active	3.05 (0.34, 27.70)	0.322
	Registered, active	0.76.07 (0.13, 0.97)	0.003
Cost	Not Important	Ref	
	Important	0.68 (0.09, 5.36)	0.723
	Very Important	0.17 (0.01, 0.73)	0.029
**Government health centre / Polyclinic vs CHPS**			
Quality-of-care	Not Important	**Ref**	
	Important	1.56 (1.05, 2.91)	0.008
	Very Important	0.46 (0.13, 0.89)	0.992
Age		1.72 (1.21, 9.99)	<0.001
Education	No formal education	**Ref**	
	Basic	0.61 (0.22, 1.69)	0.345
	SHS	1.64 (0.60, 4.49)	0.334
	Tertiary	3.76 (0.88, 16.02)	0.074
NHIS status	Not registered	**Ref**	
	Registered, not active	1.90 (0.29, 10.41)	0.505
	Registered, active	3.26 (0.57, 16.96)	0.183
Cost	Neutral	**Ref**	
	Important	5.45 (1.55, 10.83)	0.025
	Very Important	3.02 (0.76, 29.65)	0.341
**District hospital vs CHPS**			
Quality-of-care	Not Important	**Ref**	
	Important	2.89(1.02, 9.78)	0.033
	Very Important	1.25 (1.01, 3.61)	0.004
Age		2.82 (1.01, 9.73)	< 0.001
Education	No formal education	**Ref**	
	Basic	1.21 (0.40, 4.37)	0.733
	SHS	3.03 (1.01, 9.09)	0.047
	Tertiary	14.08 (3.19, 62.02)	< 0.001
NHIS status	Not registered	**Ref**	
	Registered, not active	2.96 (0.45, 19.56)	0.259

	Registered, active	2.53 (0.45, 14.19)	0.295
Cost	Not Important	**Ref**	
	Important	1.33 (0.07, 9.63)	0.779
	Very Important	0.61 (0.09, 0.98)	0.023

NB: Ref = Reference category

Effect of accessibility factors on individual preferences for healthcare facility type

Table 5 provides a descriptive analysis of accessibility factors and barriers to healthcare services among the respondents. Among the 398 respondents, the majority had used healthcare services in the past six months (68.1%), whereas a smaller proportion had not (31.9%). Among the 271 individuals who had accessed care, the majority relied on both public and private facilities (57.6%), with fewer depending solely on public or private facilities (10.7%). Location played a major role in healthcare decisions, with most respondents (74.9%) indicating that it was influential, while only a small fraction found it not influential (1.0%). Willingness to travel varied by service type: most respondents were willing to travel further for specialized care (82.4%) and emergency care (60.8%), but willingness dropped for routine care, where slightly more than half (51.3%) were unwilling. Regarding barriers, most respondents reported no barriers (69.3%). Among the 122 who experienced challenges, cost emerged as the most common barrier (80.3%), followed by long waiting times (57.4%) and distance (32%).

Tables 6 and 7 present the results of a multinomial logistic regression examining the influence of accessibility factors on the type of PHC facility preferred from SPSS V.22 software. The model was statistically significant: $\chi^2(43) = 109.66$, $p < .001$ (indicating that distance, transportation options, waiting time, appointment availability, and cost jointly explained a meaningful proportion of variance in facility choice). Specifically, respondents who rated distance and location as very important rather than not important were 242% more likely to choose private clinics/faith-based facilities rather than CHPS compounds (aOR = 3.42, 95% CI: 1.78–6.84, p = 0.001). Those who considered transportation options as very important compared to not important were 301% more likely to select private clinics/faith-based facilities rather than CHPS compounds (aOR = 4.01, 95% CI: 2.10–7.83, $p < 0.001$). Respondents who rated waiting time as very important compared to not important are 21% more likely to choose private clinics/faith-based facilities compared to CHPS compounds (aOR = 1.21, 95% CI: 1.10–2.47, $p < 0.001$), while those who considered appointment availability as very important are 131% more likely to prefer these facilities compared to CHPS compounds (aOR = 2.31, 95% CI: 1.14–5.61, p = 0.001). Conversely, respondents who rated cost of care as very

important are 12% less likely to choose private clinics/faith-based facilities compared to CHPS compounds (aOR = 0.88, 95% CI: 0.02–0.95, $p < 0.001$).

For government health centres or polyclinics, respondents who rated distance and location as very important are 134% more likely to select these facilities compared to CHPS compounds (aOR = 2.34, 95% CI: 1.24–4.56, p = 0.009), and those who considered transportation options as very important are 248% more likely to prefer them (aOR = 3.48, 95% CI: 1.82–6.68, $p < 0.001$). In contrast, respondents who rated waiting time as very important are 71% less likely to choose government facilities (aOR = 0.29, 95% CI: 0.14–0.55, p = 0.001), and those who considered appointment availability as very important are 66% less likely to select these facilities (aOR = 0.34, 95% CI: 0.17–0.66, p = 0.002). Respondents who rated the cost of transportation as very important are 171% more likely to prefer government health centres or polyclinics (aOR = 2.71, 95% CI: 1.40–5.37, p = 0.003). Regarding district hospitals, respondents who rated distance and location as very important are 355% more likely to choose district hospitals compared to CHPS compounds (aOR = 4.55, 95% CI: 2.09–8.91, p = 0.510), and those who considered transportation options as very important are 498% more likely to select them (aOR = 5.98, 95% CI: 0.94–11.85, p = 0.700). Conversely, respondents who rated waiting time as very important are 83% less likely to choose district hospitals (aOR = 0.17, 95% CI: 0.08–0.33, $p < 0.001$), while those who considered appointment availability as very important are 68% less likely to select these facilities (aOR = 0.32, 95% CI: 0.16–0.62, p = 0.001). Respondents who rated cost of care as very important are 318% more likely to prefer district hospitals (aOR = 4.18, 95% CI: 2.11–8.21, $p < 0.001$).

Table 5: Descriptive analysis of accessibility factors and barriers to accessing healthcare services

Item		Frequency (f)	Percentage (%)
Have you used healthcare services in the past 6 months?	Yes	271	68.1
	No	127	31.9
**TOTAL**		**398**	**100%**
If yes, what type of healthcare facility do you typically use?			
	Public	86	31.7
	Private	29	10.7
	Both private and pubic	156	57.6
**TOTAL**		**271**	**100%**
How does the location of a healthcare facility influence your decision to use it?	Not Influential at All	25	6.3
	Not Influential	4	1.0
	Neutral	41	10.3
	Influential	298	74.9
	Very Influential	30	7.5
Would you be willing to travel further for Specialized care	No	70	17.6
	Yes	328	82.4
Would you be willing to travel further for Emergency care	No	156	39.2
	Yes	242	60.8
Would you be willing to travel further for Routine care	No	204	51.3
	Yes	194	48.7
Have you experienced any barriers to accessing healthcare services?	Yes	122	30.7
	No	276	69.3
**TOTAL**		**398**	**100%**
If yes what were the barriers (Multiple responses) a. transportation issues	No	112	91.8
	Yes	10	8.2
b. Long waiting times	No	52	42.6
	Yes	70	57.4
c. Cost	No	24	19.7
	Yes	98	80.3
d. Distance	No	83	68.0
	Yes	39	32.
e. Other (No drugs, community conflict)	No	118	96.7
	Yes	4	3.3
**TOTAL**		**610**	**100%**

Table 6: Multinomial logistic regression of accessibility factors influencing preferred PHC facility type

Variable	Odds (95% CI)	P-value	
**Private clinic / Faith-based vs CHPS**			
Distance & Location	Not Important	**Ref**	
	Important	1.88 (1.10, 3.57)	0.042
	Very Important	3.42 (1.78, 6.84)	0.001
Transportation Options	Not Important	Ref	
	Important	2.15 (1.24, 4.02)	0.009
	Very Important	4.01 (2.10, 7.83)	< 0.001
Waiting Time	Not Important	**Ref**	
	Important	1.54 (1.09, 2.97)	0.021
	Very Important	1.21 (1.10, 2.47)	< 0.001
Appointment Availability	Not Important	**Ref**	
	Important	1.48 (0.26, 0.91)	0.024
	Very Important	2.31 (1.14, 5.61)	0.001
Cost of care	Not Important	Ref	
	Important	0.74 (0.11, 0.99)	0.028
	Very Important	0.88 (0.02, 0.95)	< 0.001
**Government Health Centre / Polyclinic vs CHPS**			
Distance & Location	Not Important	**Ref**	
	Important	1.72 (1.04, 3.14)	0.049
	Very Important	2.34 (1.24, 4.56)	0.009
Transportation Options	Not Important	**Ref**	
	Important	2.28 (1.29, 4.26)	0.006
	Very Important	3.48 (1.82, 6.68)	< 0.001
Waiting Time	Not Important	Ref	
	Important	0.47 (0.26, 0.82)	0.008
	Very Important	0.29 (0.14, 0.55)	0.001
Appointment Availability	Not Important	**Ref**	
	Important	0.49 (0.27, 0.91)	0.024
	Very Important	0.34 (0.17, 0.66)	0.002
Cost of Transportation	Not Important	**Ref**	
	Important	1.62 (0.95, 3.21)	0.071
	Very Important	2.71 (1.40, 5.37)	0.003

NB: Ref = Reference category

Table 7: Multinomial logistic regression of accessibility factors influencing preferred PHC facility type cont'd

Variable		Odds (95% CI)	P-value
**District Hospital vs CHPS**			
Distance & Location	Not Important	**Ref**	
	Important	0.35 (0.27, 0.79)	0.008
	Very Important	4.55 (2029, 8.91)	0.510
Transportation Options	Not Important	**Ref**	
	Important	0.68 (0.02, 0.95)	0.002
	Very Important	5.98 (0.94, 11.85)	0.700
Waiting Time	Not Important	**Ref**	
	Important	0.39 (0.21, 0.70)	0.002
	Very Important	0.17 (0.08, 0.33)	< 0.001
Appointment Availability	Not Important	**Ref**	
	Important	0.50 (0.27, 0.94)	0.032
	Very Important	0.32 (0.16, 0.62)	0.001
Cost of care	Not Important	**Ref**	
	Important	2.20 (1.13, 4.28)	0.020
	Very Important	4.18 (2.11, 8.21)	< 0.001

NB: Ref = Reference category

Discussions

Perceived quality-of-care emerged as a determinant of individuals' preference for PHC facility type in the Bolgatanga Municipality. In specific contexts, private or faith-based facilities, government health centers or polyclinics, and district hospitals rather than CHPS compounds were preferred by individuals who attributed greater importance to quality-of-care. This shows that higher-level or alternative facilities that are perceived to offer better services are opted for due to higher valuation of quality. Also, individuals assess the standard of care they anticipate receiving and justify their facility choice with perceptions of competence, responsiveness, and service reliability beyond mere physical access. This shows a growing demand for healthcare services that are not only accessible but also trustworthy, patient-centred, and effective. Through the assumptions of Andersen's Behavioral Model of Health Services Use, the findings reflect the interaction between predisposing, enabling, and need factors influencing an individual's preference for healthcare facility type [31]. Here, the perceived quality-of-care operates as 1) a predisposing factor, through individual beliefs and expectations about healthcare. 2) a need-related factor, as patients seek facilities they believe can adequately address their health problems [29, 30]. This shows that the choice of hospitals

and private facilities among those who highly value quality-of-care suggests that when enabling resources are available, perceived quality becomes a decisive driver of utilization.

The current finding is supported by Bahrampour et al. [14] who report that service quality in terms of low waiting time, staff attitude, cleanliness, and follow-up care influenced patients' hospital preferences in Iran. In the same way, Gabrani et al. [33] supported that both public and private healthcare users in Albania prioritized quality-of-care when choosing facilities. Gage et al. [34] showed that perceptions of provider responsiveness and service quality shaped utilization of PHC facilities in Haiti. Studies in Africa by Mwami and Oleche [35] and Lokina and Musili [15] in supporting this current finding reported that perceived staff competence, shorter waiting times, and better interpersonal treatment drove patients toward private clinics in Kenya. Olorunfemi et al. [16] in similar context observed similar preferences for faith-based facilities in Nigeria due to better provider–patient interactions. On the other hand, Mhlanga and Hassan [36] found that cost considerations outweighed perceived quality among low-income households in South Africa. And this leads to continued preference for public facilities despite dissatisfaction. Also, the current findings showed that while the highest education level (Odds = 3.76, p = 0.074) was not significantly related to the choice of government health centre, NHIS status was also not significantly associated with both government health centers (Odds = 3.26) and district hospitals (Odds = 2.53). While these can be explained by structural and supply-side barriers such as facility availability, perceived quality, and proximity that may override individual-level socioeconomic characteristics in driving healthcare-seeking decisions in resource-constrained settings like Bolgatanga, the methodological limitations of this study are of concern. Methodologically, the cross-sectional design precludes any causal inference, as both exposure and outcome are measured simultaneously, making it impossible to determine whether education or NHIS status precedes or follows facility preference. The use of multinomial logistic regression, while appropriate for polytomous outcomes, is sensitive to small cell sizes across facility categories, which may have inflated standard errors and reduced statistical power. This is a particularly acute concern, as the analysis is geographically confined to Bolgatanga municipality alone, limiting both sample diversity and the generalizability of findings to other urban, peri-urban, or rural contexts in the Upper East Region or Ghana broadly. Despite the demerits, these findings suggest the need to strengthen quality-of-care at the primary level, particularly within CHPS compounds. Future studies should adopt larger, multi-site probability samples across diverse districts, incorporate longitudinal designs to track

how NHIS enrollment dynamically influences facility choice over time, and consider multilevel modeling to disentangle individual-level from facility-level determinants.

In Bolgatanga, accessibility-related factors such as distance and location, transportation options, waiting time, appointment availability, and cost considerations were found to relate to individuals' preferences for PHC facility type. This shows that multiple accessibility factors interact in individuals' daily lives in selecting facility type and that healthcare choices are not driven by a single access. From the study, individuals who valued proximity and transportation convenience preferred private clinics, faith-based facilities, and district hospitals. This may suggest that patients are willing to bypass nearby CHPS compounds when other facilities are perceived to be easier to reach or better connected by transport. Interestingly, dissatisfaction with congestion and delays commonly associated with higher-level public facilities were revealed in the study. Where participants who valued shorter waiting times and easier appointment availability were less likely to choose hospitals and government facilities. Also, the persistent relationship between financial barriers and an individual's healthcare behavior, particularly in low-resource settings, was revealed, where respondents who prioritized cost showed a lower preference for private facilities. In the context of this study, Andersen's Behavioral Model of Health Services Use, the findings relate to the central role of enabling factors in healthcare utilization. Thus, distance, transportation options, waiting time, appointment systems, and cost represent key enabling or constraining conditions that determine whether individuals can translate their healthcare needs into actual service use [29, 30].

Studies in India and the United States support the current findings by showing that distance, travel time, and transportation barriers significantly relate to facility choice, with rural populations often preferring nearby primary care facilities due to mobility constraints [18, 19] . Guimarães et al. [20] and Akkazieva and Zhao [21] also support the current findings, where they reported that financial accessibility often overrides geographical proximity, as low-income households avoid facilities perceived as costly even when they are close. Studies in Africa such as Mangundu et al. [22] and Oriyomi [23] support the current findings, documenting how long distances, poor road networks, and transport costs push rural households toward closer or informal healthcare options. In Ghana, Asante [24] and Otchere et al. [25] showed that proximity and travel time encourage the use of CHPS compounds, which support the current findings. Asare-Akuffo et al. [37] in supporting the current findings, found that individuals may travel longer distances when they perceive higher-level facilities as more capable of

addressing their health needs. The identification of proximity and transportation convenience as drivers of preference for private clinics, faith-based facilities, and district hospitals particularly adds nuance to demand-side analyses in low-resource urban settings. Nonetheless, the study's cross-sectional, single-municipality design represents a significant methodological limitation. It captures a static snapshot of preferences without accounting for seasonal variation in access, such as during rainy seasons when transportation is disrupted, and the geographically bounded sample constrains transferability of findings beyond Bolgatanga's specific infrastructural and socioeconomic context. Furthermore, reliance on self-reported preferences may introduce social desirability bias, and the absence of objective facility-level data such as actual travel distances and verified waiting times limits triangulation of subjective perceptions against measurable access indicators. Despite the limitations, the study suggests the need for integrated interventions that address both physical and organizational access to care. That is, enhancing road networks, strengthening public transport systems, and decentralizing services could reduce the burden of distance and transportation barriers. Future research should employ mixed-methods longitudinal designs across multiple municipalities, integrating Geographic Information Systems (GIS) mapping of facility catchment areas with qualitative exploration of how accessibility trade-offs are negotiated across different household types and seasons.

Conclusions

The study aimed to identify the factors associated with individuals' preferences for PHC facility type in the Bolgatanga Municipality, Upper East Region, Ghana. We found that quality-of-care and accessibility-related factors such as distance and location, transportation options, waiting time, appointment availability, and cost considerations were associated with individuals' preferences for PHC facility type. Therefore, the study suggests that healthcare managers in Bolgatanga and the Ministry of Health should prioritize interventions aimed at improving quality-of-care, such as improving interpersonal communication between health workers and patients, enhancing staff training, ensuring consistent availability of medications, and maintaining a hygienic and safe facility environment across all facility types. They should also provide effective and reliable transportation options, optimizing the location of new facilities, reducing waiting times through appointment management systems, and ensuring reasonable service fees. By addressing these quality-of-care gaps, public and CHPS facilities may become more attractive to residents who currently prefer private or faith-based facilities due to perceived superior service. Also, with these measures, barriers that currently drive patients

toward higher-level facilities while maintaining affordability for low-income populations would reduce.

For methodological enhancement, future studies should adopt larger, multi-site probability samples across diverse districts, incorporate longitudinal designs to track how NHIS enrollment dynamically influences facility choice over time, and consider multilevel modeling to disentangle individual-level from facility-level determinants. Also, future research should employ mixed-methods longitudinal designs across multiple municipalities, integrating Geographic Information Systems (GIS) mapping of facility catchment areas with qualitative exploration of how accessibility trade-offs are negotiated across different household types and seasons. Also, linking survey data with routine health information systems would provide stronger behavioural validation.

Authors' Contributions

S.A., C.O., and K.A.M. conceptualized and designed the study. S.A. and L.A. collected the data under the supervision of K.A.M. and L.A., and CO analyzed and interpreted the data and wrote the draft manuscript. All authors read and approved the final manuscript.

Acknowledgement

Not Applicable

References

1. Zhang W, Ung COL, Lin G, Liu J, Li W, Hu H, et al. Factors contributing to patients' preferences for primary health care institutions in China: a qualitative study. Frontiers in public health, 8, 414 Aday. L A, & Andersen, R. 2020;9(3):208–20.

2. WHO. Key Facts: Primary health care [Internet]. 2025 [cited 2025 Jun 11]. Available from: https://www.who.int/news-room/fact-sheets/detail/primary-health-care

3. Ministry of Health G. Ghana health sector medium-term development plan 2022–2025. Ministry of Health; 2020.

4. Allen LN, Pettigrew LM, Exley J, Nugent R, Balabanova D, Villar-Uribe M, et al. The role of Primary Health Care, primary care and hospitals in advancing Universal Health Coverage. BMJ global health. 2023;8(12).

5. Shyaka A, Igihozo G, Tegli M, Ntiyaduhanye E, Ndizeye E, Umuhoza A, et al. Rwanda One health achievements and challenges: the final push toward policy implementation. One Health Cases. 2024;20240010.

6. Renggli S, Mayumana I, Mboya D, Charles C, Mshana C, Kessy F, et al. Towards improved health service quality in Tanzania: appropriateness of an electronic tool to assess quality of Primary Health Care. BMC health services research. 2019;19:1–16.

7. Armah P, Kicha D. Primary health care in Ghana: the structure and functions in relation to preventing neglected tropical diseases. Archiv Euromed. 2020;10:12–7.

8. Ghana Health Service. Ministry of Health 2020 Annual Performance Report [Internet]. Gana: National Development Planning Commission; 2020 [cited 2025 Jun 11]. Available from: https://www.ndpc.gov.gh/media/Ministry_of_Health_APR_2020_XT2Z015.pdf

9. Kraef C, Juma P, Kallestrup P, Mucumbitsi J, Ramaiya K, Yonga G. The COVID-19 pandemic and non-communicable diseases—a wake-up call for primary health care system strengthening in sub-Saharan Africa. Journal of primary care & community health. 2020;11.

10. Asenso-Boadi F, Aikins M, Agyepong IA. Bypassing closer health services: A study of patients visiting a district hospital in Ghana. Global Health Action. 2016;9(1):31157. doi:10.3402/gha.v9.31157

11. Tuoyire DA, Baatiema L, Dwomoh D. Healthcare utilization in Ghana: Insights from the Ghana Living Standard Survey. PLOS ONE. 2017;19(6):e0306032. doi:10.1371/journal.pone.0306032

12. Farhan A, Aryeetey GC. Factors influencing utilization of healthcare services in Danku in the upper west region of Ghana: a mixed method study. J Glob Health Sci. 2024;6(2):e15. doi:10.35500/jghs.2024.6.e15

13. Senapati S, Panda RK. Enriching healthcare service quality by leveraging service experiences in Indian private hospitals: application of fuzzy-AHP approach. International Journal of Quality & Reliability Management. 2023;40(10):2301–25.

14. Bahrampour M, Bahrampour A, Amiresmaili M, Barouni M. Hospital service quality–patient preferences–a discrete choice experiment. International journal of health care quality assurance. 2018;31(7):676–83.

15. Lokina R, Musili W. Household’s Choice of Healthcare Provider in Kenya. The African Review. 2021;48(1):239–62.

16. Olorunfemi O, Agbaje OC, Abiodun OO, Ayeni BA, Osunde RN. Understanding the Roles of Religion in Nursing Practice as a Prescriptive Instrument for High-Quality Faith-Based Healthcare. Amrita Journal of Medicine. 2024;20(4):144–8.

17. Atinga RA, Abekah-Nkrumah G, Domfeh KA. Managing healthcare quality in Ghana: a necessity of patient satisfaction. International journal of health care quality assurance. 2020;24(7):548–63.

18. Verma VR, Dash U. Geographical accessibility and spatial coverage modelling of public health care network in rural and remote India. PloS one. 2020;15(10):e0239326.

19. Syed ST, Gerber BS, Sharp LK. Traveling towards disease: transportation barriers to health care access. Journal of community health. 2023;38:976–93.

20. Guimarães T, Lucas K, Timms P. Understanding how low-income communities gain access to healthcare services: A qualitative study in São Paulo, Brazil. Journal of Transport & Health. 2019;15:100658.

21. Van Doorslaer E, O'Donnell O, Rannan-Eliya RP, Somanathan A, Adhikari SR, Akkazieva B, et al. Paying out-of-pocket for health care in Asia: Catastrophic and poverty impact. Rotterdam and IPS, Colombo: Erasmus University; 2021.

22. Mangundu M, Roets L, Janse van Rensberg E. Accessibility of healthcare in rural Zimbabwe: The perspective of nurses and healthcare users. African journal of primary health care & family medicine. 2020;12(1):e1–7. doi:10.4102/phcfm.v12i1.2245

23. Oriyomi K. Factors influencing choice of healthcare facilities utilisation by rural households in Ogun State, Nigeria. AGRICULTURA TROPICA ET SUBTROPICA. 2023;56.

24. Asante AG. Choice of Healthcare Services and Its Influencing Factors in the Sunyani Municipality [PhD Thesis]. University of Ghana; 2020.

25. Otchere G, Ankomah SE, Fusheini A, Kumah E, Agyei SK. Accessibility and utilization of health-care services among rural–urban migrants in Ghana: A scoping review. International Journal of Population Studies. 2024;10(4):1–17.

26. Saeed BII, Yawson AE, Nguah S, Agyei-Baffour P, Emmanuel N, Ayesu E. Effect of socio-economic factors in utilization of different healthcare services among older adult men and women in Ghana. BMC Health Services Research. 2016;16:1–9.

27. Cvetkovic-Vega A, Maguiña JL, Soto A, Lama-Valdivia J, Correa López LE. Cross-sectional studies. Revista de la Facultad de Medicina Humana. 2021;21(1):164–70.

28. Ghana Statistical Service. 2021 Population and housing census: National analytical report [Internet]. GSS; 2021. Available from: https://statsghana.gov.gh/

29. Andersen RM. National health surveys and the behavioral model of health services use. Med Care. 2008;46:647–53.

30. Andersen RA. Behavioral Model of Families' Use of Health Services. Chicago, Ill: Univ. of Chicago; 1968.

31. Lederle M, Tempes J, Bitzer EM. Application of Andersen's behavioural model of health services use: a scoping review with a focus on qualitative health services research. BMJ open. 2021;11(5):e045018.

32. Omair A. Sample size estimation and sampling techniques for selecting a representative sample. Journal of Health specialties. 2025;2(4):142.

33. Gabrani J, Schindler C, Wyss K. Factors associated with the utilisation of primary care services: a cross-sectional study in public and private facilities in Albania. BMJ. 2020;Open;10: e040398. doi:10.1136/bmjopen-2020-040398

34. Gage AD, Leslie HH, Bitton A, Jerome JG, Joseph JP, Thermidor R, et al. Gage Does quality influence utilization of primary health care? Evidence from Haiti Globalization and health. 2018;14:1–9.

35. Mwami MN, Oleche MO. Determinants of utilization of health care services in Kenya. International Journal of Academic Research in Business and Social Sciences. 2020;7(10):132–56.

36. Mhlanga D, Hassan A. An investigation of the factors influencing the choice of healthcare facility in South Africa. International Journal of Research in Business and Social Science. 2022;11(4):2147–4478.

37. Asare-Akuffo F, Twumasi-Boakye R, Appiah-Opoku S, Sobanjo JO. Spatial accessibility to hospital facilities: the case of Kumasi, Ghana. African Geographical Review. 2020;39(2):120–33.

Factors Associated with Individuals' Preferences for ~~Primary Healthcare~~Primary Health Care Facility Type in Bolgatanga Municipality in the Upper East Region of Ghana.

***Charles Obeng* [1]*, *Sussana Atanga* [2]*, *Kofi Akohene Mensah*[3], *Lawal Alhassan*[4]**

[1]* Department of Health Policy, Management, and Economics, Kwame Nkrumah University of Science and Technology (KNUST), Kumasi, Ghana

[2]* Bolgatanga Municipal Health Directorate, Reproductive and Child Health Unit, Bolga, Ghana.

[3]Department of Health Policy, Management and Economics, Kwame Nkrumah University of Science and Technology (KNUST), Kumasi, Ghana

[4]Bolgatanga Municipal Health Directorate, Office of Municipal Director of Health Service, Bolga, Ghana.

Corresponding Authors

[1]* Email: obengcharles436@gmail.com
[2]* Email: atangasussan85@gmail.com

Abstract

Background: There is a recognizable gap regarding the specific determinants that drive preferences for different types of ~~primary healthcare~~Primary Health Care (PHC) facilities, despite the efforts of prior scholars on factors influencing patients' preferences for facility type. To address this gap, the study identified the key factors influencing individuals' preferences for ~~primary healthcare~~ PHC facility type in the Bolgatanga Municipality, Upper East Region, Ghana.

Methods: This study employed a two-stage cluster sampling method, and 398 residents in the Bolgatanga Municipality of the Upper East Region were surveyed with structured questionnaires acting as the primary data collection tool. Data was entered and analyzed using Stata Version 17.0 and SPSS Version 22.0~~Stata Version 17.0 and SPSS Version 22.0~~ computer software. Frequencies, percentages, and multinomial logistic regression statistical analysis were employed in analyzing the filed data.

Results: ~~Quality of care~~Quality-of-care (aOR = 2.89, 95% CI: 1.02–9.78, p = 0.033) was a significant determinant of individuals' preference for ~~primary healthcare~~PHC facility type. The study also found that accessibility-related factors such as distance and location (aOR = 3.42, 95% CI: 1.78–6.84, p = 0.001), transportation options (aOR = 4.01, 95% CI: 2.10–7.83, p < 0.001), waiting time (aOR = 1.21, 95% CI: 1.10–2.47, p < 0.001), appointment availability (aOR = 2.31,

95% CI: 1.14–5.61, p = 0.001) and cost of care (aOR = 0.88, 95% CI: 0.02–0.95, p < 0.001) are associated with individuals' preferences for ~~primary healthcare~~PHC facility type.

Conclusion: The study established perceived ~~quality of care~~quality-of-care and accessibility factors significantly influenced individual preferences for ~~primary healthcare~~PHC facility type, with cost-sensitive and nearby Community-Based Health Planning and Services (CHPS) compounds ~~CHPS compounds~~ remaining crucial for clients with limited resources. Therefore, the management of facilities within Bolgatanga Municipality and similar contexts should improve service quality, reduce waiting times, and enhance transport and financial accessibility to ensure equitable utilization of ~~primary healthcare~~PHC services.

Keywords: ~~Quality of Care~~Quality-of-care; ~~Primary Healthcare~~Primary Health Care Facility Type; Distance and Location; Transportation Options; Waiting Time; Appointment Availability.

Introduction

Globally, Primary Health Care ~~primary health care~~ (PHC) has been a crucial component of the healthcare system in many countries. This level of healthcare delivery functions as~~Being~~ the first point of contact between patients and the health system, ~~the primary healthcare (PHC)~~and plays a significant role in ensuring access to essential health services [1]. According to the World Health Organization (WHO) [2], Primary Health Care (PHC) is a whole-of-society approach to health that aims to ensure the highest possible level of health and well-being and their equitable distribution by focusing on population's needs as early as possible along the continuum from health promotion and disease prevention to treatment, rehabilitation, and palliative care, as close as feasible to people's everyday environment. PHC facilities consist of a different array of settings, such as community health centers, clinics, health posts, and general practitioner practices, which function as the frontline of healthcare delivery [3]. Global declarations, such as the Alma-Ata Declaration of 1978 and the Astana Declaration of 2018, unequivocally underscore the international commitment to PHC as the most equitable, effective, and efficient path to health [2].

PHC is the most inclusive, equitable, cost-effective, and efficient approach to enhancing people's physical and mental health and social well-being. For instance, WHO [2] asserted that scaling up primary health care (PHC) interventions across low and middle-income countries could save 60 million lives and improve average life expectancy by 3.7 years by 2030. An estimated 75% of the projected health gains from the Sustainable Development Goals could be achieved through PHC [2, 4] . In Low-Middle-Income Countries (LMICs) like Rwanda, community health worker programs and health posts have optimized the transformative power of PHC that has significantly improved access to basic care and health outcomes in rural areas [5]. Also, Tanzania through the

Health Basket Fund ~~channelled~~channeled to the ~~primary healthcare~~PHC facilities has focused on strengthening its primary care infrastructure and improving service delivery at the district level, recognizing PHC as key to decentralized health governance and equitable access [6]. Similarly, in Ghana, the Community-Based Health Planning and Services (CHPS) compounds, representing the first contact of the health system, are designed to bring preventive, promotive, and basic curative services directly to users and communities, especially in rural areas, thereby improving access to immunizations, maternal and child health services, and common disease management [7]. Ghana has in previous years explicitly adopted PHC principles aimed at improving health at the basic level ~~And~~ through its National Health Insurance Scheme (NHIS). This NHIS~~,~~ aims~~ed~~ ~~at~~ to reduce~~ing~~ financial barriers to care, particularly at the primary care level.~~,~~ ~~Ghana has in previous years explicitly adopted PHC principles aimed at improving health at the basic level~~ [8]. This approach aligns with PHC's emphasis on community participation and ownership, fostering local engagement in health planning and delivery. These regional efforts demonstrate how PHC enhances health system resilience, enabling better preparedness and response to public health emergencies, as evidenced by adaptations made during the COVID-19 pandemic across the continent [9].

~~Primary healthcare~~ ~~(~~PHC~~)~~ delivery effectiveness is dependent on the efficient use of diverse facility types. And this functions as the bedrock for universal health coverage and improved health outcomes of the population [7, 10]. However, patients do not consistently utilize the nearest or most appropriate PHC facility despite the investments by member states to expand PHC infrastructure and services, particularly in ~~low- and middle-income countries~~LMICs like Ghana. Rather, their healthcare-seeking behavior reflects deliberate preferences for specific clinic or hospital types, often bypassing proximate facilities in favor of more distant ones [7, 10]. In Ghana, the number of outpatient consultations in PHC has not increased significantly, and patients still seek medical care at secondary or tertiary hospitals rather than PHC institutions as their first point of contact, leading to excessive demand and a burden on secondary and tertiary hospitals, while underutilizing PHC institutions [11]. For instance, In the Upper West Region of Ghana, a study by Farhan and Aryeetey [12] found a 77.8% utilization rate of secondary and tertiary healthcare compared to 22.2% utilization rate for PHC, suggesting a considerable wastage of high-cost medical resources in the secondary and tertiary hospitals. ~~And~~ T~~t~~his can easily result in prolonged waiting time for patients and excessive workloads for doctors and other healthcare providers. Research asserts that this preference or health-seeking behavior is influenced by an intricate interplay of various factors [7].

Studies demonstrate that patients' choice of a healthcare facility type is invariably influenced by their perceived ~~quality of care~~quality-of-care [13, 14]. In Kenya, a survey reported that perceived quality in terms of perceived staff competence and shorter waiting times significantly influenced preferences for private clinics over public hospitals [15]. Also, Olorunfemi et al. [16] in Nigeria reveal that patients often chose faith-based hospitals due to perceived ~~quality of care~~quality-of-care in terms of better interpersonal treatment, despite higher costs. Similarly, Atinga et al. [17] in Ghana found that perceived healthcare quality pushes patients toward private providers. This implies that, irrespective of it being publicly or privately financed, the quality of healthcare services impacts patients' choices for facility type. Studies also reveal that accessibility factors impact patients' choice of a healthcare facility type. For instance, Verma and Dash [18] found that 68% of respondents preferred local clinics over distant hospitals due to travel constraints. Syed et al. [19] reported that urban populations favored specialized facilities, whereas rural residents relied on primary care centers due to shorter travel distances. Unaffordable costs, low income levels, and out-of-pocket expenses influence the use of public facilities over private facilities [20, 21]. Poor road networks [22, 23], travel time and distance [24] were also found to influence the choice of healthcare among household members. Otchere et al. [25] on accessibility and utilization of health-care services among rural–urban migrants in Ghana, revealed that rural Ghanaians prioritized proximity, with 60% opting for Community-Based Health Planning. These pieces of evidence demonstrate that geographical accessibility, financial barriers, and infrastructural limitations influence patients' choices for healthcare facility type.

Despite the efforts of existing studies [18–25] highlighting factors that impact individual choice of healthcare facility type, very few have disaggregated analysis at the level of PHC facility type, leaving unclear why patients distinguish between and selectively prefer specific categories of PHC providers within the same geographic and policy environment. Also, the overwhelming majority of facility preference studies have been conducted in urban settings [7, 26], rendering their findings poorly transferable to rural and semi-urban municipal contexts such as Bolgatanga, where informal health norms, community trust structures, and provider-patient relational dynamics operate differently. Again, with the need to promote the utilization of PHC services in Bolgatanga Municipality due to a recent report on inconsistent utilization patterns across different PHC facilities, there is the need to assess the existing factors that impact patients' choices of ~~primary healthcare~~ PHC facility type for local context evidence to support national decision-making. To achieve this aim, the following research questions were addressed.

1. What is the role of healthcare service quality in shaping individual preferences for healthcare facility type in Bolgatanga Municipality in the Upper East Region of Ghana?

2. What is the effect of accessibility factors on individual preferences for healthcare facility type in Bolgatanga Municipality in the Upper East Region of Ghana?

Methods and Materials

Study design and population

The study employed a cross-sectional study design. A cross-sectional design is appropriate for this study because it enabled the simultaneous collection of data on ~~quality of care~~quality-of-care, accessibility factors, and healthcare facility preferences from a defined population at a single point in time, making it both time- and cost-efficient for examining associations across a large sample in the Bolgatanga Municipality [27].~~ Since the study aims to estimate the prevalence of preferences and identify associated factors, rather than establish causal temporality and as preferences for primary healthcare facility types are relatively stable attributes that do not require longitudinal tracking, a cross-sectional approach was methodologically well-suited~~. This study included adults~~,~~ 18 years and above, who live in Bolgatanga and accepted the consent to participate. This study excluded individuals less than 18 years and adults who are seriously ill or mentally unfit to participate in the research and patients who did not give consent.~~denied their consent to participate~~.

Profile of study area

This study was confined to the settings of Bolgatanga. Bolgatanga Municipal is in the center of the Upper East Region and is the regional capital. It has a total land area of 729 sq. km and is bordered to the north by the Bongo District, to the south and east by Talensi, and to the west by the Kassena-Nankana Municipality. The municipality was established by LI 1797 (2004) [28]. As of 2021, the town had~~s~~ a population of about 142,509 people, with females (74,659) representing 52.4% and males (67,850) representing 47.6 %. The municipality ~~has been~~is divided into nine (9) sub-municipalities, namely Bolga Central, Bolga North, Bolga South, Kalbeo, Sherigu, Sumbrungu East, Sumbrungu West, Plaza and Zaare. The capital of the municipality is Bolgatanga, which is cosmopolitan. The major tribe is Frafra, with significant tribes from within the Upper East Region, as well as other neighbouring regions in the country. The climate is classified as tropical and has two distinct ~~seasons – the~~seasons, the rainy season that runs from May to October and a long dry season that stretches from October to April, with hardly any rain. Mean annual rainfall is 950 mm, while the maximum temperature is 45°C in March and April with a minimum of 12°C in December~~. The people are mainly peasant farmers who grow few vegetables, such as tomatoes, in commercial quantities during the tomato farming season. A handful of women also deal in basketry, and this accounts for as much as 57% of the labor force, trade and commerce 19%, manufacturing (mainly handicrafts) 11.92%, community/social services~~

(7.4%), and others like mining, construction, and utility services [28]. The selection of this study area is influenced by inconsistent utilization patterns across different PHC facilities in the municipality observed by the health workers.

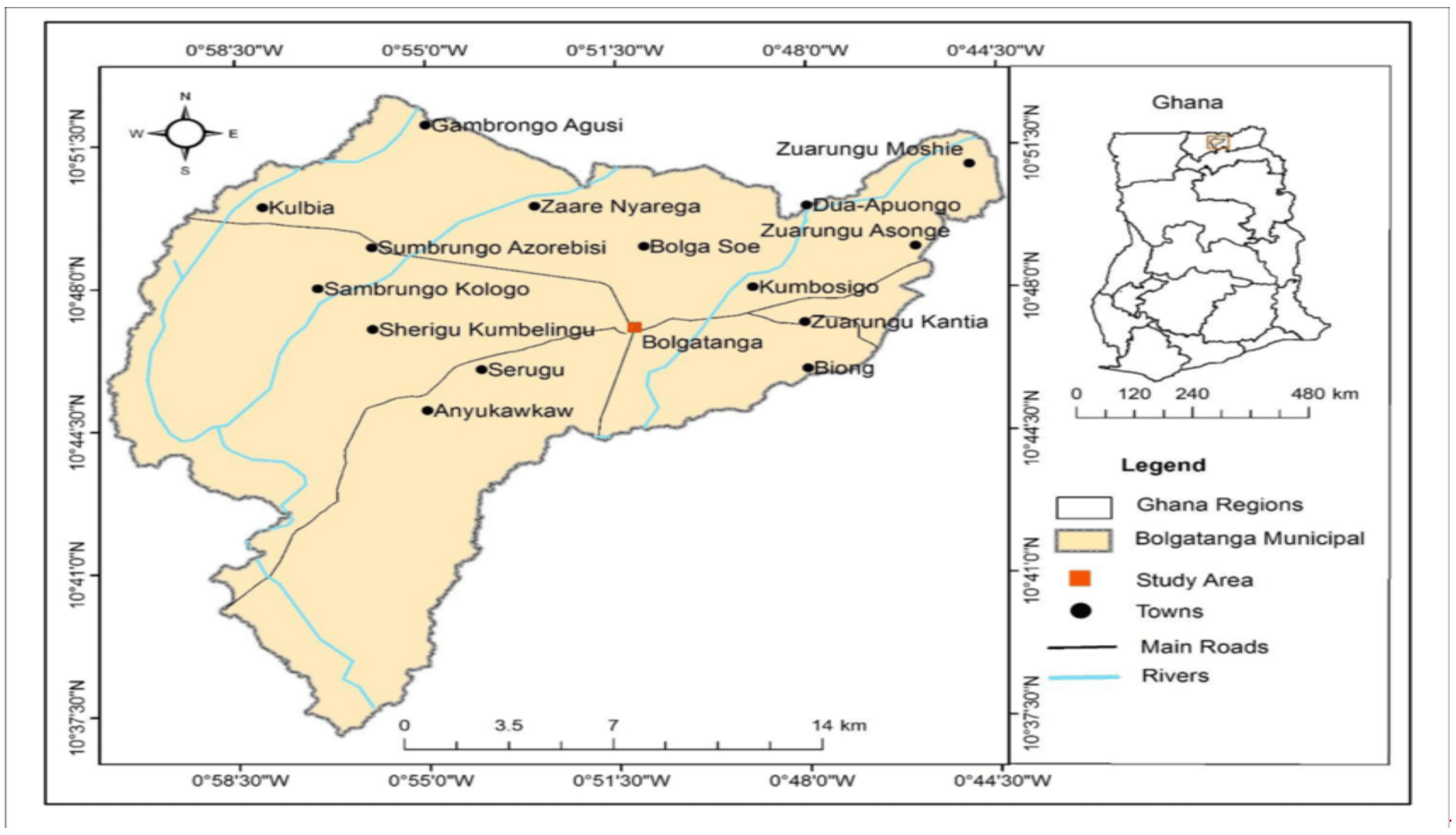

Figure 2: Map of Bolgatanga Municipal (Source: [28])

Theoretical Model/Framework

This study employed Andersen's Behavioral Model of Health Services Use as the theoretical framework to explain the key factors that influence individual preferences for primary healthcare PHC facility type in Bolgatanga Municipality, Upper East Region, Ghana. The model was developed by Andersen and was based on a national quantitative survey that aimed to understand families' use of health services [29, 30]. The model focuses on three core factors to explain healthcare utilization: predisposing factors such as age and education; enabling factors such as income, hospital density; and need factors such as health status [29, 31].

In the context of this study, the model's enabling and predisposing domains directly inform both the research design and the selection of variables. Specifically, the accessibility factors examined, namely distance to facility, transportation options, and cost of service, are theoretically situated within Andersen's enabling domain, as they constitute the structural and economic conditions that either facilitate or impede an individual's capacity to reach and engage with a preferred PHC facility type [29, 31]. Concurrently, the healthcare service quality dimensions encompassing waiting time, cleanliness, communication with provider, and drug availability align predominantly with Andersen's predisposing and enabling constructs, as they reflect individuals' evaluative

perceptions of the care environment that shape attitudinal dispositions and reinforce or redirect facility preferences over repeated health-seeking episodes. The model's conceptual logic further justifies the adoption of a cross-sectional research design, as Andersen's framework is fundamentally a snapshot model of utilization behavior. It theorizes the simultaneous interplay of predisposing, enabling, and need conditions at a given point in time rather than tracing longitudinal behavioral trajectories, making a cross-sectional survey the most epistemologically coherent design for capturing and measuring the concurrent influence of these variable domains on facility-type preference. In the study preferred facility types included in the study are District hospital, Private clinic/hospital/Faith based, Government health centers or polyclinic and CHPS compound. District hospitals were included because, within the Ghana Health Service referral framework, they often serve as the first point of contact for many communities and provide a substantial proportion of primary healthcare services, particularly in areas like Bolgatanga, where lower-level facilities have limited capacity. Respondents were asked about their preferred facility for seeking primary healthcare services rather than hypothetical choices. Analytically, the model's tripartite structure guides the organization of variables into theoretically meaningful blocks, informing a multivariate analytical approach, most appropriately multinomial logistic regression, wherein accessibility factors and service quality dimensions are examined as independent predictors of facility-type preference, consistent with Andersen's proposition that utilization outcomes are the product of the additive and interactive influence of these determinant domains. Consequently, far from being a passive theoretical reference, Andersen's Behavioral Model of Health Services actively structures every methodological decision in this study, from what is measured and why to how variables are categorized and the manner in which their relationships with PHC facility-type preferences are estimated and interpreted, thereby ensuring that the study's empirical inquiry is theoretically grounded, internally coherent, and meaningfully positioned within the broader canon of health services utilization research [29, 30].

Sampling methods

The optimal sample size was determined using the Yamane (1973) formula which assumes simple random sampling [32], with population (N = 142,509), and margin of error (e = 0.05). A total of 398 accurate and complete questionnaires were obtained, which matched the actual sample size estimated without the nonresponse rate. For this study, the researcher used a ~~two~~three-stage cluster sampling method. The absence of design effect adjustment is due to the fact that, the primary objective of the study was to obtain population-level estimates and examine associations among variables rather than produce highly precise cluster-level estimates. Though the use of random selection at each sampling stage helped to reduce selection bias, the use of multistage cluster

sampling may have introduced intra-cluster correlation, which could result in larger standard errors and slightly reduced statistical precision.

In the first stage, First, the study area was divided into clusters based on the nine (9) sub-municipalities, namely Bolga Central, Bolga North, Bolga South, Kalbeo, Sherigu, Sumbrungu East, Sumbrungu West, Plaza, and Zaare. From these, 5 clusters (Bolga Central, Bolga South, Sherigu, Sumbrungu East, and Plaza) were randomly selected. In the second stage, next stage the researchers randomly selected compounds from each selected cluster by assigning codes with the aid of random number generator software. The researcher then listed all the households in each selected compound. A household here means a father, mother, and children or relatives under one roof, so a compound may have more than one household. Sub-municipalities may differ substantially in socioeconomic status, education, ethnicity, and urbanization, and households located in compounds of different sizes may have unequal probabilities of selection. To address this, the number of compounds selected per sub-municipality was proportional to the total number of compounds identified in that cluster, thereby minimizing selection bias arising from compound size heterogeneity. From the 250 selected compound households, the researcher selected not more than two adults from each household at random to take part in the study at the third stage. Here, and with the aid of random number generator software, the participants were assigned a code and surveyed. This method made the process fair and ensured that every household has an equal chance of being included.

Data collection techniques

Data for this study was collected using researcher-administered questionnaires. A novel questionnaire was developed by the researchers specifically for the purposes of this study and uploaded under the supplementary file. These were administered through face-to-face interviews, which helped to ensure accurate responses from participants who had difficulties reading or understanding the questions independently. The questionnaire developed for this study underwent a rigorous content validity process, whereby five experts in health research from the department of Health Policy, Management, and Economics, KNUST independently evaluated each item for relevance, clarity, and representativeness using a Content Validity Index (CVI), yielding an item-level CVI (I-CVI) of ≥0.80 and a scale-level CVI (S-CVI) of 0.92, consistent with established thresholds for acceptable content validity. To further establish instrument reliability, a pilot test was conducted with 20 respondents drawn from Tafo community, Kumasi. The pilot exercise focused on instrument refinement rather than context-specific behavioural measurement; therefore, emphasis was placed on linguistic clarity and interpretability of items rather than contextual equivalence of responses. However, we recognize that Kumasi is socio-culturally and

geographically different from the study area (Bolgatanga), and this may limit the extent to which contextual nuances were captured during pretesting. Also, internal consistency was measured using a Cronbach's alpha, where all construct yielded a coefficient ≥ 0.70. This confirms satisfactory internal consistency of the self-reported items. Construct validity was additionally supported via expert panel feedback, which was used iteratively to refine item wording for interpretability and contextual appropriateness prior to final administration.

~~Content validity was established through expert review by five experts in health research from the department of Health Policy, Management, and Economics, KNUST, and piloted among 20 groups of participants from the Tafo community, Kumasi. This helped to fine-tune the questions and remove confusing ones.~~

Data analysis

The data ~~were~~ was entered and analyzed using Stata Version 17.0 and SPSS Version 22.0. SPSS Version 22 was used for descriptive statistics and multinomial logistic regression, while Stata Version 17 was used for additional model diagnostics including VIF estimation. Descriptive data were presented using tables, frequencies, percentages, and charts where necessary. Initial bivariate analysis was conducted to examine crude associations between healthcare facility preference and all independent variables, including healthcare service and accessibility factors. Specifically, Chi-square tests were used for categorical variables to identify statistically significant relationships at the bivariate level ($p < 0.05$), consistent with standard modelling procedures in health services research. Variables that demonstrated theoretical relevance or significant crude associations were subsequently considered for inclusion in the multivariable model to control for potential confounding effects and improve the robustness of parameter estimates. Multinomial logistic regression was also used to determine the role of healthcare service quality and accessibility factors in shaping individual preferences for healthcare facility type. The model diagnostic tests revealed a Cox & Snell Pseudo R^2 value of 0.341 for perceived-quality-of-care and 0.431 for accessibility factors, indicating that the independent variables collectively explain approximately 34.1% and 43.1% of the variation in PHC facility-type preference and the two models respectively. The Variance Inflation Factor (VIF) values ranging from 1.198 to 1.523 and corresponding tolerance values between 0.657 and 0.835 for all the models are all well within acceptable thresholds, confirming the absence of multicollinearity among the predictor variables, thereby affirming the statistical integrity of the regression estimates. Statistical significance for all testing was set at 0.05.

Ethics approval

Ethical approval for this study was obtained from the Committee on Human Research, Publications, and Ethics (CHRPE), KNUST, in relation to the Ghana Data Protection Act, 2012 (Act 843) national guidelines, with the reference number CHRPE/AP/011/26. The study was conducted in accordance with the ethical principles outlined in the Declaration of Helsinki. All participants were informed about the purpose of the study, assured of confidentiality, and provided written informed consent prior to participation. All participants were subject to freely given, informed consent to participate in the study. Participation was voluntary, and respondents could withdraw at any time without consequence.

Results

Socio-demographic characteristics of the respondents

Table 1 provides a summary of the socio-demographic characteristics of the 398 respondents. The results show that the largest age group was 31–45 years (43.0%), while the smallest was 61–83 years (6.3%). Regarding education, most respondents had completed senior high school (32.2%), whereas the ~~fewest~~ least had no formal education (18.8%). In terms of marital status, the majority of the respondents were married (53.8%), and only a small proportion were cohabiting (1.0%). Christianity was the dominant religion (59.5%), while those with no religion formed the smallest group (2.0%). By ~~the~~ occupation, traders constituted the largest category (25.9%), whereas those in the other category (retired, security guard, and hairdresser) accounted for the smallest share (3.8%). Income distribution showed that most respondents earned GHC 200–1500 (41.0%), whereas only 4.0% earned GHC 5001–7000. Finally, NHIS status revealed that most respondents were registered and active members (74.9%), while the ~~fewest~~ least were not registered (3.8%).

Table 1: Socio-demographic characteristics of the respondents

Variable		Frequency (N=398)	Percentage (%)
Age	18–30yrs	139	34.9
	31–45yrs	171	43.0
	46–60yrs	63	15.8
	61–83yrs	25	6.3
Highest level of education	No formal education	75	18.8
	Basic (Up to JHS)	83	20.9
	Senior High School (SHS)	128	32.2
	Tertiary (Diploma, HND, Degree)	112	28.1
Marital status	Single	155	38.9
	Married	214	53.8
	Cohabiting	4	1.0
	Divorced/Separated	13	3.3
	Widowed	12	3.0
Religion	Christianity	237	59.5
	Islam	103	25.9
	Traditional Religion	50	12.6
	No religion	8	2.0
Occupation	Unemployed	83	20.9
	Farmer	45	11.3
	Trader	103	25.9
	Civil/Public servant	75	18.8
	Private sector worker	22	5.5
	Artisan (e.g., carpenter, mason)	22	5.5
	Student	33	8.3
	Other	15	3.8
Income	GHC200-1500	163	41.0
	GHC1501-3000	83	20.9
	GHC3001-5000	136	34.2
	GHC5001-7000	16	4.0
NHIS Status	Not Registered	15	3.8
	Registered but not active	85	21.4
	Registered and active membership	298	74.9

Formatted Table

327

The role of healthcare service quality in shaping individual preferences for ~~primary healthcare~~PHC facility type

Table 2 presents a descriptive analysis of the ~~quality of care~~quality-of-care, preferred healthcare facility type, and factors influencing respondents' healthcare choices. Among the 398 respondents, most rated the ~~quality of care~~quality-of-care as good (45.7%), whereas very few rated it as poor (0.5%) or excellent (1.0%). For service use, the majority preferred facilities for routine check-ups (62.8%), whereas the smallest proportion visited for specialized treatment (9.3%). District hospitals were the most preferred ~~primary healthcare~~PHC facilities (43.2%), while private clinics, faith-based facilities, and CHPS compounds were the least preferred (12.8% each). Across the influencing factors, most respondents consistently rated ~~quality of care~~quality-of-care (76.4%), waiting time (77.1%), staff attitude (75.1%), availability of follow-up care (70.1%), specialized services (68.8%), cost (57.0%), medicine availability (67.3%), and clean environment (70.9%) as important, with very few considering these factors not important. Among influence-related variables, personal recommendation (77.4%), facility reputation (74.6%), insurance coverage (75.9%), and accessibility (76.1%) were rated as highly influential, whereas online reviews were rated moderately influential (59.0%), making them the least influential factor overall.

Tables 3 and 4 present a multinomial logistic regression result examining the association between ~~quality of care~~quality-of-care and the type of healthcare facility preferred, obtained from SPSS V.22 software. The model was statistically significant ($\chi^2(36) = 208.71$, $p < .001$), indicating that the predictors jointly explained a meaningful proportion of variance in facility choice. The multinomial logistic regression results indicate that ~~quality of care~~quality-of-care significantly influences respondents' preferences for ~~primary healthcare~~PHC type after adjusting for age, education, NHIS status, and cost.

For private clinics/faith-based facilities, respondents who rated ~~quality of care~~quality-of-care as very important compared to not important are 74% more likely to prefer for private clinics/faith-based facilities compared to CHPS compounds (aOR = 1.74, 95% CI: 1.02–9.73, p = 0.026).

Among those who considered ~~quality of care~~quality-of-care important rather than not important were 56% higher (aOR = 1.56, 95% CI: 1.05–2.91, p = 0.008). Both respondents who rated ~~quality of care~~quality-of-care as important (aOR = 2.89, 95% CI: 1.02–9.78, p = 0.033) and

very important (aOR = 1.25, 95% CI: 1.01–3.61, p = 0.004) had higher odds of preferring district hospitals over CHPS compounds.

Table 2: Descriptive analysis of ~~quality of care~~quality-of-care and preferred ~~primary healthcare~~PHC facility type

Variable		Frequency(f)	Percentage
~~Quality of care~~Quality-of-care	Poor	2	0.5
	Good	182	45.7
	Fair	128	32.2
	Very Good	82	20.6
	Excellent	4	1.0
Healthcare facility preferred for	Routine check-ups	250	62.8
	Emergency care	111	27.9
	Specialized treatment	37	9.3
Preferred ~~primary healthcare~~PHC facility types	District hospital	172	43.2
	Private clinic/hospital/Faith based	51	12.8
	Government health centers or polyclinic	124	31.2
	CHPS compound	51	12.8
**TOTAL (n)**		**398**	**100%**

Table 3: Descriptive analysis of factors influencing individual preferences for healthcare facility type

Factor	Not Important	Neutral	Important	Very Impo
~~Quality of care~~Quality-of-care	0 (0.0%)	13 (3.3%)	304 (76.4%)	81 (20.4%)
Waiting time	0 (0.0%)	9 (2.3%)	306 (77.1%)	82 (20.7%)
Staff attitude & communication	0 (0.0%)	17 (4.3%)	299 (75.1%)	82 (20.6%)
Availability of follow-up care	19 (4.8%)	27 (6.8%)	279 (70.1%)	73 (18.3%)
Availability of specialized services	13 (3.3%)	33 (8.3%)	274 (68.8%)	78 (19.6%)
Cost	0 (0.0%)	23 (5.8%)	225 (57.0%)	147 (37.2%)
Availability of medicines	0 (0.0%)	12 (3.0%)	268 (67.3%)	118 (29.6%)
Clean environment	2 (0.5%)	22 (5.5%)	282 (70.9%)	92 (23.1%)
	**Not Influential**	**Neutral**	**Influential**	**Very Influential**
Personal recommendations	4 (1.0%)	36 (9.0%)	308 (77.4%)	50 (12.6%)
Online reviews	12 (3.0%)	107 (26.9%)	235 (59.0%)	44 (11.1%)
Facility reputation	6 (1.5%)	39 (9.8%)	297 (74.6%)	56 (14.1%)
Insurance coverage	0 (0.0%)	14 (3.5%)	302 (75.9%)	82 (20.6%)
Accessibility and location	0 (0.0%)	16(4.1%)	300(76.1%)	78(19.8%)
		**n=398(100%)**		

Table 4: Multinomial logistic regression of the association between ~~quality of care~~quality-of-care and preferred ~~primary healthcare~~PHC facility type

Variable		Odds (95% CI)	p-value
**Private clinic / Faith-based vs CHPS**			
~~Quality of care~~Quality-of-care	Not Important	~~1~~ (~~base~~Ref)	
	Important	0.95 (0.240, 5.68)	0.994
	Very Important	1.74 (1.02, 9.73)	0.026
Age	18–30 yrs	~~1 (base)~~Ref	
	31–45 yrs	3.27 (1.17, 9.08)	0.023
	46–60 yrs	2.87 (0.51, 16.05)	0.23
	61–83 yrs	6.41×10^{7} (−)	0.991
Education	No formal education	~~1 (base)~~Ref	
	Basic	0.90 (0.25, 3.29)	0.872
	SHS	2.41 (0.69, 8.45)	0.166
	Tertiary	4.83 (2.90, 9.67)	0.027
NHIS status	Not registered	~~1 (base)~~Ref	
	Registered, not active	3.05 (0.34, 27.70)	0.322
	Registered, active	0.76.07 (0.13, 0.97)	0.003
Cost	Not Important	~~1 (base)~~Ref	
	Important	0.68 (0.09, 5.36)	0.723
	Very Important	0.17 (0.01, 0.73)	0.029
**Government health centre / Polyclinic vs CHPS**			
~~Quality of care~~Quality-of-care	Not Important	~~1 (base)~~Ref	
	Important	1.56 (1.05, 2.91)	0.008
	Very Important	0.46 (0.13, 0.89)	0.992
Age	18–30 yrs	~~1 (base)~~Ref	
	31–45 yrs	3.83 (1.64, 8.95)	0.002
	46–60 yrs	3.33 (0.88, 12.60)	0.077
	61–83 yrs	3.48×10^{6} (−)	0.992
Education	No formal education	~~1 (base)~~Ref	
	Basic	0.61 (0.22, 1.69)	0.345
	SHS	1.64 (0.60, 4.49)	0.334
	Tertiary	3.76 (0.88, 16.02)	0.074
NHIS status	Not registered	~~1 (base)~~Ref	
	Registered, not active	1.90 (0.29, 10.41)	0.505
	Registered, active	3.26 (0.57, 16.96)	0.183
Cost	Neutral	~~1 (base)~~Ref	
	Important	5.45 (1.55, 10.83)	0.025
	Very Important	3.02 (0.76, 29.65)	0.341

Formatted Table

Table 4 Continued: Multinomial logistic regression of the association between ~~quality of care~~quality-of-care and preferred ~~primary healthcare~~PHC facility type

Variable	Category	Odds (95% CI)	p-value
**District hospital vs CHPS**			
~~Quality of care~~Quality-of-care	Not Important	~~1 (base)~~Ref	
	Important	2.89(1.02, 9.78)	0.033
	Very Important	1.25 (1.01, 3.61)	0.004
Age	18–30 yrs	~~1 (base)~~Ref	
	31–45 yrs	8.87 (3.63, 21.62)	≤ 0.001~~0.000~~
	46–60 yrs	14.60 (3.79, 56.07)	≤ 0.001~~0.000~~
	61–83 yrs	0.0014(0.00, 21.09)	0.991
Education	No formal education	Ref~~Ref~~	
	Basic	1.21 (0.40, 4.37)	0.733
	SHS	3.03 (1.01, 9.09)	0.047
	Tertiary	14.08 (3.19, 62.02)	≤ 0.001~~0.000~~
NHIS status	Not registered	Ref~~1 (base)~~	
	Registered, not active	2.96 (0.45, 19.56)	0.259
	Registered, active	2.53 (0.45, 14.19)	0.295
Cost	Not Important	Ref~~1 (base)~~	
	Important	1.33 (0.07, 9.63)	0.779
	Very Important	0.61 (0.09, 0.98)	0.023

Formatted Table

Effect of accessibility factors on individual preferences for healthcare facility type

Table 5 provides a descriptive analysis of accessibility factors and barriers to healthcare services among the respondents. Among the 398 respondents, the majority had used healthcare services in the past six months (68.1%), whereas a smaller proportion had not (31.9%). Among the 271 individuals who had accessed care, the majority relied on both public and private facilities (57.6%), with fewer depending solely on public or private facilities (10.7%). Location played a major role in healthcare decisions, with most respondents (74.9%) indicating that it was influential, while only a small fraction found it not influential (1.0%). Willingness to travel varied by service type: most respondents were willing to travel further for specialized care (82.4%) and emergency care (60.8%), but willingness dropped for routine care, where slightly more than half (51.3%) were unwilling. Regarding barriers, most respondents reported no

barriers (69.3%). Among the 122 who experienced challenges, cost emerged as the most common barrier (80.3%), followed by long waiting times (57.4%) and distance (32%).

Tables 6 and 7 present the results of a multinomial logistic regression examining the influence of accessibility factors on the type of ~~primary healthcare~~PHC facility preferred from SPSS V.22 software. The model was statistically significant: $\chi^2(43) = 109.66$, $p < .001$ (indicating that distance, transportation options, waiting time, appointment availability, and cost jointly explained a meaningful proportion of variance in facility choice). Specifically, respondents who rated distance and location as very important rather than not important were 242% more likely to choose private clinics/faith-based facilities rather than CHPS compounds (aOR = 3.42, 95% CI: 1.78–6.84, p = 0.001). Those who considered transportation options as very important compared to not important were 301% more likely to select private clinics/faith-based facilities rather than CHPS compounds (aOR = 4.01, 95% CI: 2.10–7.83, p < 0.001). Respondents who rated waiting time as very important compared to not important are 21% more likely to choose private clinics/faith-based facilities compared to CHPS compounds (aOR = 1.21, 95% CI: 1.10–2.47, p < 0.001), while those who considered appointment availability as very important are 131% more likely to prefer these facilities compared to CHPS compounds (aOR = 2.31, 95% CI: 1.14–5.61, p = 0.001). Conversely, respondents who rated cost of care as very important are 12% less likely to choose private clinics/faith-based facilities compared to CHPS compounds (aOR = 0.88, 95% CI: 0.02–0.95, p < 0.001).

For government health centres or polyclinics, respondents who rated distance and location as very important are 134% more likely to select these facilities compared to CHPS compounds (aOR = 2.34, 95% CI: 1.24–4.56, p = 0.009), and those who considered transportation options as very important are 248% more likely to prefer them (aOR = 3.48, 95% CI: 1.82–6.68, p < 0.001). In contrast, respondents who rated waiting time as very important are 71% less likely to choose government facilities (aOR = 0.29, 95% CI: 0.14–0.55, p = 0.001), and those who considered appointment availability as very important are 66% less likely to select these facilities (aOR = 0.34, 95% CI: 0.17–0.66, p = 0.002). Respondents who rated the cost of transportation as very important are 171% more likely to prefer government health centres or polyclinics (aOR = 2.71, 95% CI: 1.40–5.37, p = 0.003). Regarding district hospitals, respondents who rated distance and location as very important are 355% more likely to choose district hospitals compared to CHPS compounds (aOR = 4.55, 95% CI: 2.09–8.91, p = 0.510), and those who considered transportation options as very important are 498% more likely to

select them (aOR = 5.98, 95% CI: 0.94–11.85, p = 0.700). Conversely, respondents who rated waiting time as very important are 83% less likely to choose district hospitals (aOR = 0.17, 95% CI: 0.08–0.33, p < 0.001), while those who considered appointment availability as very important are 68% less likely to select these facilities (aOR = 0.32, 95% CI: 0.16–0.62, p = 0.001). Respondents who rated cost of care as very important are 318% more likely to prefer district hospitals (aOR = 4.18, 95% CI: 2.11–8.21, p < 0.001).

Table 5: Descriptive analysis of accessibility factors and barriers to accessing healthcare services

**Item**		**Frequency (f)**	**Percentage (%)**
Have you used healthcare services in the past 6 months?	Yes	271	68.1
	No	127	31.9
**TOTAL**		**398**	**100%**
If yes, what type of healthcare facility do you typically use?			
	Public	86	31.7
	Private	29	10.7
	Both private and pubic	156	57.6
**TOTAL**		**271**	**100%**
How does the location of a healthcare facility influence your decision to use it?	Not Influential at All	25	6.3
	Not Influential	4	1.0
	Neutral	41	10.3
	Influential	298	74.9
	Very Influential	30	7.5
Would you be willing to travel further for Specialized care	No	70	17.6
	Yes	328	82.4
Would you be willing to travel further for Emergency care	No	156	39.2
	Yes	242	60.8
Would you be willing to travel further for Routine care	No	204	51.3
	Yes	194	48.7
Have you experienced any barriers to accessing healthcare services?	Yes	122	30.7
	No	276	69.3
**TOTAL**		**398**	**100%**
If yes what were the barriers (Multiple responses) a. transportation issues	No	112	91.8
	Yes	10	8.2
b. Long waiting times	No	52	42.6
	Yes	70	57.4
c. Cost	No	24	19.7
	Yes	98	80.3
d. Distance	No	83	68.0
	Yes	39	32.
e. Other (No drugs, community conflict)	No	118	96.7
	Yes	4	3.3
**TOTAL**		**610**	**100%**

Formatted Table

Table 6~~:~~: Multinomial logistic regression of accessibility factors influencing preferred ~~primary healthcare~~PHC facility type

Variable	Odds (95% CI)	P-value	
**Private clinic / Faith-based vs CHPS**			
Distance & Location	Not Important	Ref~~1 (base)~~	
	Important	1.88 (1.10, 3.57)	0.042
	Very Important	3.42 (1.78, 6.84)	0.001
Transportation Options	Not Important	Ref~~1 (base)~~	
	Important	2.15 (1.24, 4.02)	0.009
	Very Important	4.01 (2.10, 7.83)	< 0.001
Waiting Time	Not Important	Ref~~1 (base)~~	
	Important	1.54 (1.09, 2.97)	0.021
	Very Important	1.21 (1.10, 2.47)	< 0.001
Appointment Availability	Not Important	Ref~~1 (base)~~	
	Important	1.48 (0.26, 0.91)	0.024
	Very Important	2.31 (1.14, 5.61)	0.001
Cost of care	Not Important	Ref~~1 (base)~~	
	Important	0.74 (0.11, 0.99)	0.028
	Very Important	0.88 (0.02, 0.95)	< 0.001
**Government Health Centre / Polyclinic vs CHPS**			
Distance & Location	Not Important	Ref~~1 (base)~~	
	Important	1.72 (1.04, 3.14)	0.049
	Very Important	2.34 (1.24, 4.56)	0.009
Transportation Options	Not Important	Ref~~1 (base)~~	
	Important	2.28 (1.29, 4.26)	0.006
	Very Important	3.48 (1.82, 6.68)	< 0.001
Waiting Time	Not Important	Ref~~1 (base)~~	
	Important	0.47 (0.26, 0.82)	0.008
	Very Important	0.29 (0.14, 0.55)	0.001
Appointment Availability	Not Important	Ref~~1 (base)~~	
	Important	0.49 (0.27, 0.91)	0.024
	Very Important	0.34 (0.17, 0.66)	0.002
Cost of Transportation	Not Important	Ref~~1 (base)~~	
	Important	1.62 (0.95, 3.21)	0.071
	Very Important	2.71 (1.40, 5.37)	0.003

Formatted Table

Table 7: Multinomial logistic regression of accessibility factors influencing preferred ~~primary healthcare~~PHC facility type cont'd

Variable		Odds (95% CI)	P-value
**District Hospital vs CHPS**			
Distance & Location	Not Important	Ref~~1 (base)~~	
	Important	0.35 (0.27, 0.79)	0.008
	Very Important	4.55 (2029, 8.91)	0.510
Transportation Options	Not Important	Ref~~1 (base)~~	
	Important	0.68 (0.02, 0.95)	0.002
	Very Important	5.98 (0.94, 11.85)	0.700
Waiting Time	Not Important	Ref~~1 (base)~~	
	Important	0.39 (0.21, 0.70)	0.002
	Very Important	0.17 (0.08, 0.33)	< 0.001
Appointment Availability	Not Important	Ref~~1 (base)~~	
	Important	0.50 (0.27, 0.94)	0.032
	Very Important	0.32 (0.16, 0.62)	0.001
Cost of care	Not Important	Ref~~1 (base)~~	
	Important	2.20 (1.13, 4.28)	0.020
	Very Important	4.18 (2.11, 8.21)	< 0.001

Formatted Table

Discussions

~~**The role of healthcare service quality in shaping individual preferences for healthcare facility type in Bolgatanga Municipality**~~

Perceived ~~quality of care~~quality-of-care emerged as a determinant of individuals' preference for ~~primary healthcare~~PHC facility type in the Bolgatanga Municipality. In specific contexts, private or faith-based facilities, government health centers or polyclinics, and district hospitals rather than CHPS compounds were preferred by individuals who attributed greater importance to ~~quality of care~~quality-of-care. This shows that higher-level or alternative facilities that are perceived to offer better services are opted for due to higher valuation of quality. Also, individuals assess the standard of care they anticipate receiving and justify their facility choice with perceptions of competence, responsiveness, and service reliability beyond mere physical

access. This shows a growing demand for healthcare services that are not only accessible but also trustworthy, patient-centred, and effective. Through the assumptions of Andersen's Behavioral Model of Health Services Use, the findings reflect the interaction between predisposing, enabling, and need factors influencing an individual's preference for healthcare facility type [31]. Here, the perceived ~~quality of care~~quality-of-care operates as 1) a predisposing factor, through individual beliefs and expectations about healthcare. 2) a need-related factor, as patients seek facilities they believe can adequately address their health problems [29, 30]. This shows that the choice of hospitals and private facilities among those who highly value ~~quality of care~~quality-of-care suggests that when enabling resources are available, perceived quality becomes a decisive driver of utilization.

The current finding is supported by Bahrampour et al. [14] who report that service quality in terms of low waiting time, staff attitude, cleanliness, and follow-up care influenced patients' hospital preferences in Iran. In the same way, Gabrani et al. [33] supported that both public and private healthcare users in Albania prioritized ~~quality of care~~quality-of-care when choosing facilities. Gage et al. [34] showed that perceptions of provider responsiveness and service quality shaped utilization of ~~primary healthcare~~PHC facilities in Haiti. Studies in Africa by Mwami and Oleche [35] and Lokina and Musili [15] in supporting this current finding reported that perceived staff competence, shorter waiting times, and better interpersonal treatment drove patients toward private clinics in Kenya. Olorunfemi et al. [16] in similar context observed similar preferences for faith-based facilities in Nigeria due to better provider–patient interactions. On the other hand, Mhlanga and Hassan [36] found that cost considerations outweighed perceived quality among low-income households in South Africa. And this leads to continued preference for public facilities despite dissatisfaction. Also, the current findings showed that while the highest education level (Odds = 3.76, p = 0.074) was not significantly related to the choice of government health centre, NHIS status was also not significantly associated with both government health centers (Odds = 3.26) and district hospitals (Odds = 2.53). While these can be explained by structural and supply-side barriers such as facility availability, perceived quality, and proximity that may override individual-level socioeconomic characteristics in driving healthcare-seeking decisions in resource-constrained settings like Bolgatanga, the methodological limitations of this study are of concern. Methodologically, the cross-sectional design precludes any causal inference, as both exposure and outcome are measured simultaneously, making it impossible to determine whether

education or NHIS status precedes or follows facility preference. The use of multinomial logistic regression, while appropriate for polytomous outcomes, is sensitive to small cell sizes across facility categories, which may have inflated standard errors and reduced statistical power. This is a particularly acute concern, as the analysis is geographically confined to Bolgatanga municipality alone, limiting both sample diversity and the generalizability of findings to other urban, peri-urban, or rural contexts in the Upper East Region or Ghana broadly. Despite the demerits, these findings suggest the need to strengthen ~~quality of care~~quality-of-care at the primary level, particularly within CHPS compounds. Future studies should adopt larger, multi-site probability samples across diverse districts, incorporate longitudinal designs to track how NHIS enrollment dynamically influences facility choice over time, and consider multilevel modeling to disentangle individual-level from facility-level determinants.

~~**The association of accessibility factors with individual preferences for healthcare facility type in Bolgatanga Municipality**~~

In Bolgatanga, accessibility-related factors such as distance and location, transportation options, waiting time, appointment availability, and cost considerations were found to relate to individuals' preferences for ~~primary healthcare~~PHC facility type. This shows that multiple accessibility factors interact in individuals' daily lives in selecting facility type and that healthcare choices are not driven by a single access. From the study, individuals who valued proximity and transportation convenience preferred private clinics, faith-based facilities, and district hospitals. This may suggest that patients are willing to bypass nearby CHPS compounds when other facilities are perceived to be easier to reach or better connected by transport. Interestingly, dissatisfaction with congestion and delays commonly associated with higher-level public facilities were revealed in the study. Where participants who valued shorter waiting times and easier appointment availability were less likely to choose hospitals and government facilities. Also, the persistent relationship between financial barriers and an individual's healthcare behavior, particularly in low-resource settings, was revealed, where respondents who prioritized cost showed a lower preference for private facilities. In the context of this study, Andersen's Behavioral Model of Health Services Use, the findings relate to the central role of enabling factors in healthcare utilization. Thus, distance, transportation options, waiting time, appointment systems, and cost represent key enabling or constraining conditions that

determine whether individuals can translate their healthcare needs into actual service use [29,30].

Studies in India and the United States support the current findings by showing that distance, travel time, and transportation barriers significantly relate to facility choice, with rural populations often preferring nearby primary care facilities due to mobility constraints [18, 19] . Guimarães et al. [20] and Akkazieva and Zhao [21] also support the current findings, where they reported that financial accessibility often overrides geographical proximity, as low-income households avoid facilities perceived as costly even when they are close. Studies in Africa such as Mangundu et al. [22] and Oriyomi [23] support the current findings, documenting how long distances, poor road networks, and transport costs push rural households toward closer or informal healthcare options. In Ghana, Asante [24] and Otchere et al. [25] showed that proximity and travel time encourage the use of CHPS compounds, which support the current findings. Asare-Akuffo et al. [37] in supporting the current findings, found that individuals may travel longer distances when they perceive higher-level facilities as more capable of addressing their health needs. The identification of proximity and transportation convenience as drivers of preference for private clinics, faith-based facilities, and district hospitals particularly adds nuance to demand-side analyses in low-resource urban settings. Nonetheless, the study's cross-sectional, single-municipality design represents a significant methodological limitation. It captures a static snapshot of preferences without accounting for seasonal variation in access, such as during rainy seasons when transportation is disrupted, and the geographically bounded sample constrains transferability of findings beyond Bolgatanga's specific infrastructural and socioeconomic context. Furthermore, reliance on self-reported preferences may introduce social desirability bias, and the absence of objective facility-level data such as actual travel distances and verified waiting times limits triangulation of subjective perceptions against measurable access indicators. Despite the limitations, the study suggests the need for integrated interventions that address both physical and organizational access to care. That is, enhancing road networks, strengthening public transport systems, and decentralizing services could reduce the burden of distance and transportation barriers. Future research should employ mixed-methods longitudinal designs across multiple municipalities, integrating Geographic Information Systems (GIS) mapping of facility catchment areas with qualitative exploration of how accessibility trade-offs are negotiated across different household types and seasons.

Conclusions

The study aimed to identify the factors associated with individuals' preferences for ~~primary healthcare~~PHC facility type in the Bolgatanga Municipality, Upper East Region, Ghana. We found that ~~quality of care~~quality-of-care and accessibility-related factors such as distance and location, transportation options, waiting time, appointment availability, and cost considerations were associated with individuals' preferences for ~~primary healthcare~~PHC facility type. ~~In specific contexts, private or faith-based facilities, government health centers or polyclinics, and district hospitals rather than CHPS compounds were preferred by individuals who attributed greater importance to quality of care. Moreover, the highest education level (Odds = 3.76, p p= 0.074) was not significantly related to the choice of government health center; NHIS status was not significantly associated with either the government health center (Odds = 3.26, 0.183) or the district hospital (Odds = 2.53, p = 0.295).~~

In all, the study suggests that healthcare managers in Bolgatanga and the Ministry of Health should prioritize interventions aimed at improving ~~quality of care~~quality-of-care, such as improving interpersonal communication between health workers and patients, enhancing staff training, ensuring consistent availability of medications, and maintaining a hygienic and safe facility environment across all facility types. They should also provide effective and reliable transportation options, optimizing the location of new facilities, reducing waiting times through appointment management systems, and ensuring reasonable service fees. By addressing these quality-of-care gaps, public and CHPS facilities may become more attractive to residents who currently prefer private or faith-based facilities due to perceived superior service. Also, with these measures, barriers that currently drive patients toward higher-level facilities while maintaining affordability for low-income populations would reduce.

For methodological enhancement, future studies should adopt larger, multi-site probability samples across diverse districts, incorporate longitudinal designs to track how NHIS enrollment dynamically influences facility choice over time, and consider multilevel modeling to disentangle individual-level from facility-level determinants. Also, future research should employ mixed-methods longitudinal designs across multiple municipalities, integrating Geographic Information Systems (GIS) mapping of facility catchment areas with qualitative exploration of how accessibility trade-offs are negotiated across different household types and seasons. Also, linking survey data with routine health information systems would provide stronger behavioural validation.

Authors' Contributions

S.A., C.O., and K.A.M. conceptualized and designed the study. S.A. and L.A. collected the data under the supervision of K.A.M. and L.A., and CO analyzed and interpreted the data and wrote the draft manuscript. All authors read and approved the final manuscript.

Acknowledgement

Not Applicable

References

1. Zhang W, Ung COL, Lin G, Liu J, Li W, Hu H, et al. Factors contributing to patients' preferences for primary health care institutions in China: a qualitative study. Frontiers in public health, 8, 414 Aday. L A, & Andersen, R. 2020;9(3):208–20.

2. WHO. Key Facts: Primary health care [Internet]. 2025 [cited 2025 Jun 11]. Available from: https://www.who.int/news-room/fact-sheets/detail/primary-health-care

3. Ministry of Health G. Ghana health sector medium-term development plan 2022–2025. Ministry of Health; 2020.

4. Allen LN, Pettigrew LM, Exley J, Nugent R, Balabanova D, Villar-Uribe M, et al. The role of Primary Health Care, primary care and hospitals in advancing Universal Health Coverage. BMJ global health. 2023;8(12).

5. Shyaka A, Igihozo G, Tegli M, Ntiyaduhanye E, Ndizeye E, Umuhoza A, et al. Rwanda One health achievements and challenges: the final push toward policy implementation. One Health Cases. 2024;20240010.

6. Renggli S, Mayumana I, Mboya D, Charles C, Mshana C, Kessy F, et al. Towards improved health service quality in Tanzania: appropriateness of an electronic tool to assess quality of ~~primary healthcare~~Primary Health Care. BMC health services research. 2019;19:1–16.

7. Armah P, Kicha D. Primary health care in Ghana: the structure and functions in relation to preventing neglected tropical diseases. Archiv Euromed. 2020;10:12–7.

8. Ghana Health Service. Ministry of Health 2020 Annual Performance Report [Internet]. Gana: National Development Planning Commission; 2020 [cited 2025 Jun 11]. Available from: https://www.ndpc.gov.gh/media/Ministry_of_Health_APR_2020_XT2Z015.pdf

9. Kraef C, Juma P, Kallestrup P, Mucumbitsi J, Ramaiya K, Yonga G. The COVID-19 pandemic and non-communicable diseases—a wake-up call for primary health care system strengthening in sub-Saharan Africa. Journal of primary care & community health. 2020;11.

10. Asenso-Boadi F, Aikins M, Agyepong IA. Bypassing closer health services: A study of patients visiting a district hospital in Ghana. Global Health Action. 2016;9(1):31157. doi:10.3402/gha.v9.31157

11. Tuoyire DA, Baatiema L, Dwomoh D. Healthcare utilization in Ghana: Insights from the Ghana Living Standard Survey. PLOS ONE. 2017;19(6):e0306032. doi:10.1371/journal.pone.0306032

12. Farhan A, Aryeetey GC. Factors influencing utilization of healthcare services in Danku in the upper west region of Ghana: a mixed method study. J Glob Health Sci. 2024;6(2):e15. doi:10.35500/jghs.2024.6.e15

13. Senapati S, Panda RK. Enriching healthcare service quality by leveraging service experiences in Indian private hospitals: application of fuzzy-AHP approach. International Journal of Quality & Reliability Management. 2023;40(10):2301–25.

14. Bahrampour M, Bahrampour A, Amiresmaili M, Barouni M. Hospital service quality–patient preferences–a discrete choice experiment. International journal of health care quality assurance. 2018;31(7):676–83.

15. Lokina R, Musili W. Household's Choice of Healthcare Provider in Kenya. The African Review. 2021;48(1):239–62.

16. Olorunfemi O, Agbaje OC, Abiodun OO, Ayeni BA, Osunde RN. Understanding the Roles of Religion in Nursing Practice as a Prescriptive Instrument for High-Quality Faith-Based Healthcare. Amrita Journal of Medicine. 2024;20(4):144–8.

17. Atinga RA, Abekah-Nkrumah G, Domfeh KA. Managing healthcare quality in Ghana: a necessity of patient satisfaction. International journal of health care quality assurance. 2020;24(7):548–63.

18. Verma VR, Dash U. Geographical accessibility and spatial coverage modelling of public health care network in rural and remote India. PloS one. 2020;15(10):e0239326.

19. Syed ST, Gerber BS, Sharp LK. Traveling towards disease: transportation barriers to health care access. Journal of community health. 2023;38:976–93.

20. Guimarães T, Lucas K, Timms P. Understanding how low-income communities gain access to healthcare services: A qualitative study in São Paulo, Brazil. Journal of Transport & Health. 2019;15:100658.

21. Van Doorslaer E, O'Donnell O, Rannan-Eliya RP, Somanathan A, Adhikari SR, Akkazieva B, et al. Paying out-of-pocket for health care in Asia: Catastrophic and poverty impact. Rotterdam and IPS, Colombo: Erasmus University; 2021.

22. Mangundu M, Roets L, Janse van Rensberg E. Accessibility of healthcare in rural Zimbabwe: The perspective of nurses and healthcare users. African journal of primary health care & family medicine. 2020;12(1):e1–7. doi:10.4102/phcfm.v12i1.2245

23. Oriyomi K. Factors influencing choice of healthcare facilities utilisation by rural households in Ogun State, Nigeria. AGRICULTURA TROPICA ET SUBTROPICA. 2023;56.

24. Asante AG. Choice of Healthcare Services and Its Influencing Factors in the Sunyani Municipality [PhD Thesis]. University of Ghana; 2020.

25. Otchere G, Ankomah SE, Fusheini A, Kumah E, Agyei SK. Accessibility and utilization of health-care services among rural–urban migrants in Ghana: A scoping review. International Journal of Population Studies. 2024;10(4):1–17.

26. Saeed BII, Yawson AE, Nguah S, Agyei-Baffour P, Emmanuel N, Ayesu E. Effect of socio-economic factors in utilization of different healthcare services among older adult men and women in Ghana. BMC Health Services Research. 2016;16:1–9.

27. Cvetkovic-Vega A, Maguiña JL, Soto A, Lama-Valdivia J, Correa López LE. Cross-sectional studies. Revista de la Facultad de Medicina Humana. 2021;21(1):164–70.

28. Ghana Statistical Service. 2021 Population and housing census: National analytical report [Internet]. GSS; 2021. Available from: https://statsghana.gov.gh/

29. Andersen RM. National health surveys and the behavioral model of health services use. Med Care. 2008;46:647–53.

30. Andersen RA. Behavioral Model of Families' Use of Health Services. Chicago, Ill: Univ. of Chicago; 1968.

31. Lederle M, Tempes J, Bitzer EM. Application of Andersen's behavioural model of health services use: a scoping review with a focus on qualitative health services research. BMJ open. 2021;11(5):e045018.

32. Omair A. Sample size estimation and sampling techniques for selecting a representative sample. Journal of Health specialties. 2025;2(4):142.

33. Gabrani J, Schindler C, Wyss K. Factors associated with the utilisation of primary care services: a cross-sectional study in public and private facilities in Albania. BMJ. 2020;Open;10: e040398. doi:10.1136/bmjopen-2020-040398

34. Gage AD, Leslie HH, Bitton A, Jerome JG, Joseph JP, Thermidor R, et al. Gage Does quality influence utilization of primary health care? Evidence from Haiti Globalization and health. 2018;14:1–9.

35. Mwami MN, Oleche MO. Determinants of utilization of health care services in Kenya. International Journal of Academic Research in Business and Social Sciences. 2020;7(10):132–56.

36. Mhlanga D, Hassan A. An investigation of the factors influencing the choice of healthcare facility in South Africa. International Journal of Research in Business and Social Science. 2022;11(4):2147–4478.

37. Asare-Akuffo F, Twumasi-Boakye R, Appiah-Opoku S, Sobanjo JO. Spatial accessibility to hospital facilities: the case of Kumasi, Ghana. African Geographical Review. 2020;39(2):120–33.

Charles Obeng,
Department of Health Policy, Management and Economics,
Kwame Nkrumah University of Science and Technology (KNUST)
Kumasi, Ghana.
Email: obengcharles436@gmail.com
12 June 2026

The Editor-in-Chief
PLOS ONE

Re: Response to Reviewers – Revised Manuscript

Dear Editor,

We sincerely thank the Editor and all three Reviewers for their constructive and detailed feedback. We have carefully addressed each comment in the revised manuscript. Below, we provide point-by-point responses. Reviewer comments are presented in italics; our responses follow in regular text.

AUDITOR'S COMMENT

Comment:
Delete all subheadings from the Discussion section to ensure smoother flow.

Response:
All subheadings have been removed from the Discussion section. The content has been restructured into continuous prose to improve readability and flow.

REVIEWER #1

Comment:
Justify the selection of only quality of care, age, education, NHIS status, and cost in the multinomial logistic regression.

Response:
We appreciate this comment. Quality of care was the primary explanatory variable, directly aligned with the study's objective of examining its influence on facility preference. Age, education, NHIS status, and cost were included as control variables to adjust for potential confounding effects. These covariates were selected based on theoretical relevance and evidence from prior literature demonstrating their consistent influence on healthcare-seeking behaviour and facility utilisation decisions.

REVIEWER #2

General Comments

Comment 1: Conceptualization of Facility Preference

District hospitals are included as PHC facilities but often function as secondary-level referral facilities in Ghana. The authors should justify their inclusion and clarify whether questions concerned actual utilization or hypothetical preference, and how facility categories align with Ghana Health Service (GHS) classifications.

Response:

We acknowledge this concern. In the Ghanaian health system context, district hospitals serve dual roles, often functioning as the primary point of care in districts with limited health centre coverage. For this study, PHC facility types were defined based on the functional and accessible role they play in the community, consistent with how GHS operationalises first-contact care at the district level. Additionally, the questionnaire focused on respondents' preferences for healthcare facility use rather than referral utilization. This clarification has been added to the Methods section (page 7, lines 195–201).

Comment 2: Questionnaire Validation

No information is provided on the parameters of expert review (reliability, construct validity, consistency, or interpretability) for the novel questionnaire.

Response:

We have strengthened the reporting of instrument validation. The revised manuscript now includes details on the expert review process, including the criteria assessed (content relevance, clarity, interpretability, and comprehensiveness) and the feedback integration process. A pilot test was also conducted to assess internal consistency. These details have been added to the relevant section of the Methods (page 8, lines 234–244).

Comment 3: Informatics Perspective

Reliance on self-reported perceptions limits validation of actual healthcare-seeking behaviour. Integration of routine health information system data would strengthen the research.

Response:

We acknowledge this observation. This study deliberately relied on primary self-reported data because the objective was to capture individuals' perceived preferences and behavioural intentions, which are not fully observable in routine health records. Self-reported survey data are appropriate for understanding subjective decision-making, particularly where perceptions, accessibility, and socio-cultural factors drive behaviour. The integration of routine health information system data has been highlighted as a direction for future research in the revised manuscript.

Comment 4: Sample Size Calculation

The manuscript references Yamane's formula but does not report the population size or margin of error used.

Response:

The population size and margin of error (5%) used in Yamane's formula have now been explicitly reported in the revised Methods section (page 7, lines 214–215).

Comment 5: Overall Grammar and Style

Numerous grammatical and stylistic issues require editing, including inconsistent capitalization of healthcare facility names, inconsistent use of percentages and decimals, and inconsistent use of PHC as a concept versus system.

Response:

The manuscript has been thoroughly revised for grammar and style. Specifically: (1) 'Primary Healthcare' has been harmonized throughout; (2) PHC is now consistently used as a level of healthcare delivery; (3) abbreviations are written out at first use (including WHO at line 46); (4) reference formatting has been made consistent throughout; (5) line 62 and 71–72 have been revised for clarity; and (6) a professional language review has been undertaken. All flagged instances have been corrected in the revised manuscript.

REVIEWER #3

1. Statistical and Methodological Issues

Comment 1: Missing Bivariate Analysis

The title references 'Factors Associated with...' but no bivariate analysis (e.g., chi-square tests) was conducted prior to multivariable logistic regression.

Response:

We agree with this comment. Bivariate analyses (chi-square tests) were conducted to examine crude associations between predictor variables and facility preference prior to multivariable modelling. These results have now been incorporated into the Methods section and the relevant results table to justify predictor selection for the final model (page 8, lines 243–250).

Comment 2: Model Separation (Impossible Odds Ratios)

The Odds Ratio for the '61–83 yrs' age category is reported as 6.41×10^7 with missing confidence intervals, indicating complete or quasi-complete separation.

Response:

We thank the reviewer for this observation. The issue arose from sparse data in the 61–83 years age category. The correct figures have been provided and the tables updated accordingly.

Comment 3: Pilot Study Location Mismatch

The questionnaire was piloted in Kumasi, a highly urbanised setting, whereas the main study was conducted in Bolgatanga, a rural/semi-urban northern setting.

Response:

We acknowledge this limitation. The pilot was conducted in Kumasi primarily for logistical reasons and due to the availability of a comparable population of healthcare users. While we recognise the socio-cultural and geographic differences, the pilot was intended to assess instrument clarity, response consistency, and administration feasibility rather than contextual representativeness. This has been acknowledged revised manuscript. (Page 8, line 231-236)

Comment 4: Software Ambiguity

Line 228 references both Stata Version 17 and SPSS Version 22, but results appear to come from SPSS alone. Clarify which software was used for which analysis.

Response:

The Methods section has been revised to clearly specify that SPSS Version 22 was used for descriptive statistics and multinomial logistic regression, while Stata Version 17 was used for additional model diagnostics including VIF estimation. This distinction is now explicitly stated. Line 242-244

Comment 5: Model Diagnostics Clarification

Clarify to which specific models the reported VIF and pseudo-R^2 values apply.

Response:

The revised manuscript now explicitly states that the VIF values apply to the explanatory variables in the multinomial logistic regression model used to examine factors associated with facility preference, and that the pseudo-R^2 (Nagelkerke) pertains to the same model. These clarifications have been added to the Methods section (page 8).

<u>2. Structural and Formatting Issues</u>

Comment 6: Introduction Too Verbose

The introduction is too verbose; the theoretical model framework section should be reduced.

Response:

The introduction has been substantially tightened. Redundant background information has been removed and the theoretical framework section reduced to its essential elements, consistent with standard scientific writing conventions.

Comment 7: Methodology Repetition

The study design and population sections contain redundant repetitions.

Response:

Repetitive content in the study design and population sections has been consolidated (page 5).

Comment 8: Irrelevant Population Profile Details

Remove extraneous details (e.g., basketry, tomato farming) unless explicitly linked to occupational health-seeking behavior or income in the analysis.

Response:

Extraneous population profile details have been removed from the revised manuscript (page 5).

Comment 9: Table Restructuring (Tables 3 & 4)

Split Table 2 into two tables: one for descriptive analysis of Quality of Care and PHC Facility Type, and another for Factors Influencing Individual Preferences. Renumber subsequent tables accordingly.

Response:

Table 2 has been split into two distinct tables as recommended. The first focuses on descriptive analysis of Quality of Care and Preferred PHC Facility Type, and the second presents factors influencing individual preferences. The subsequent tables have been renumbered accordingly.

Comment 10: Conclusion Too Long

The conclusion contains redundant material that belongs in the Discussion section.

Response:

The conclusion has been revised and condensed to present only the main takeaways and implications of the study. Material more appropriate for the Discussion section has been relocated accordingly (page 21).

Comment 11: Reference Formatting

Verify consistency of all references; Line 407 incorrectly uses an author name instead of the required numbered citation format.

Response:

All references have been reviewed and formatted consistently. The error on Line 407 (Lederle et al.) has been corrected to the appropriate numbered citation format.

3. Specific Line-by-Line Edits

Comment 12: Line 19

Change 'the study' to 'this study'.

Response:

Corrected.

Comment 13: Line 141

Change 'and denied their consent' to 'patients who did not give consent'.

Response:

Corrected.

Comment 14: Line 164 – Figure Numbering

Text references 'Figure 2' but there is no Figure 1. Correct figure numbering sequentially.

Response:

Figure numbering has been corrected and is now sequential throughout the manuscript.

Comment 15: Line 228

Change 'Data were' to 'Data was' for consistency.

Response:

Corrected.

Comment 16: P-values in Tables 3, 4, 6, 7

A p-value of '0.000' is statistically impossible. All instances should read $p < 0.001$.

Response:

All instances of '0.000' in the tables have been changed to '$p < 0.001$'.

Comment 17: Table 3 Typo

Fix typographical error in Odds Ratio for the Cost variable, currently written as '0.76.07'.

Response:
The typographical error in Table 3 has been corrected.

We trust that the revisions adequately address all the concerns raised. We are grateful for the opportunity to improve the manuscript and hope it is now suitable for publication.

Sincerely,

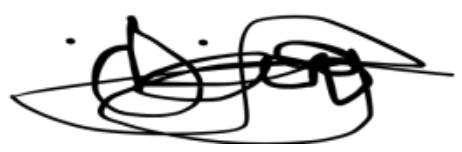

Mr. Charles Obeng (Corresponding Author 1)
Cc: Mrs. Sussana Atanga (Corresponding Author 2)
Prof. Kofi Akohene Mensah (PhD)
Mr. Lawal Alhassan
Tell: +233 (0) 550889312
