# Supplementary material for: Factors associated with individuals’ preferences for Primary Health Care facility type: A case of Bolgatanga Municipality in the Upper East Region of Ghana
Source: PLoS One. 2026 Aug 20;21(8):e0354800. doi: 10.1371/journal.pone.0354800 (PMC13492761; doi:10.1371/journal.pone.0354800)
Supplement: Supplementary file 4 [file pone.0354800.s001.docx]

**QUESTIONNAIRE**

TO IDENTIFY AND EXAMINE THE KEY FACTORS THAT INFLUENCE INDIVIDUAL PREFERENCES FOR PRIMARY HEALTHCARE FACILITY TYPE IN BOLGATANGA MUNICIPALITY, UPPER EAST REGION, GHANA

This research work seeks to identify and examine the key factors that influence individual preferences for primary healthcare facility type in Bolgatanga Municipality, Upper East Region, Ghana. This questionnaire is for academic purposes only. Respondents anonymous and any information gathered will remain confidential. Where alternatives have been provided in this questionnaire, please tick the appropriate response. For any other question write your answer in the space provided. Your participation and cooperation in this study is highly appreciated and valued. Thank you.

**SECTION A: Demographic Characteristics**

Please complete this part by ticking (√) the appropriate response to each statement.

1. Age

1. ▢ 18–29 years

2. ▢ 30–39 years

3. ▢ 40–49 years

4. ▢ 50–59 years

5. ▢ 60 years and above

2. What is your Gender?
    ▢ 1. Male
    ▢ 2. Female
    ▢ 3. Other (please specify): __________
3. What is your highest level of education completed?
    ▢ 1. No formal education
    ▢ 2. Primary school
    ▢ 3. Junior High School (JHS)
    ▢ 4. Senior High School (SHS)
    ▢ 5. Tertiary (Diploma, HND, Degree)
4. What is your current marital status?
    ▢ 1. Single
    ▢ 2. Married
    ▢ 3. Cohabiting
    ▢ 4. Divorced/Separated
    ▢ 5. Widowed
5. What is your religion?
    ▢ 1. Christianity
    ▢ 2. Islam
    ▢ 3. Traditional Religion
    ▢ 4. No religion
    ▢ 5. Other (please specify): __________
6. What is your current occupation?
    ▢ 1. Unemployed
    ▢ 2. Farmer
    ▢ 3. Trader
    ▢ 4. Civil/Public servant
    ▢ 5. Private sector worker
    ▢ 6. Artisan (e.g., carpenter, mason)
    ▢ 7. Student
    ▢ 8. Other (please specify): __________
7. What is your average monthly income?
    ▢ 1. Less than GHS 500
    ▢ 2. GHS 500 – 999
    ▢ 3. GHS 1,000 – 1,499
    ▢ 4. GHS 1,500 – 1,999
    ▢ 5. GHS 2,000 and above
    ▢ 6. Prefer not to say
8. NHIS Status

▢ 1. Not Registered
 ▢ 2. Registered but not active
 ▢ 3. Registered and active membership

**SECTION B**: **The Role of Healthcare Service Quality in Shaping Individual Preferences for Healthcare Facility Type.**

1. How important are the following factors when choosing a healthcare facility?

***Please complete this part by ticking (√) the appropriate response to each statement. The following five-point Likert-scale issued: (1) Not Important at All; 2) Not Important; 3) Neutral; 4) Important; 5) Very Important***

|  | **Factors when choosing a healthcare facility** | **1** | **2** | **3** | **4** | **5** |
| --- | --- | --- | --- | --- | --- | --- |
| 1 | Quality of care |  |  |  |  |  |
| 2 | Waiting time |  |  |  |  |  |
| 3 | Staff attitude and communication |  |  |  |  |  |
| 4 | Availability of follow-up care |  |  |  |  |  |
| 5 | Availability of specialized services |  |  |  |  |  |
| 6 | Cost |  |  |  |  |  |
| 7 | Availability of medicines |  |  |  |  |  |
| 8 | Clean environment |  |  |  |  |  |

2. How influential are the following factors in shaping your preference for a healthcare facility?

***Please complete this part by ticking (√) the appropriate response to each statement. The following five-point Likert-scale issued: (1) Not Influential at All; 2) Not Influential; 3) Neutral; 4) Influential; 5) Very Influential***

|  | **Factors shaping preference for healthcare facility** | **1** | **2** | **3** | **4** | **5** |
| --- | --- | --- | --- | --- | --- | --- |
| 1 | Personal recommendations |  |  |  |  |  |
| 2 | Online reviews |  |  |  |  |  |
| 3 | Facility reputation |  |  |  |  |  |
| 4 | Insurance coverage |  |  |  |  |  |
| 5 | Accessibility and location |  |  |  |  |  |

3. How would you rate the quality of care at your preferred healthcare facility?

1. ☐ Poor
2. ☐ Good
3. ☐ Fair
4. ☐ Very Good
5. ☐ Excellent

4. What type of healthcare facility do you prefer for?

1. ☐ Routine check-ups
2. ☐ Emergency care
3. ☐ Specialized treatment

5. Which of the following primary healthcare facility types do you usually prefer when seeking healthcare? *(Tick one)*

1. ☐ District hospital
2. ☐ Private clinic/hospital
3. ☐ Government health centers or polyclinic
4. ☐ CHPS compound
5. ☐ Faith-based health facility

***Please complete this part by ticking (√) the appropriate response to each statement. The following five-point Likert-scale issued: (1) Strongly Disagree; 2) Disagree; 3) Neutral; 4) Agree; 5) Strongly Agree***

|  | **Healthcare Service Quality** | **1** | **2** | **3** | **4** | **5** |
| --- | --- | --- | --- | --- | --- | --- |
| 1 | I do not wait long before receiving care anytime i visit the facility. |  |  |  |  |  |
| 2 | The facility is always clean and has neat environments that make me feel safe. |  |  |  |  |  |
| 3 | Healthcare workers at the facility explain my condition and treatment clearly and respectfully. |  |  |  |  |  |
| 4 | I was satisfied with the overall service I received during my visit to the facility |  |  |  |  |  |
| 5 | The facility usually has the prescribed medicines in stock after my consultation. |  |  |  |  |  |
| 6 | The quality of service at the facility influences my decision to use it over others. |  |  |  |  |  |

**SECTION C: Effect Of Accessibility Factors on Individual Preferences for Healthcare Facility Type.**

1. Have you used healthcare services in the past 6 months? (Yes/No)

1. ▢Yes
2. ▢No

2. What type of healthcare facility do you typically use? (Public, Private, Both)

1. ▢Public
2. ▢Private
3. ▢Both private and pubic

3. How does the location of a healthcare facility influence your decision to use it?

1. ▢ Not Influential at All
2. ▢ Not Influential
3. ▢ Neutral
4. ▢ Influential
5. ▢ Very Influential

4. Would you be willing to travel further for

a. Specialized care 1. Yes ▢	2.No ▢

b. Emergency care 1. Yes ▢	2.No ▢

c. Routine care	1. Yes ▢	2.No ▢

5. How important are the following factors when choosing a healthcare facility?

***Please complete this part by ticking (√) the appropriate response to each statement. The following five-point Likert-scale issued: (1) Not Important at All; 2) Not Important; 3) Neutral; 4) Important; 5) Very Important***

|  | **Accessibility** | **1** | **2** | **3** | **4** | **5** |
| --- | --- | --- | --- | --- | --- | --- |
| 1 | Distance/Location |  |  |  |  |  |
| 2 | Transportation options |  |  |  |  |  |
| 3 | Waiting time |  |  |  |  |  |
| 4 | Appointment availability |  |  |  |  |  |
| 5 | Cost |  |  |  |  |  |

6. Have you experienced any barriers to accessing healthcare services?

1. ▢Yes
2. ▢No

7. If yes, what were the barriers? (Select all that apply)

1. ▢Transportation issues
2. ▢Long waiting times
3. ▢Cost
4. ▢Distance
5. ▢Other (please specify)
