# Supplementary material for: Factors associated with individuals’ preferences for Primary Health Care facility type: A case of Bolgatanga Municipality in the Upper East Region of Ghana
Source: PLoS One. 2026 Aug 20;21(8):e0354800. doi: 10.1371/journal.pone.0354800 (PMC13492761; doi:10.1371/journal.pone.0354800)
Supplement: Supplementary file 5 [file pone.0354800.s003.docx]

# Review Report for PLOS ONE

**Manuscript Title:** Factors Associated with Individuals’ Preferences for Primary Healthcare Facility Type in Bolgatanga Municipality in the Upper East Region of Ghana.

Thank you for submitting this manuscript to PLOS ONE. The study addresses a relevant and underexplored question regarding determinants of primary healthcare facility preference in a semi-urban Ghanaian context. The use of Andersen’s Behavioral Model and multinomial logistic regression is conceptually appropriate. However, the manuscript requires substantial revision before it can be considered further. The comments below are organized by section and cover issues of methodology, statistical reporting, structure, and presentation.

**Recommendation:** Major Revision

## 1. Major Methodological & Statistical Concerns

- **Sample Size and Design Effect:** The sampling procedure described appears to be a **three-stage sampling design** (selection of sub-municipalities, compounds, and then households/adults), rather than the stated two-stage cluster sampling approach. In addition, the sample size was determined using Yamane's formula, which is based on simple random sampling assumptions. Since multistage cluster design was employed, the authors should justify why no design effect adjustment was considered and discuss the potential impact of clustering on statistical precision. Furthermore, the statement that “every household has an equal chance of being included” (Lines 215–216) is not supported by the sampling design. Sub-municipalities may differ substantially in socioeconomic status, education, ethnicity, and urbanization, and households located in compounds of different sizes may have unequal probabilities of selection. The authors should clarify the selection probabilities at each stage, justify the representativeness of the sample, and revise or qualify the claim of equal inclusion probability.
- **Missing Bivariate Analysis:** The title explicitly investigates "Factors Associated with..." However, there is no initial bivariate analysis (e.g., Chi-square tests). You should first test for crude associations between healthcare services/accessibility factors and preferences before jumping into multivariable logistic regression. Or if that was done, it should be stated in the methodology. This justifies your selection of predictors for the final model.
- **Model Separation (Impossible Odds Ratios):** In Tables 3 and 4, the Odds Ratio for the "61-83 yrs" category is reported as 6.41 x 10^7 with missing confidence intervals. This indicates complete or quasi-complete separation in your data (a sparse data problem).
- **Pilot Study Location Mismatch:** You piloted the questionnaire in Kumasi. Kumasi is a highly urbanized southern metropolis and is vastly different socio-culturally and geographically from the rural/semi-urban northern setting of Bolgatanga. You should justify why this pilot location was appropriate or acknowledge it as a severe limitation.
- **Software Ambiguity:** Line 228 states data was analyzed using Stata Version 17 and SPSS Version 22, but the results mention being pulled from SPSS. Clarify exactly which software was used for which analysis. Additionally, consider upgrading your software (SPSS v22 is over a decade old).
- **Clarification of Model Diagnostics:** Please explicitly clarify to which specific models the reported Variance Inflation Factor (VIF) and pseudo-R2 values apply.

## 2. Structural and Formatting Issues

- **Introduction and Framework:** The introduction is too verbose. You should adhere to standard scientific writing frameworks. Tighten the narrative. Furthermore, reduce the theoretical model framework section to its absolute necessities; remove redundant background information.
- **Methodology Repetition:** The study design and population sections contain redundant repetitions. Consolidate this information.
- **Irrelevant Population Profile:** Remove extraneous details from the population profile (e.g., basketry, tomato farming) unless you explicitly link them to occupational health-seeking behaviors or income in your analysis.
- **Table Titling (Tables 3 & 4):** Please split Table 2 into two distinct tables to improve readability and structural organization. Table 2 should focus exclusively on the descriptive analysis of Quality of Care and Preferred Primary Healthcare Facility Type. The variables representing the Factors Influencing Individual Preferences should be moved to a separate table, designated as Table 3. Then, Table 3 and 4 should be Table 4.
- **Conclusion:** The conclusion is too long and contains redundant material that belongs in the Discussion section. Keep the conclusion strictly to the main takeaways and implications.
- **Reference Formatting:** Verify that all references are formatted consistently. Consider using a reference manager. Specifically, Line 407 incorrectly uses an author name ("Lederle et al.") instead of the required numbered citation format.

## 3. Specific Line-by-Line Edits

- **Line 19:** Change "the study" to "this study".
- **Line 141:** Change "and denied their consent" to "patients who did not give consent".
- **Line 164 (Figure Numbering):** The text references "Figure 2", but there is no Figure 1. Correct the figure numbering sequentially.
- **Line 228:** Change "Data were" to "Data was" for consistency.
- **Tables 3, 4, 6, 7 (P-values):** A p-value of "0.000" is statistically impossible. Change all instances to p < 0.001.
- **Table 3 (Typo):** Fix the typographical error in the Odds Ratio for the Cost variable, currently written as "0.76.07".
