# Supplementary material for: Factors associated with individuals’ preferences for Primary Health Care facility type: A case of Bolgatanga Municipality in the Upper East Region of Ghana
Source: PLoS One. 2026 Aug 20;21(8):e0354800. doi: 10.1371/journal.pone.0354800 (PMC13492761; doi:10.1371/journal.pone.0354800)
Supplement: Supplementary file 6 [file pone.0354800.s007.docx]

**Response to Reviewers**

We thank the Editor and the Reviewers for their thorough and constructive review of our manuscript. We have carefully addressed each comment and revised the manuscript accordingly. Below, we provide a point-by-point response, indicating the specific page and line numbers where changes have been made, in line with Reviewer 1's request. All revisions are highlighted in the resubmitted manuscript.

**Editor's Comments**

*Thank you for the comment. The authors' comments below are consistent with, and respond jointly to, the related concerns raised by Reviewer 1 and Reviewer 2.*

**Comment: *1. The concerns regarding the sample size justification have not been fully addressed. Although the manuscript now acknowledges reduced statistical precision resulting from the achieved sample size, describing this limitation as "slight" is not supported given the difference between the planned and actual sample size. Please revise this section to accurately reflect the potential impact on the study's statistical precision and interpretability.***

**Author's Response:** We thank the reviewer for this observation and agree that the word "slight" understated the effect of the shortfall between the planned and achieved sample size. We have revised the limitations section to more accurately reflect the magnitude of this impact and its implications for statistical precision and interpretability. The revised text now reads (Page 7, Line 210):

*"* ...*the use of multistage cluster sampling may have introduced intra-cluster correlation, which could result in larger standard errors and reduced statistical precision."*

**Comment: *2. The statistical analyses also require greater completeness and transparency. The Methods state that bivariate analyses were performed; however, the corresponding results are not presented. Please include the bivariate analysis table referenced in the manuscript and ensure that the results are appropriately described in the text.***

**Author's Response:** We thank the reviewer for this comment. The bivariate analyses (Chi-square tests) have now been included in the manuscript. These analyses were performed for all variables that were subsequently entered into the multivariable logistic regression model, and the results are presented in Table 4 (Page 13) and Table 7 (Page 18). The corresponding narrative describing the bivariate findings has also been added, line 313 to 322 and 368 to 372 respectively.

**Comment: *3. Additionally, the descriptive characteristics table should include participants' age to provide a complete summary of the study population. Likewise, age should be included in the regression analysis table (or a clear justification provided if it was intentionally excluded from the model).***

**Author's Response:** We thank the reviewer for this comment. Age has now been included in both the descriptive characteristics table and the regression analysis table. We wish to clarify, for context, that in the previous round of review the Editors had requested that identifying data be removed or anonymised, noting: “We note that there is identifying data in Tables 1, 4. Please remove or anonymize all personal information age, ensure that the data shared are in accordance with participant consent, and add an anonymized version to your manuscript file.” In response to that earlier request, age had been analysed as a continuous variable. In light of the current comment, and consistent with the approach adopted by Bekele et al. (2026), who analysed age using categories in a study published in this journal, we have now included age and its corresponding categories in the descriptive and regression tables (Tables 1, 4 and 5).

**Reviewer #1**

**Comment: *Authors should also include the line numbers where to find the changes made and also what changes were made in the authors' comments; this helps the review process.***

**Author's Response:** We thank the reviewer for this helpful suggestion. We have revised our point-by-point responses throughout this document to clearly indicate the specific page and line numbers corresponding to each change, together with a description of what was changed, to facilitate the review process.

**Reviewer #3**

**Comment: *Please be mindful of extra full stops, missing spaces between the final word and the in-text citation number, and instances where present tense is used instead of past tense.***

**Author's Response:** We thank the reviewer for identifying these issues. Extra full stops, missing spaces before in-text citation numbers, and instances of present tense have been corrected throughout the manuscript, including at Lines 141, 159, 181, and 182, where tense and spacing errors have been rectified.

**Comment: *It's not necessary to restate the statistical software used here, as it has already been mentioned in line 252.***

**Author's Response:** We thank the reviewer for this observation. The repeated mention of the statistical software has been removed, as it had already been stated at Line 252. This has been rectified at Line 314.

**Comment: *N = 398, but the total of 610 indicates that this question allowed multiple responses. You may consider adding a footnote at the end of the table, in small italicised font, noting: *Totals may exceed the sample size because respondents could select more than one option. Although this is already stated within the table, including it as a footnote would improve clarity.***

**Author's Response:** We thank the reviewer for this helpful suggestion. A footnote has been added at the end of Table 6, in small italicised font, stating: "*Totals may exceed the sample size because respondents could select more than one option."

*We thank the Editor and Reviewers once again for their valuable comments, which have substantially strengthened the manuscript.*
