# Supplementary material for: Factors associated with individuals’ preferences for Primary Health Care facility type: A case of Bolgatanga Municipality in the Upper East Region of Ghana
Source: PLoS One. 2026 Aug 20;21(8):e0354800. doi: 10.1371/journal.pone.0354800 (PMC13492761; doi:10.1371/journal.pone.0354800)
Supplement: Peer review history

# Peer review: Decision Letter 0

**DOI:** 10.1371/journal.pone.0354800.r001

**Authors:** Mabel Kamweli Aworh

9 Jun 2026

Dear Dr. Obeng,

Thank you for submitting your manuscript to PLOS ONE. After careful consideration, we feel that it has merit but does not fully meet PLOS ONE’s publication criteria as it currently stands. Therefore, we invite you to submit a revised version of the manuscript that addresses the points raised during the review process.

Please submit your revised manuscript by Aug 08 2026 11:59PM. If you will need more time than this to complete your revisions, please reply to this message or contact the journal office at plosone@plos.org. When you're ready to submit your revision, log on to https://www.editorialmanager.com/pone/ and select the 'Submissions Needing Revision' folder to locate your manuscript file.

- A letter that responds to each point raised by the academic editor and reviewer(s). You should upload this letter as a separate file labeled 'Response to Reviewers'.
- A marked-up copy of your manuscript that highlights changes made to the original version. You should upload this as a separate file labeled 'Revised Manuscript with Track Changes'.
- An unmarked version of your revised paper without tracked changes. You should upload this as a separate file labeled 'Manuscript'.

If you would like to make changes to your financial disclosure, please include your updated statement in your cover letter. Guidelines for resubmitting your figure files are available below the reviewer comments at the end of this letter.

If applicable, we recommend that you deposit your laboratory protocols in protocols.io to enhance the reproducibility of your results. Protocols.io assigns your protocol its own identifier (DOI) so that it can be cited independently in the future. For instructions see: https://journals.plos.org/plosone/s/submission-guidelines#loc-laboratory-protocols. Additionally, PLOS ONE offers an option for publishing peer-reviewed Lab Protocol articles, which describe protocols hosted on protocols.io. Read more information on sharing protocols at https://plos.org/protocols?utm_medium=editorial-email&utm_source=authorletters&utm_campaign=protocols.

As the corresponding author, your ORCID iD is verified in the submission system and will appear in the published article. PLOS supports the use of ORCID, and we encourage all coauthors to register for an ORCID iD and use it as well. Please encourage your coauthors to verify their ORCID iD within the submission system before final acceptance, as unverified ORCID iDs will not appear in the published article. *Only*  the individual author can complete the verification step; PLOS staff *cannot*  verify ORCID iDs on behalf of authors.

We look forward to receiving your revised manuscript.

Kind regards,

Mabel Kamweli Aworh, DVM, MPH, PhD. FCVSN

Academic Editor

PLOS One

Journal Requirements:

When submitting your revision, we need you to address these additional requirements.

1. Please ensure that your manuscript meets PLOS ONE's style requirements, including those for file naming. The PLOS ONE style templates can be found at

https://journals.plos.org/plosone/s/file?id=wjVg/PLOSOne_formatting_sample_main_body.pdf and

https://journals.plos.org/plosone/s/file?id=ba62/PLOSOne_formatting_sample_title_authors_affiliations.pdf

2. In the online submission form, you indicated that “The datasets generated and/or analysed during the current study are not publicly available due to confidentiality and ethical restrictions involving participant information but are available from the corresponding author on reasonable request.”

All PLOS journals now require all data underlying the findings described in their manuscript to be freely available to other researchers, either 1. In a public repository, 2. Within the manuscript itself, or 3. Uploaded as supplementary information.

This policy applies to all data except where public deposition would breach compliance with the protocol approved by your research ethics board. If your data cannot be made publicly available for ethical or legal reasons (e.g., public availability would compromise patient privacy), please explain your reasons on resubmission and your exemption request will be escalated for approval.

3. Please ensure that you refer to Figure 2 in your text as, if accepted, production will need this reference to link the reader to the figure.

4. We note that Figure 1 in your submission contain [map/satellite] images which may be copyrighted. All PLOS content is published under the Creative Commons Attribution License (CC BY 4.0), which means that the manuscript, images, and Supporting Information files will be freely available online, and any third party is permitted to access, download, copy, distribute, and use these materials in any way, even commercially, with proper attribution. For these reasons, we cannot publish previously copyrighted maps or satellite images created using proprietary data, such as Google software (Google Maps, Street View, and Earth). For more information, see our copyright guidelines: http://journals.plos.org/plosone/s/licenses-and-copyright.

We require you to either (1) present written permission from the copyright holder to publish these figures specifically under the CC BY 4.0 license, or (2) remove the figures from your submission:

1. You may seek permission from the original copyright holder of Figure 2 to publish the content specifically under the CC BY 4.0 license.

We recommend that you contact the original copyright holder with the Content Permission Form (http://journals.plos.org/plosone/s/file?id=7c09/content-permission-form.pdf) and the following text:

“I request permission for the open-access journal PLOS ONE to publish XXX under the Creative Commons Attribution License (CCAL) CC BY 4.0 (http://creativecommons.org/licenses/by/4.0/). Please be aware that this license allows unrestricted use and distribution, even commercially, by third parties. Please reply and provide explicit written permission to publish XXX under a CC BY license and complete the attached form.”

Please upload the completed Content Permission Form or other proof of granted permissions as an ""Other"" file with your submission.

In the figure caption of the copyrighted figure, please include the following text: “Reprinted from [ref] under a CC BY license, with permission from [name of publisher], original copyright [original copyright year].”

2. If you are unable to obtain permission from the original copyright holder to publish these figures under the CC BY 4.0 license or if the copyright holder’s requirements are incompatible with the CC BY 4.0 license, please either i) remove the figure or ii) supply a replacement figure that complies with the CC BY 4.0 license. Please check copyright information on all replacement figures and update the figure caption with source information. If applicable, please specify in the figure caption text when a figure is similar but not identical to the original image and is therefore for illustrative purposes only.

The following resources for replacing copyrighted map figures may be helpful:

USGS National Map Viewer (public domain): http://viewer.nationalmap.gov/viewer/

The Gateway to Astronaut Photography of Earth (public domain): http://eol.jsc.nasa.gov/sseop/clickmap/

Maps at the CIA (public domain): https://www.cia.gov/library/publications/the-world-factbook/index.html and https://www.cia.gov/library/publications/cia-maps-publications/index.html

NASA Earth Observatory (public domain): http://earthobservatory.nasa.gov/

Landsat: http://landsat.visibleearth.nasa.gov/

USGS EROS (Earth Resources Observatory and Science (EROS) Center) (public domain): http://eros.usgs.gov/#

Natural Earth (public domain): http://www.naturalearthdata.com/

5. Please upload a copy of Supporting Information Figure/Table/etc. “supplementary file” which you refer to in your text on page 8.

6. If the reviewer comments include a recommendation to cite specific previously published works, please review and evaluate these publications to determine whether they are relevant and should be cited. There is no requirement to cite these works unless the editor has indicated otherwise.

**Additional Editor Comments:**

In addition to addressing the reviewers’ comments, please delete all subheadings from the discussion section to ensure a smoother flow.

[Note: HTML markup is below. Please do not edit.]

Reviewers' comments:

Reviewer's Responses to Questions

**Comments to the Author**

1. Is the manuscript technically sound, and do the data support the conclusions?

Reviewer #1: Yes

Reviewer #2: Yes

Reviewer #3: Yes

**********

2. Has the statistical analysis been performed appropriately and rigorously?

Reviewer #1: Yes

Reviewer #2: I Don't Know

Reviewer #3: Yes

**********

3. Have the authors made all data underlying the findings in their manuscript fully available?

The PLOS Data policy

Reviewer #1: No

Reviewer #2: Yes

Reviewer #3: No

**********

4. Is the manuscript presented in an intelligible fashion and written in standard English?

Reviewer #1: Yes

Reviewer #2: Yes

Reviewer #3: Yes

**********

Reviewer #1: Regarding the statistical analysis (multinomial logistic regression), please justify the selection of only quality of care, age, education, NHIS status, and cost among other sociodemographic variables.

Reviewer #2: Elucidating the toxicogenomic mechanisms of DBP exposure on Hepatocellular carcinoma through network toxicology and molecular docking analysis

Thank you for the opportunity to review the manuscript titled “Factors Associated with Individuals’ Preferences for Primary Healthcare Facility Type in Bolgatanga Municipality in the Upper East Region of Ghana.” The manuscript addresses a relevant public health issue and contributes local evidence regarding healthcare facility preferences in a municipal setting. The topic is timely and aligns with current efforts to strengthen primary healthcare utilization in Ghana. However, the manuscript would benefit from substantial revisions to improve methodological rigor, analytical transparency, interpretation of findings, and overall scientific clarity.

General Comments

Conceptualization of facility preference requires clarification: The manuscript seeks to examine determinants of preferences for PHC facility types, yet the facility categories included in the analysis raise conceptual concerns. District hospitals are categorized as primary healthcare facilities, whereas in Ghana's health system, they frequently function as secondary-level referral facilities. The authors should clearly justify why district hospitals were included as PHC facilities, whether respondents were asked about actual utilization or hypothetical preference, and how facility categories align with Ghana Health Service classifications. Failure to clarify this issue may affect the interpretation of the findings.

Questionnaire validation is insufficiently reported: The authors state that a novel questionnaire was developed and reviewed by five experts. However, no information is provided regarding the parameters of expert review, such as reliability testing, construct validity, consistency, or interpretability. Given that the study relies entirely on self-reported survey data, stronger evidence of instrument validity is required.

Informatics Perspective: An important opportunity exists to strengthen the manuscript through a health informatics lens. The reliance on self-reported perceptions limits the ability to validate actual healthcare-seeking behavior. Integration of routine health information system data would substantially strengthen future research.

Sample Size Calculation: The manuscript references Yamane's formula but does not provide the population size or the margin of error used for the calculation. These should be reported.

Overall grammar editing: Numerous grammatical and stylistic issues require editing. Specific examples are as below, but additional general observations include inconsistent capitalization of healthcare facility names and inconsistent use of percentages and decimals. A professional language edit is recommended.

1. Advised to harmonize the concept defined as PHC owing to several inconsistencies throughout the manuscript – Primary Health Care vs. Primary Healthcare.

2. PHC is confusingly alternated between being a concept and being a healthcare system.

3. Line 44-45: Consider revising the sentence to provide clarity for what context of PHC is mentioned here – a tier of the healthcare system or a concept of healthcare delivery.

4. Line 46: Write out abbreviations at first use. The abbreviations and acronyms can subsequently be used standing alone. The full definition of the acronym should precede the first mention of WHO in this line.

5. Line 62: Apply the correct reference style and stay consistent throughout the manuscript.

6. Line 71-72: Consider revising this statement to make it easier for the global audience to understand.

Figure Numbering: The manuscript begins with Figure 2, but Figure 1 is not presented. Please verify figure numbering.

Conclusion:

This study addresses a relevant policy and health systems question and provides potentially useful evidence regarding healthcare facility preferences within the Bolgatanga Municipality. However, significant concerns regarding grammar and editing, measurement validity, model interpretation, and analytical transparency must be addressed before the findings can be confidently interpreted. Strengthening these areas will substantially improve the manuscript's scientific rigor and contribution to the literature.

Reviewer #3: Your manuscript was an excellent and engaging read. I found the focus on PHC utilization preferences, particularly among facilities operating within the same geographical and policy context, to be both relevant and insightful, offering meaningful implications for resource allocation and the reduction of waste of limited resources. I have highlighted sections where further clarification or revision may be helpful, and I have embedded comments and recommendations directly in the PDF to ensure they are easy to locate and address. Thank you for the opportunity to review this work.

**********

what does this mean?). If published, this will include your full peer review and any attached files.

If you choose “no”, your identity will remain anonymous but your review may still be made public.

**Do you want your identity to be public for this peer review?**  For information about this choice, including consent withdrawal, please see our Privacy Policy

Reviewer #1: **Yes:** Oluwatomi Yetunde Oni

Reviewer #2: **Yes:** Babajide Adewumi

Reviewer #3: No

**********

[NOTE: If reviewer comments were submitted as an attachment file, they will be attached to this email and accessible via the submission site. Please log into your account, locate the manuscript record, and check for the action link "View Attachments". If this link does not appear, there are no attachment files.]

To ensure your figures meet our technical requirements, please review our figure guidelines: https://journals.plos.org/plosone/s/figures

You may also use PLOS’s free figure tool, NAAS, to help you prepare publication quality figures: https://journals.plos.org/plosone/s/figures#loc-tools-for-figure-preparation.

NAAS will assess whether your figures meet our technical requirements by comparing each figure against our figure specifications.

Attachment Submitted filename: Review Report for PLOS ONE (1).docx

Attachment Submitted filename: PONE-D-26-23521_Reviewed.pdf

# Author comment: Author response to Decision Letter 1

**DOI:** 10.1371/journal.pone.0354800.r002

15 Jun 2026

AUDITOR'S COMMENT

Comment:

Delete all subheadings from the Discussion section to ensure smoother flow.

Response:

All subheadings have been removed from the Discussion section. The content has been restructured into continuous prose to improve readability and flow.

REVIEWER #1

Comment:

Justify the selection of only quality of care, age, education, NHIS status, and cost in the multinomial logistic regression.

Response:

We appreciate this comment. Quality of care was the primary explanatory variable, directly aligned with the study's objective of examining its influence on facility preference. Age, education, NHIS status, and cost were included as control variables to adjust for potential confounding effects. These covariates were selected based on theoretical relevance and evidence from prior literature demonstrating their consistent influence on healthcare-seeking behaviour and facility utilisation decisions.

REVIEWER #2

General Comments

Comment 1: Conceptualization of Facility Preference

District hospitals are included as PHC facilities but often function as secondary-level referral facilities in Ghana. The authors should justify their inclusion and clarify whether questions concerned actual utilization or hypothetical preference, and how facility categories align with Ghana Health Service (GHS) classifications.

Response:

We acknowledge this concern. In the Ghanaian health system context, district hospitals serve dual roles, often functioning as the primary point of care in districts with limited health centre coverage. For this study, PHC facility types were defined based on the functional and accessible role they play in the community, consistent with how GHS operationalises first-contact care at the district level. Additionally, the questionnaire focused on respondents' preferences for healthcare facility use rather than referral utilization. This clarification has been added to the Methods section (page 7, lines 195–201).

Comment 2: Questionnaire Validation

No information is provided on the parameters of expert review (reliability, construct validity, consistency, or interpretability) for the novel questionnaire.

Response:

We have strengthened the reporting of instrument validation. The revised manuscript now includes details on the expert review process, including the criteria assessed (content relevance, clarity, interpretability, and comprehensiveness) and the feedback integration process. A pilot test was also conducted to assess internal consistency. These details have been added to the relevant section of the Methods (page 8, lines 234–244).

Comment 3: Informatics Perspective

Reliance on self-reported perceptions limits validation of actual healthcare-seeking behaviour. Integration of routine health information system data would strengthen the research.

Response:

We acknowledge this observation. This study deliberately relied on primary self-reported data because the objective was to capture individuals' perceived preferences and behavioural intentions, which are not fully observable in routine health records. Self-reported survey data are appropriate for understanding subjective decision-making, particularly where perceptions, accessibility, and socio-cultural factors drive behaviour. The integration of routine health information system data has been highlighted as a direction for future research in the revised manuscript.

Comment 4: Sample Size Calculation

The manuscript references Yamane's formula but does not report the population size or margin of error used.

Response:

The population size and margin of error (5%) used in Yamane's formula have now been explicitly reported in the revised Methods section (page 7, lines 214–215).

Comment 5: Overall Grammar and Style

Numerous grammatical and stylistic issues require editing, including inconsistent capitalization of healthcare facility names, inconsistent use of percentages and decimals, and inconsistent use of PHC as a concept versus system.

Response:

The manuscript has been thoroughly revised for grammar and style. Specifically: (1) 'Primary Healthcare' has been harmonized throughout; (2) PHC is now consistently used as a level of healthcare delivery; (3) abbreviations are written out at first use (including WHO at line 46); (4) reference formatting has been made consistent throughout; (5) line 62 and 71–72 have been revised for clarity; and (6) a professional language review has been undertaken. All flagged instances have been corrected in the revised manuscript.

REVIEWER #3

1. Statistical and Methodological Issues

Comment 1: Missing Bivariate Analysis

The title references 'Factors Associated with...' but no bivariate analysis (e.g., chi-square tests) was conducted prior to multivariable logistic regression.

Response:

We agree with this comment. Bivariate analyses (chi-square tests) were conducted to examine crude associations between predictor variables and facility preference prior to multivariable modelling. These results have now been incorporated into the Methods section and the relevant results table to justify predictor selection for the final model (page 8, lines 243–250).

Comment 2: Model Separation (Impossible Odds Ratios)

The Odds Ratio for the '61–83 yrs' age category is reported as 6.41 × 10⁷ with missing confidence intervals, indicating complete or quasi-complete separation.

Response:

We thank the reviewer for this observation. The issue arose from sparse data in the 61–83 years age category. The correct figures have been provided and the tables updated accordingly.

Comment 3: Pilot Study Location Mismatch

The questionnaire was piloted in Kumasi, a highly urbanised setting, whereas the main study was conducted in Bolgatanga, a rural/semi-urban northern setting.

Response:

We acknowledge this limitation. The pilot was conducted in Kumasi primarily for logistical reasons and due to the availability of a comparable population of healthcare users. While we recognise the socio-cultural and geographic differences, the pilot was intended to assess instrument clarity, response consistency, and administration feasibility rather than contextual representativeness. This has been acknowledged revised manuscript. (Page 8, line 231-236)

Comment 4: Software Ambiguity

Line 228 references both Stata Version 17 and SPSS Version 22, but results appear to come from SPSS alone. Clarify which software was used for which analysis.

Response:

The Methods section has been revised to clearly specify that SPSS Version 22 was used for descriptive statistics and multinomial logistic regression, while Stata Version 17 was used for additional model diagnostics including VIF estimation. This distinction is now explicitly stated. Line 242-244

Comment 5: Model Diagnostics Clarification

Clarify to which specific models the reported VIF and pseudo-R² values apply.

Response:

The revised manuscript now explicitly states that the VIF values apply to the explanatory variables in the multinomial logistic regression model used to examine factors associated with facility preference, and that the pseudo-R² (Nagelkerke) pertains to the same model. These clarifications have been added to the Methods section (page 8).

2. Structural and Formatting Issues

Comment 6: Introduction Too Verbose

The introduction is too verbose; the theoretical model framework section should be reduced.

Response:

The introduction has been substantially tightened. Redundant background information has been removed and the theoretical framework section reduced to its essential elements, consistent with standard scientific writing conventions.

Comment 7: Methodology Repetition

The study design and population sections contain redundant repetitions.

Response:

Repetitive content in the study design and population sections has been consolidated (page 5).

Comment 8: Irrelevant Population Profile Details

Remove extraneous details (e.g., basketry, tomato farming) unless explicitly linked to occupational health-seeking behavior or income in the analysis.

Response:

Extraneous population profile details have been removed from the revised manuscript (page 5).

Comment 9: Table Restructuring (Tables 3 & 4)

Split Table 2 into two tables: one for descriptive analysis of Quality of Care and PHC Facility Type, and another for Factors Influencing Individual Preferences. Renumber subsequent tables accordingly.

Response:

Table 2 has been split into two distinct tables as recommended. The first focuses on descriptive analysis of Quality of Care and Preferred PHC Facility Type, and the second presents factors influencing individual preferences. The subsequent tables have been renumbered accordingly.

Comment 10: Conclusion Too Long

The conclusion contains redundant material that belongs in the Discussion section.

Response:

The conclusion has been revised and condensed to present only the main takeaways and implications of the study. Material more appropriate for the Discussion section has been relocated accordingly (page 21).

Comment 11: Reference Formatting

Verify consistency of all references; Line 407 incorrectly uses an author name instead of the required numbered citation format.

Response:

All references have been reviewed and formatted consistently. The error on Line 407 (Lederle et al.) has been corrected to the appropriate numbered citation format.

3. Specific Line-by-Line Edits

Comment 12: Line 19

Change 'the study' to 'this study'.

Response:

Corrected.

Comment 13: Line 141

Change 'and denied their consent' to 'patients who did not give consent'.

Response:

Corrected.

Comment 14: Line 164 – Figure Numbering

Text references 'Figure 2' but there is no Figure 1. Correct figure numbering sequentially.

Response:

Figure numbering has been corrected and is now sequential throughout the manuscript.

Comment 15: Line 228

Change 'Data were' to 'Data was' for consistency.

Response:

Corrected.

Comment 16: P-values in Tables 3, 4, 6, 7

A p-value of '0.000' is statistically impossible. All instances should read p < 0.001.

Response:

All instances of '0.000' in the tables have been changed to 'p < 0.001'.

Comment 17: Table 3 Typo

Fix typographical error in Odds Ratio for the Cost variable, currently written as '0.76.07'.

Response:

The typographical error in Table 3 has been corrected.

Attachment Submitted filename: Response to Reviewers.pdf

# Peer review: Decision Letter 1

**DOI:** 10.1371/journal.pone.0354800.r003

**Authors:** Mabel Kamweli Aworh

6 Jul 2026

Thank you for submitting your manuscript to PLOS ONE. After careful consideration, we feel that it has merit but does not fully meet PLOS ONE’s publication criteria as it currently stands. Therefore, we invite you to submit a revised version of the manuscript that addresses the points raised during the review process.

Please submit your revised manuscript by Sep 04 2026 11:59PM. If you will need more time than this to complete your revisions, please reply to this message or contact the journal office at plosone@plos.org. When you're ready to submit your revision, log on to https://www.editorialmanager.com/pone/ and select the 'Submissions Needing Revision' folder to locate your manuscript file.

- A letter that responds to each point raised by the academic editor and reviewer(s). You should upload this letter as a separate file labeled 'Response to Reviewers'.
- A marked-up copy of your manuscript that highlights changes made to the original version. You should upload this as a separate file labeled 'Revised Manuscript with Track Changes'.
- An unmarked version of your revised paper without tracked changes. You should upload this as a separate file labeled 'Manuscript'.

If you would like to make changes to your financial disclosure, please include your updated statement in your cover letter. Guidelines for resubmitting your figure files are available below the reviewer comments at the end of this letter.

If applicable, we recommend that you deposit your laboratory protocols in protocols.io to enhance the reproducibility of your results. Protocols.io assigns your protocol its own identifier (DOI) so that it can be cited independently in the future. For instructions see: https://journals.plos.org/plosone/s/submission-guidelines#loc-laboratory-protocols. Additionally, PLOS ONE offers an option for publishing peer-reviewed Lab Protocol articles, which describe protocols hosted on protocols.io. Read more information on sharing protocols at https://plos.org/protocols?utm_medium=editorial-email&utm_source=authorletters&utm_campaign=protocols.

As the corresponding author, your ORCID iD is verified in the submission system and will appear in the published article. PLOS supports the use of ORCID, and we encourage all coauthors to register for an ORCID iD and use it as well. Please encourage your coauthors to verify their ORCID iD within the submission system before final acceptance, as unverified ORCID iDs will not appear in the published article. *Only*  the individual author can complete the verification step; PLOS staff *cannot*  verify ORCID iDs on behalf of authors.

We look forward to receiving your revised manuscript.

Kind regards,

Mabel Kamweli Aworh, DVM, MPH, PhD. FCVSN

Academic Editor

PLOS One

Journal Requirements:

If the reviewer comments include a recommendation to cite specific previously published works, please review and evaluate these publications to determine whether they are relevant and should be cited. There is no requirement to cite these works unless the editor has indicated otherwise.

Please review your reference list to ensure that it is complete and correct. If you have cited papers that have been retracted, please include the rationale for doing so in the manuscript text, or remove these references and replace them with relevant current references. Any changes to the reference list should be mentioned in the rebuttal letter that accompanies your revised manuscript. If you need to cite a retracted article, indicate the article’s retracted status in the References list and also include a citation and full reference for the retraction notice.

**Additional Editor Comments:**

- The concerns regarding the sample size justification have not been fully addressed. Although the manuscript now acknowledges reduced statistical precision resulting from the achieved sample size, describing this limitation as "slight" is not supported given the difference between the planned and actual sample size. Please revise this section to accurately reflect the potential impact on the study's statistical precision and interpretability.
- The statistical analyses also require greater completeness and transparency. The Methods state that bivariate analyses were performed; however, the corresponding results are not presented. Please include the bivariate analysis table referenced in the manuscript and ensure that the results are appropriately described in the text.
- Additionally, the descriptive characteristics table should include participants' age to provide a complete summary of the study population. Likewise, age should be included in the regression analysis table (or a clear justification provided if it was intentionally excluded from the model).
- Finally, the manuscript would benefit from additional editing to improve clarity and conciseness.

Please ensure that all reviewer comments have been fully addressed and that the revised manuscript, response to reviewers, and supporting tables are consistent with one another.

[Note: HTML markup is below. Please do not edit.]

Reviewers' comments:

Reviewer's Responses to Questions

**Comments to the Author**

Reviewer #1: (No Response)

Reviewer #2: All comments have been addressed

Reviewer #3: (No Response)

**********

2. Is the manuscript technically sound, and do the data support the conclusions?

Reviewer #1: (No Response)

Reviewer #2: Yes

Reviewer #3: Yes

**********

3. Has the statistical analysis been performed appropriately and rigorously?

Reviewer #1: (No Response)

Reviewer #2: Yes

Reviewer #3: Yes

**********

4. Have the authors made all data underlying the findings in their manuscript fully available?

The PLOS Data policy

Reviewer #1: (No Response)

Reviewer #2: Yes

Reviewer #3: (No Response)

**********

5. Is the manuscript presented in an intelligible fashion and written in standard English?

Reviewer #1: (No Response)

Reviewer #2: Yes

Reviewer #3: Yes

**********

Reviewer #1: Review Report for PLOS ONE

Manuscript Title: Factors Associated with Individuals’ Preferences for Primary Healthcare Facility Type in Bolgatanga Municipality in the Upper East Region of Ghana.

RE-AUTHOR’S COMMENTS

• The initial comment about the sample size and design effect was not addressed in the comment, however, I noticed the authors addressed it in the manuscript but said “slightly reduced statistical precision”. Considering the gap in sample size the reduction in statistical precision is not “slight”, remove it.

• Regarding the bivariate analysis table, authors mentioned they included the statement of conducting a bivariate analysis in the methods, but the table was not included in the manuscript, include it.

• In the descriptive table the descriptive analysis for age was not included.

• Furthermore, the table with the odds ratio, doesn’t have age listed there.

• Manuscript as is, is still quite verbose

Generally.

• Authors should consolidate/compile all of the changes they made, properly, before submitting as this aids readability and review.

• Authors should also include the line numbers where to find the changes made and also what changes were made in the authors comments, helps the review process.

Reviewer #2: Thank you for the opportunity to review the manuscript titled “Factors Associated with Individuals’ Preferences for Primary Healthcare Facility Type in Bolgatanga Municipality in the Upper East Region of Ghana.” The authors have addressed the concerns highlighted in the previous reviews. Appropriate statistical review and analytical rigors have been applied. This topic opens potential opportunities for future research in the space and contribution to literature.

Reviewer #3: Well done to all the authors for providing this revised draft of the manuscript. There is a clear improvement in the write‑up, it is more detailed and reflects greater thoughtfulness and hard work. I have a few suggestions: please be mindful of extra full stops, missing spaces between the final word and the in‑text citation number, and instances where present tense is used instead of past tense. I have also included additional recommendations to further strengthen the quality of the manuscript. Kindly refer to the yellow highlights, which point to my suggested revisions.

**********

what does this mean?). If published, this will include your full peer review and any attached files.

If you choose “no”, your identity will remain anonymous but your review may still be made public.

**Do you want your identity to be public for this peer review?**  For information about this choice, including consent withdrawal, please see our Privacy Policy

Reviewer #1: **Yes:** Oluwatomi Oni

Reviewer #2: **Yes:** Babajide Adewumi

Reviewer #3: No

**********

[NOTE: If reviewer comments were submitted as an attachment file, they will be attached to this email and accessible via the submission site. Please log into your account, locate the manuscript record, and check for the action link "View Attachments". If this link does not appear, there are no attachment files.]

To ensure your figures meet our technical requirements, please review our figure guidelines: https://journals.plos.org/plosone/s/figures

You may also use PLOS’s free figure tool, NAAS, to help you prepare publication quality figures: https://journals.plos.org/plosone/s/figures#loc-tools-for-figure-preparation.

NAAS will assess whether your figures meet our technical requirements by comparing each figure against our figure specifications.

Attachment Submitted filename: PONE-D-26-23521_R1.pdf

# Author comment: Author response to Decision Letter 2

**DOI:** 10.1371/journal.pone.0354800.r004

8 Jul 2026

We thank the Editor and the Reviewers for their thorough and constructive review of our manuscript. We have carefully addressed each comment and revised the manuscript accordingly. Below, we provide a point-by-point response, indicating the specific page and line numbers where changes have been made, in line with Reviewer 1's request. All revisions are highlighted in the resubmitted manuscript.

Editor's Comments

Thank you for the comment. The authors' comments below are consistent with, and respond jointly to, the related concerns raised by Reviewer 1 and Reviewer 2.

Comment: 1. The concerns regarding the sample size justification have not been fully addressed. Although the manuscript now acknowledges reduced statistical precision resulting from the achieved sample size, describing this limitation as "slight" is not supported given the difference between the planned and actual sample size. Please revise this section to accurately reflect the potential impact on the study's statistical precision and interpretability.

Author's Response: We thank the reviewer for this observation and agree that the word "slight" understated the effect of the shortfall between the planned and achieved sample size. We have revised the limitations section to more accurately reflect the magnitude of this impact and its implications for statistical precision and interpretability. The revised text now reads (Page 7, Line 210):

" ...the use of multistage cluster sampling may have introduced intra-cluster correlation, which could result in larger standard errors and reduced statistical precision."

Comment: 2. The statistical analyses also require greater completeness and transparency. The Methods state that bivariate analyses were performed; however, the corresponding results are not presented. Please include the bivariate analysis table referenced in the manuscript and ensure that the results are appropriately described in the text.

Author's Response: We thank the reviewer for this comment. The bivariate analyses (Chi-square tests) have now been included in the manuscript. These analyses were performed for all variables that were subsequently entered into the multivariable logistic regression model, and the results are presented in Table 4 (Page 13) and Table 7 (Page 18). The corresponding narrative describing the bivariate findings has also been added, line 313 to 322 and 368 to 372 respectively.

Comment: 3. Additionally, the descriptive characteristics table should include participants' age to provide a complete summary of the study population. Likewise, age should be included in the regression analysis table (or a clear justification provided if it was intentionally excluded from the model).

Author's Response: We thank the reviewer for this comment. Age has now been included in both the descriptive characteristics table and the regression analysis table. We wish to clarify, for context, that in the previous round of review the Editors had requested that identifying data be removed or anonymised, noting: “We note that there is identifying data in Tables 1, 4. Please remove or anonymize all personal information age, ensure that the data shared are in accordance with participant consent, and add an anonymized version to your manuscript file.” In response to that earlier request, age had been analysed as a continuous variable. In light of the current comment, and consistent with the approach adopted by Bekele et al. (2026), who analysed age using categories in a study published in this journal, we have now included age and its corresponding categories in the descriptive and regression tables (Tables 1, 4 and 5).

Reviewer #1

Comment: Authors should also include the line numbers where to find the changes made and also what changes were made in the authors' comments; this helps the review process.

Author's Response: We thank the reviewer for this helpful suggestion. We have revised our point-by-point responses throughout this document to clearly indicate the specific page and line numbers corresponding to each change, together with a description of what was changed, to facilitate the review process.

Reviewer #3

Comment: Please be mindful of extra full stops, missing spaces between the final word and the in-text citation number, and instances where present tense is used instead of past tense.

Author's Response: We thank the reviewer for identifying these issues. Extra full stops, missing spaces before in-text citation numbers, and instances of present tense have been corrected throughout the manuscript, including at Lines 141, 159, 181, and 182, where tense and spacing errors have been rectified.

Comment: It's not necessary to restate the statistical software used here, as it has already been mentioned in line 252.

Author's Response: We thank the reviewer for this observation. The repeated mention of the statistical software has been removed, as it had already been stated at Line 252. This has been rectified at Line 314.

Comment: N = 398, but the total of 610 indicates that this question allowed multiple responses. You may consider adding a footnote at the end of the table, in small italicised font, noting: *Totals may exceed the sample size because respondents could select more than one option. Although this is already stated within the table, including it as a footnote would improve clarity.

Author's Response: We thank the reviewer for this helpful suggestion. A footnote has been added at the end of Table 6, in small italicised font, stating: "*Totals may exceed the sample size because respondents could select more than one option."

Attachment Submitted filename: Response to Reviewers (1).docx

# Editor report: Decision Letter 2

**DOI:** 10.1371/journal.pone.0354800.r005

**Authors:** Mabel Kamweli Aworh

14 Jul 2026

Factors Associated with Individuals’ Preferences for Primary Health Care Facility Type: A Case of Bolgatanga Municipality in the Upper East Region of Ghana

PONE-D-26-23521R2

Dear Dr. Obeng,

We’re pleased to inform you that your manuscript has been judged scientifically suitable for publication and will be formally accepted for publication once it meets all outstanding technical requirements.

Within one week, you’ll receive an e-mail detailing the required amendments. When these have been addressed, you’ll receive a formal acceptance letter and your manuscript will be scheduled for publication.

An invoice will be generated when your article is formally accepted. Please note, if your institution has a publishing partnership with PLOS and your article meets the relevant criteria, all or part of your publication costs will be covered. Please make sure your user information is up-to-date by logging into Editorial Manager at Editorial Manager® and clicking the ‘Update My Information' link at the top of the page. For questions related to billing, please contact billing support.

If your institution or institutions have a press office, please notify them about your upcoming paper to help maximize its impact. If they’ll be preparing press materials, please inform our press team as soon as possible -- no later than 48 hours after receiving the formal acceptance. Your manuscript will remain under strict press embargo until 2 pm Eastern Time on the date of publication. For more information, please contact onepress@plos.org.

Kind regards,

Mabel Kamweli Aworh, DVM, MPH, PhD. FCVSN

Academic Editor

PLOS One

Additional Editor Comments (optional):

Reviewers' comments:

# Editor report: Acceptance letter

**DOI:** 10.1371/journal.pone.0354800.r006

**Authors:** Mabel Kamweli Aworh

PONE-D-26-23521R2

PLOS One

Dear Dr. Obeng,

I'm pleased to inform you that your manuscript has been deemed suitable for publication in PLOS One. Congratulations! Your manuscript is now being handed over to our production team.

At this stage, our production department will prepare your paper for publication. This includes ensuring the following:

* All references, tables, and figures are properly cited

* All relevant supporting information is included in the manuscript submission,

* There are no issues that prevent the paper from being properly typeset

You will receive further instructions from the production team, including instructions on how to review your proof when it is ready. Please keep in mind that we are working through a large volume of accepted articles, so please give us a few days to review your paper and let you know the next and final steps.

Lastly, if your institution or institutions have a press office, please let them know about your upcoming paper now to help maximize its impact. If they'll be preparing press materials, please inform our press team within the next 48 hours. Your manuscript will remain under strict press embargo until 2 pm Eastern Time on the date of publication. For more information, please contact onepress@plos.org.

You will receive an invoice from PLOS for your publication fee after your manuscript has reached the completed accept phase. If you receive an email requesting payment before acceptance or for any other service, this may be a phishing scheme. Learn how to identify phishing emails and protect your accounts at https://explore.plos.org/phishing.

If we can help with anything else, please email us at customercare@plos.org.

Thank you for submitting your work to PLOS One and supporting open access.

Kind regards,

PLOS ONE Editorial Office Staff

on behalf of

Dr. Mabel Kamweli Aworh

Academic Editor

PLOS One
